# Iron and Other Metal Ions in Human Health and Disease

**DOI:** 10.1002/mco2.71021

**Published:** 2026-10-02

**Authors:** Xiaofeng Dai, Jitian Li

**Affiliations:** ^1^ Department of Geriatric Endocrinology and Metabolism, International Ward 1, National Local Joint Engineering Research Center For Precision Surgery & Regenerative Medicine, Shaanxi Provincial Center for Regenerative Medicine and Surgical Engineering The First Affiliated Hospital of Xi'an Jiaotong University Xi'an China; ^2^ Clinical Medical Center of Tissue Engineering and Regeneration, The Third Affiliated Hospital of Henan Medical University Henan Medical University Xinxiang China

**Keywords:** calcium, copper, iron, redox‐active metals, redox‐inert metals, zinc

## Abstract

Iron is the most abundant trace element, driving both the commonest deficiency and ferroptosis. This review takes iron as the central theme and extends to copper, zinc, and calcium, spanning physiological functions, pathological mechanisms, risk factors, diagnostics, and therapeutics. We classify these ions into redox‐active (iron, copper), which engage in catalytic electron transfer, and redox‐inert (zinc, calcium), which serve structural and signaling roles without redox cycling. Iron deficiency remains the global leading micronutrient deficit, whereas iron overload triggers ferroptosis‐mediated organ damage. Copper deficiency manifests as hematological and neurological syndromes, while copper excess induces cuproptosis. Zinc deficiency impairs growth and immunity, yet chronic zinc toxicity primarily causes secondary copper deficiency. Calcium dysregulation underpins hypoparathyroidism, osteoporosis, cardiovascular diseases, and crystal deposition disorders. Diagnosis integrates clinical assessment, biochemical profiling, imaging, and genetic analysis; risk factors encompass dietary insufficiency, environmental exposure, genetic variants, and demographic variables. Given the narrow therapeutic window between deficiency and toxicity, vigilant monitoring is mandatory. Treatment strategies are metal‐specific and include chelation, hormone replacement, source removal, and channel modulation. Future directions emphasize validated biomarkers, tissue‐targeted delivery, and metallo‐genomics to enable precision medicine, with iron metabolism as a pivotal entry point for understanding and managing systemic metal homeostasis.

## Graphical abstract

Iron, copper, zinc, and calcium orchestrate cellular function through distinct yet cooperative mechanisms: redox‐active iron and copper cycle between oxidation states to act as Fenton catalysts and electron carriers in oxidative phosphorylation (OXPHOS); redox‐inert zinc serves as a structural component of zinc‐finger proteins and a catalytic cofactor for >300 enzymes; and calcium functions as a universal second messenger. OXPHOS uniquely integrates all four metals: iron and copper facilitate electron transfer within respiratory chain complexes, zinc supports zinc‐finger regulators such as NDUFS6 and the catalytic activity of SOD1, and calcium activates TCA cycle dehydrogenases via MCU to couple energy demand with ATP production. Imbalance between redox‐active and redox‐inert metal ions disrupts energy metabolism and ROS homeostasis, driving either proliferative conditions like cancer, or metal‐induced cell death pathways like ferroptosis, cuproptosis, and apoptosis toward degenerative syndromes such as Alzheimer's disease.

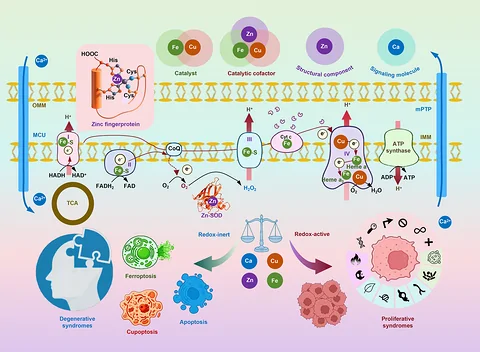

## 1 Introduction

Among the myriad of metal ions essential for human life, iron (Fe) holds a unique and preeminent position. As the most abundant transition metal in the human body, Fe is a critical cofactor in oxygen transport (hemoglobin), electron transfer (cytochromes), and numerous enzymatic reactions, and its discovery as a functional component of hemoglobin over a century ago marked the dawn of metallobiology [1, 2]. Yet Fe is far from alone in its biological significance; other metal ions, notably copper (Cu), zinc (Zn), and calcium (Ca), are equally indispensable, each contributing distinct chemical properties that underpin a vast array of physiological processes. Over the past decades, advances in bioinorganic chemistry, structural biology, and metallomics have revealed that metal ions participate in virtually every cellular process, from catalyzing redox reactions and stabilizing protein folds to transmitting electrical signals and regulating gene expression [3, 4]. Approximately one‐third of all proteins require a metal cofactor for proper function, underscoring the profound integration of these elements into the core machinery of cellular metabolism, signaling, and stress responses [5, 6]. Despite this foundational knowledge, the full spectrum of metal ion functions, particularly their roles in disease pathogenesis and as therapeutic targets, remains an active frontier of biomedical research.

The myriad of metal elements present in the human body can be broadly categorized into redox‐active and redox‐inert metals based on their electronic configurations and chemical behavior. Redox‐active metal ions, such as Fe and Cu, are characterized by partially filled d‐orbitals that enable participation in one‐electron transfer reactions [7, 8]. Redox‐inert metal ions, including Zn and Ca, lack this redox activity but excel in structural and regulatory functions [9, 10]. The unique chemical versatility of redox‐active metals is both biologically advantageous and potentially hazardous. On one hand, it enables essential redox chemistry in processes such as electron transport, oxygen carriage, and enzymatic catalysis; on the other hand, dysregulated electron transfer can generate damaging reactive oxygen species (ROS) via Fenton or Fenton‐like reactions, a risk particularly pronounced for Fe, given its abundance and potent catalytic activity [7, 11]. Redox‐inert metals, in contrast, provide stable structural and signaling functions without engaging in potentially harmful redox cycling [9, 10]. Understanding the physiological roles of these metal ions provides the necessary foundation for appreciating how their dysregulation leads to disease.

When metal homeostasis is disrupted, whether through deficiency, overload, or mislocalization, profound pathological consequences ensue [9, 12]. Among all metal‐related disorders, Fe imbalance stands out as both the most common and the most extensively studied. Fe deficiency remains the most prevalent micronutrient deficiency globally, affecting approximately one in four individuals worldwide [13, 14], while Fe overload, as seen in hereditary hemochromatosis (HH) and transfusion‐dependent anemias, causes progressive multiorgan damage through oxidative stress and, notably, ferroptosis, an Fe‐dependent form of regulated cell death [15, 16]. Cu deficiency and overload, though rarer, produce equally devastating neurological and hepatic syndromes, and excess Cu triggers cuproptosis, a distinct cell death pathway [17, 18]. Zn imbalance contributes to growth retardation, immune dysfunction, and neurodevelopmental disorders [19, 20], and chronic Zn toxicity often manifests as secondary Cu deficiency [21]. Ca dysregulation underpins a wide spectrum of conditions ranging from osteoporosis and cardiac arrhythmias to neurodegenerative diseases and crystal deposition disorders [22, 23]. Beyond these individual pathologies, metal ions have been increasingly recognized as central regulators of cell death pathways. The discoveries of ferroptosis (Fe‐dependent) and cuproptosis (Cu‐dependent), along with emerging roles of Zn and Ca in programmed cell death, have fundamentally reshaped our understanding of how metal dysregulation drives tissue injury and inflammation [24, 25, 26, 27]. This integrated view positions metal ions, and particularly Fe, given its central role in both metabolic and death signaling, as critical nodes linking metabolic dysfunction, oxidative stress, and inflammation across a broad range of human diseases [28, 29].

Despite significant advances, several critical knowledge gaps remain. The mechanisms governing tissue‐specific susceptibility to metal toxicity, the interplay between different metals in disease pathogenesis, and the translation of mechanistic insights into effective therapies all require further investigation. Moreover, while individual metal‐related disorders have been extensively studied, a comprehensive synthesis that integrates the physiological roles, pathological associations, risk factors, diagnostic approaches, and therapeutic strategies across multiple essential metals (Fe, Cu, Zn, and Ca) is currently lacking. This review aims to fill this gap by providing an integrated, expert‐driven synthesis that bridges molecular biology, clinical medicine, and systems toxicology, with Fe as the central focus and the other three metals serving as complementary paradigms.

Using Fe [30, 31], Cu [18], Zn [21], and Ca [12, 32] as primary examples, given their abundance, essentiality, and clinical relevance, this review is structured as follows. Section 2 systematically examines the physiological roles of each metal ion, organized by their molecular functions (e.g., electron transport, catalysis, signaling, and epigenetic regulation). Section 3 provides a comprehensive overview of metal‐associated diseases, structured by deficiency and overload states for each metal, with particular emphasis on clinical manifestations, underlying mechanisms, and recent therapeutic advances. Section 4 analyzes the multifactorial risk factors for metal imbalance, including dietary, environmental, genetic, and demographic determinants. Section 5 outlines current diagnostic paradigms for metal‐related disorders, integrating biochemical testing, imaging modalities, and genetic analysis. Finally, Section 6 reviews established and emerging therapeutic strategies targeting metal ions, from chelation and hormone replacement to novel ferroptosis and cuproptosis modulators. By synthesizing knowledge across these interconnected domains, this paper is dedicated to offering a valuable resource for researchers and clinicians working at the interface of metallobiology and diseases.

## 2 Physiological Roles of Metal Ions in Human Health

The physiological roles of metal ions are as diverse as their chemical properties, reflecting the distinct evolutionary paths by which they have been incorporated into human biology. This section delineates the functions of each of the four exemplary metals, that is, Fe, Cu, Zn, and Ca (Figure 1 and Table 1), with the goal of establishing the mechanistic foundation for understanding how metal dysregulation leads to the pathological conditions discussed in Section 3.

**FIGURE 1:**
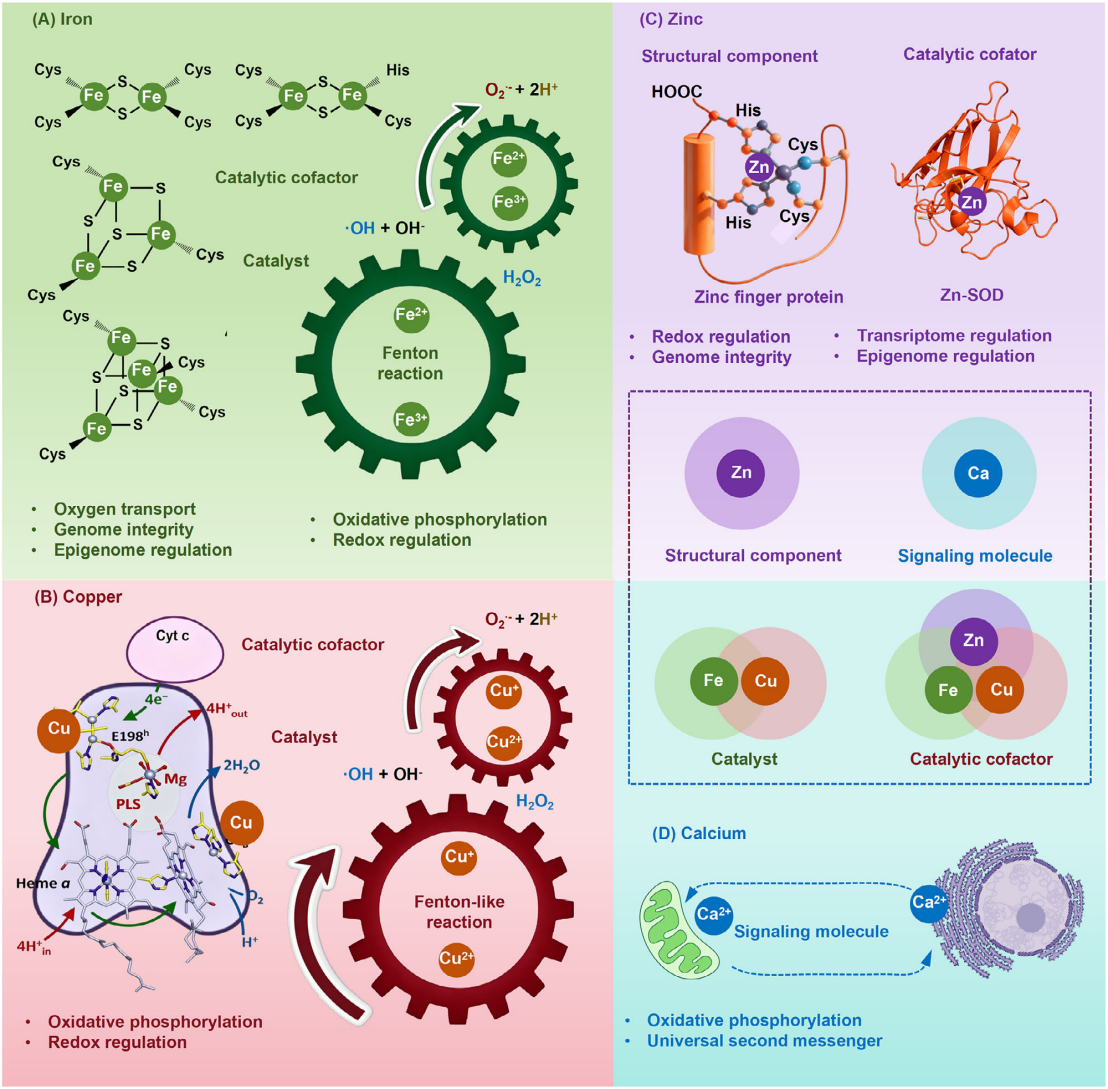
Physiological roles of iron and other metal ions in human health. Schematic overview of the major physiological functions of Fe, Cu, Zn, and Ca, organized by molecular mechanism. (A) Fe (redox‐active). Cycles between Fe^2+^ and Fe^3+^ to serve as a Fenton catalyst and a catalytic cofactor for heme‐containing (e.g., cytochrome *c*) and nonheme Fe enzymes (e.g., Fe–S cluster proteins). Fe is essential for redox regulation, OXPHOS, genome integrity, oxygen transport, and epigenetic regulation, among other roles. (B) Cu (redox‐active). Cycles between Cu^+^ and Cu^2+^ to function as a Fenton‐like catalyst and a catalytic cofactor for cuproenzymes such as SOD1 and cytochrome *c* oxidase (Complex IV). Cellular Cu homeostasis is essential for many events including, for example, redox regulation and OXPHOS. (C) Zn (redox‐inert). Serves as a structural component of, for example, Zn finger proteins, and as a catalytic cofactor for >300 enzymes. Zn is critical for, for example, transcriptional regulation, genome integrity, redox regulation, and epigenic control. (D) Ca (redox‐inert). Functions as a universal second messenger. Mitochondrial Ca uptake via the MCU complex activates TCA cycle dehydrogenases, coupling energy demand to ATP production. Ca also mediates muscle contraction, and neuronal function as a universal second messenger. While both redox‐active and redox‐inert metals serve as enzyme cofactors, redox‐active metals are unique in their role as catalysts, whereas redox‐inert metals play critical roles in maintaining structure and mediating signaling. ATP, adenosine triphosphate; Ca, calcium; Cu, copper; Fe, iron; MCU, mitochondrial calcium uniporter; OXPHOS, oxidative phosphorylation; SOD1, superoxide dismutase 1; TCA, tricarboxylic acid; Zn, zinc.

**TABLE 1:** Summary of ions and other exemplified metal ions: daily requirements, major sources, physiological function, pathological association.

Metal ion	Physiological functions	Pathological associations (deficiency)	Pathological associations (overload)	Daily requirement (adults)	Major dietary sources
Fe	Electron transport (ETC: cytochromes, Fe–S clusters) Oxygen transport (hemoglobin, myoglobin) Catalytic cofactor (cytochrome P450, catalase, myeloperoxidase, TPO, RNR, PHDs, lipoxygenases) Fe–S cluster biology (aconitase, IRP1/IRP2) Redox regulation (Fenton chemistry, GPX4 axis) Hormone synthesis (steroidogenesis, thyroid hormones) Epigenetic regulation (TET, JmjC demethylases) Data sources: [3, 4]	Fe deficiency anemia (microcytic, hypochromic) Fatigue, weakness, pallor Restless legs syndrome Impaired cognitive development in children Pregnancy complications (preterm birth, low birth weight) Glossitis, angular cheilitis, koilonychia, pica Data sources: [14]	Hereditary hemochromatosis (*HFE*, *HJV*, *HAMP*, *TFR2*, *SLC40A1* mutations) Transfusion‐dependent Fe overload (thalassemia, SCD) Ferroptosis Liver fibrosis, cirrhosis, HCC Cardiomyopathy, arrhythmias, heart failure Hypogonadotropic hypogonadism, diabetes mellitus, hypothyroidism Data sources: [14]	Men: 8 mg/day Women: 18 mg/day Pregnancy: 27 mg/day Data sources: [14, 33]	Red meat, liver, poultry, fish, shellfish (clams, oysters), legumes, spinach, fortified cereals Data sources: [14]
Cu	Catalytic cofactor in cuproenzymes (SOD1, COX, ceruloplasmin, LOX) Mitochondrial respiration (cytochrome *c* oxidase) Antioxidant defense (SOD1, ceruloplasmin) Fe metabolism (ceruloplasmin ferroxidase, hephaestin) Connective tissue formation (LOX cross‐linking) Neurotransmitter synthesis (dopamine β‐hydroxylase, PAM) Synaptic modulation (NMDA, GABA receptors) Data sources: [8]	Cu deficiency anemia (refractory to Fe) Neutropenia, pancytopenia Cu deficiency myelopathy (sensory ataxia, spasticity) Peripheral neuropathy Menkes disease (ATP7A mutations): neurological degeneration, kinky hair, connective tissue abnormalities Data sources: [18]	Wilson's disease (*ATP7B* mutations): hepatic cirrhosis, neurological symptoms (dystonia, tremor, dysarthria, ataxia), psychiatric disturbances, Kayser–Fleischer rings Cuproptosis Alzheimer's disease (amyloid‐β aggregation) Parkinson's disease (α‐synuclein aggregation) Tumor angiogenesis and metastasis Data sources: [18]	0.9 mg/day Data sources: [33]	Shellfish (oysters, crab), organ meats (liver), nuts (cashews, almonds), seeds, legumes, whole grains, dark chocolate Data sources: [18]
Zn	Structural component (Zn finger proteins: C2H2, RING, PHD, LIM; nuclear receptors; p53) Catalytic cofactor (>300 enzymes: alkaline phosphatase, carbonic anhydrase, alcohol dehydrogenase, MMPs) Epigenetic regulation (DNMT1, HDACs, sirtuins) Signaling (GPR39, NMDA receptor modulation) Proteostasis and autophagy (V‐ATPase, metallothioneins) Redox regulation (SOD1, NADPH oxidase inhibition) Hormone synthesis (insulin hexamerization, thyroid hormone regulation) Data sources: [9]	Growth retardation, delayed sexual maturation Alopecia, diarrhea, dermatitis (classic triad of acrodermatitis enteropathica) Immune dysfunction, increased infections Hypogonadism, night blindness Acrodermatitis enteropathica (SLC39A4 mutations) Transient neonatal Zn deficiency (SLC30A2 mutations) PAMI syndrome (functional deficiency) Data sources: [20]	Zn toxicity (acquired only; no inherited forms) Acute: nausea, vomiting, diarrhea (intake >200 mg/day) Chronic: neuropathy, anemia, fatigue, spasticity (intake 50–150 mg/day) ZICD: neutropenia, leukopenia, anemia, irreversible myelopathy Metal fume fever (occupational inhalation) Data sources: [20]	Men: 11 mg/day Women: 8 mg/day Pregnancy: 11–12 mg/day Data sources: [20, 33]	Oysters (richest source), red meat, poultry, crab, lobster, beans, nuts, dairy products, whole grains, fortified cereals Data sources: [20]
Ca	Universal second messenger Mitochondrial bioenergetics (MCU‐mediated Ca^2+^ uptake activates TCA cycle dehydrogenases) Ca^2+^/calmodulin signaling (CaMKs, calcineurin, NFAT, CREB) Excitation–contraction coupling (CICR via RyR, SERCA) Neurotransmitter release (voltage‐gated Ca^2+^ channels) Synaptic plasticity (LTP/LTD via NMDA receptors) Structural component of bone and teeth Data sources: [10]	Hypocalcemia: paresthesia, muscle cramps, tetany Chvostek's sign, Trousseau's sign Seizures, prolonged QT interval, arrhythmias Chronic: cataracts, dental abnormalities, dry skin, coarse hair Osteoporosis, rickets (with vitamin D deficiency) Hypoparathyroidism (postsurgical, autoimmune, genetic) Data sources: [23]	Hypercalcemia: fatigue, constipation, polyuria, polydipsia Nephrolithiasis, acute kidney injury Neuropsychiatric disturbances (confusion, depression) Cardiac arrhythmias Primary hyperparathyroidism (MEN1, MEN2A, MEN4, FHH) Malignancy‐associated hypercalcemia (PTHrP) CPPD (pseudogout): chondrocalcinosis, acute arthritis Data sources: [23]	Adults 19–50 yrs: 1000 mg/day Women >50 yrs: 1200 mg/day Men >70 yrs: 1200 mg/day Data sources: [32]	Dairy products (milk, yogurt, cheese), leafy greens (kale, broccoli), fortified plant milks, Ca‐set tofu, sardines, salmon (with bones), almonds, sesame seeds Data sources: [32]

Abbreviations: ACSL4, acyl‐CoA synthetase long‐chain family member 4; CaMK, Ca/calmodulin‐dependent protein kinase; CASR, Ca‐sensing receptor; CICR, Ca‐induced Ca release; CPPD, Ca pyrophosphate deposition disease; CREB, cAMP response element‐binding protein; DNMT1, DNA methyltransferase 1; DTPA, diethylenetriamine pentaacetate; ETC, electron transport chain; FDX1, ferredoxin 1; FHH, familial hypocalciuric hypercalcemia; GPR39, G protein‐coupled receptor 39; GPX4, glutathione peroxidase 4; HCC, hepatocellular carcinoma; HDAC, histone deacetylase; HFE, homeostatic Fe regulator; HJV, hemojuvelin; IP3R, inositol trisphosphate receptor; IRP, Fe regulatory protein; JmjC, Jumonji C; LOX, lysyl oxidase; LTP/LTD, long‐term potentiation/long‐term depression; MCU, mitochondrial Ca uniporter; MEN, multiple endocrine neoplasia; MICU, mitochondrial Ca uptake protein; MMP, matrix metalloproteinase; mPTP, mitochondrial permeability transition pore; NCLX, Na+/Ca2+/Li+ exchanger; NCX, Na^+^/Ca^2^^+^ exchanger; NFAT, nuclear factor of activated T‐cells; NMDA, N‐methyl‐d‐aspartate; PAMI, PSTPIP1‐associated myeloid‐related proteinemia inflammatory syndrome; PHD, prolyl hydroxylase (enzyme) or plant homeodomain (Zn finger); PTH, parathyroid hormone; PTHrP, parathyroid hormone‐related peptide; RNR, ribonucleotide reductase; RyR, ryanodine receptor; SCD, sickle cell disease; SERCA, sarco/endoplasmic reticulum Ca‐ATPase; SOD1, superoxide dismutase 1; TCA, tricarboxylic acid; TET, ten‐eleven translocation; TPO, thyroid peroxidase; TSAT, transferrin saturation; V‐ATPase, vacuolar‐type ATPase; ZICD, Zn‐induced Cu deficiency.

### 2.1 Physiological Roles of Fe

Fe is an indispensable cofactor across a broad spectrum of fundamental molecular processes, a necessity driven by its unique ability to cycle between ferrous (Fe^2+^) and ferric (Fe^3+^) states. This redox versatility enables Fe to serve as both a catalyst for Fenton reaction and a catalytic cofactor for approximately one‐third of all enzymes, thereby embedding it into the core machinery of cellular metabolism and gene regulation [3, 4, 34] (Figure 1).

#### 2.1.1 Fe as Fenton Catalyst

Free or labile Fe can participate in Fenton chemistry as a catalytic agent. In this context, Fe^2+^ catalyzes the conversion of hydrogen peroxide (H_2_O_2_) to the highly reactive hydroxyl radical (•OH), a species capable of attacking all classes of biomolecules, including lipids, proteins, and DNA. This property is physiologically exploited by phagocytes (via myeloperoxidase) for microbial killing; however, when uncontrolled, it drives lipid peroxidation and ferroptosis, an Fe‐dependent form of regulated cell death, as well as broader tissue damage  [35].

#### 2.1.2 Fe as Catalytic Cofactor

Fe, by virtue of its ability to cycle between Fe^2+^ and Fe^3+^, serves not only as a Fenton catalyst but also as a tightly bound cofactor for numerous enzymes, broadly classified into heme‐containing and nonheme Fe enzymes. In redox regulation, heme‐dependent enzymes such as cytochrome P450, catalase, and myeloperoxidase mediate oxidative metabolism, antioxidant defense, and microbial killing, respectively; while nonheme Fe enzymes including prolyl hydroxylases and lipoxygenases (ALOX family) regulate hypoxia responses via HIF‐1α stability and generate bioactive lipid mediators from polyunsaturated fatty acids. The glutathione–GPX4 axis, though selenium‐dependent, counteracts lipid peroxidation and ferroptosis, placing Fe at a critical crossroads where its imbalance dictates the threshold for ferroptotic cell death [24, 25].

In oxidative phosphorylation (OXPHOS), Fe is indispensable for mitochondrial electron transport. Heme groups in cytochromes (*a*, *a*_3_, *b*, *c*, *c*_1_) and iron–sulfur (Fe–S) clusters in Complexes I–III (and aconitase in the tricarboxylic acid [TCA] cycle) facilitate sequential electron transfer from NADH/FADH_2_ to oxygen (O_2_), driving proton gradient formation and ATP synthesis. Inadequate Fe availability impairs this process, leading to reduced ATP production and increased ROS leakage [36, 37].

For genome integrity, nonheme Fe–S clusters are essential structural components of DNA helicases (XPD, FANCJ, RTEL1) and polymerases (Polδ, PrimPol) [3, 4, 34, 38, 39], while the di‐Fe center of ribonucleotide reductase catalyzes the rate‐limiting reduction of ribonucleotides to deoxyribonucleotides. Thus, nonheme Fe directly supports DNA replication and repair, whereas heme Fe indirectly influences genome stability through redox balance [40, 41, 42].

In epigenetic regulation, nonheme Fe^2+^/α‐ketoglutarate‐dependent dioxygenases, the TET family for DNA demethylation and JmjC domain‐containing histone demethylases, catalyze oxidative removal of methyl marks, dynamically shaping chromatin structure and gene expression [43]. Fe deficiency suppresses these enzymes, causing lasting epigenetic changes that may affect differentiation and disease susceptibility even after repletion [44, 45].

For O_2_ transport, heme Fe in hemoglobin (tetrameric, with cooperative O_2_ binding) enables efficient O_2_ carriage from lungs to tissues, while myoglobin (monomeric, higher O_2_ affinity) serves as an intracellular O_2_ reservoir in cardiac and skeletal muscle [3, 4, 34].

Beyond these roles, Fe is critical for hormone synthesis. Heme‐dependent cytochrome P450 enzymes (CYP11A1, CYP17A1, CYP19A1) mediate steroidogenesis in adrenal and gonadal tissues [46], and thyroid peroxidase catalyzes iodination and coupling reactions for thyroid hormone synthesis [47, 48]. Nonheme Fe–S proteins in pancreatic β‐cells influence glucose sensing and insulin secretion, establishing a bidirectional relationship between Fe and glucose metabolism [49, 50, 51, 52].

In summary, the unique redox versatility of Fe underpins its dual role as both a catalyst and a cofactor, rendering it indispensable for redox regulation, OXPHOS, genome integrity, epigenetic control, O_2_ carriage, and hormone production. Yet this same chemistry also confers the potential for oxidative damage when homeostasis is disturbed.

### 2.2 Physiological Roles of Cu

Cu is an essential trace element that plays a dual role in cellular physiology, driven by its unique ability to cycle between cuprous (Cu^+^) and cupric (Cu^2+^) states. This redox versatility enables Cu to function both as a nonenzymatic Fenton‐like catalyst and as a catalytic cofactor for a diverse array of cuproenzymes, thereby making it indispensable for mitochondrial respiration, antioxidant defense, neurotransmitter synthesis, and connective tissue formation [8] (Figure 1 and Table 1).

#### 2.2.1 Cu as Fenton‐Like Catalyst

As a redox‐active transition metal, Cu can participate in Fenton‐like reactions, generating highly reactive •OH from hydrogen peroxide. These radicals attack all classes of biomolecules, including lipids, proteins, and DNA. This pro‐oxidant activity, when uncontrolled, contributes to oxidative stress and has been implicated in the pathogenesis of various diseases. Unlike Fe, Cu is more potent in catalyzing radical formation from certain substrates, but its free concentration is kept extremely low by tight homeostatic mechanisms. The Cu‐dependent antioxidant system is particularly important in tissues with high oxidative demand, such as the brain, where Cu imbalance has been linked to neurodegenerative disorders including Alzheimer's and Parkinson's diseases [7, 8].

#### 2.2.2 Cu as Catalytic Cofactor

Cu cycles between Cu^+^ and Cu^2+^, functioning as a catalytic cofactor for a diverse array of Cu‐dependent enzymes (cuproenzymes) that underpin multiple physiological processes.

In redox regulation, Cu/Zn‐superoxide dismutase (SOD1) catalyzes superoxide dismutation, providing first‐line antioxidant defense [53]. Ceruloplasmin and hephaestin, both multi‐Cu oxidases, exhibit ferroxidase activity that converts Fe^2+^ to Fe^3+^, thereby limiting Fenton chemistry and enabling Fe loading onto transferrin. Thus, Cu indirectly governs Fe homeostasis, and its deficiency can cause anemia refractory to Fe supplementation alone [8, 11].

In mitochondrial respiration, cytochrome *c* oxidase (COX), the terminal electron transport chain (ETC) complex, contains Cu_A_ and Cu_B_ centers that facilitate electron transfer to O_2_ and proton translocation for ATP synthesis. Thus, inadequate Cu impairs COX activity, reducing ATP production and increasing ROS generation [8, 11].

In the central nervous system, Cu homeostasis is stringently maintained through coordinated action of importers (CTR1), chaperones (e.g., COX17), and exporters (ATP7A and ATP7B), reflecting the dual imperative of ensuring Cu availability while preventing its toxicity. Disruption of this delicate balance has been implicated in Alzheimer's and Parkinson's diseases [7]. Functionally, Cu‐dependent dopamine β‐hydroxylase and peptidylglycine α‐amidating monooxygenase are essential for catecholamine and neuropeptide synthesis, and Cu itself modulates synaptic transmission through NMDA and GABA receptors. Accordingly, impairment of these Cu‐dependent processes contributes to the neurological manifestations of Menkes and Wilson's diseases [7, 8, 54].

For connective tissue integrity, lysyl oxidase catalyzes oxidative deamination of lysine/hydroxylysine residues in collagen and elastin, initiating cross‐linking essential for tensile strength and elasticity. Cu deficiency thus results in vascular fragility, skin laxity, and skeletal abnormalities [8, 11].

In summary, the redox versatility of Cu has made it indispensable for antioxidant defense, Fe metabolism, mitochondrial bioenergetics, neurotransmission, and connective tissue formation, while its dysregulation contributes to a broad spectrum of human diseases.

### 2.3 Physiological Roles of Zn

Zn is an essential trace element that plays a dual role in cellular physiology, distinguished by its unique property as a redox‐inert transition metal. Unlike Fe and Cu, Zn does not cycle between oxidation states under physiological conditions and therefore cannot participate in Fenton‐type redox reactions. Instead, Zn serves two primary functions: as a structural component that stabilizes the folding of thousands of proteins, and as a catalytic cofactor for more than 300 enzymes across all six enzyme classes [3, 4, 10, 12, 34, 55, 56]. This redox stability, combined with its versatile coordination chemistry, renders Zn indispensable for transcriptional regulation, epigenetic control, antioxidant defense, neurological function, hormone signaling, and proteostasis [8, 9, 10, 12] (Figure 1 and Table 1).

#### 2.3.1 Zn as a Structural Component

As a structural component, Zn coordinates within protein folds to stabilize tertiary and quaternary structures, supporting molecular recognition, DNA binding, and protein–protein interactions without directly participating in catalysis. Zn‐finger proteins, one of the largest and most diverse classes of Zn‐binding proteins, are central to transcriptional regulation, ubiquitin conjugation, chromatin remodeling, and cytoskeletal organization [55, 56]. Zn is also essential for the tumor suppressor p53 and its homologues, where a single Zn in the DNA‐binding domain preserves proper folding for dimer/tetramer formation and accurate recognition of response elements [9, 57, 58]. All nuclear receptors contain conserved DNA‐binding domains composed of two Zn‐finger motifs that recognize palindromic hormone‐response elements, including receptors for sex steroids, adrenal hormones, vitamin D, thyroid hormone, and PPARs [9, 59, 60]. In the pancreas, Zn is required for insulin hexamerization and stability; pancreatic β‐cells possess the highest Zn content, and Zn transporters such as ZnT8 and ZIP7 modulate insulin signaling, glucose uptake, and glycemic control [61]. In the thyroid, Zn regulates the synthesis of thyrotropin (TSH) and TSH‐releasing hormone (TRH) by modulating their structure and the transcription factors essential for thyroid hormone production; Zn deficiency reduces TSH, throxine, and triiodothyronine levels, while supplementation raises triiodothyronine [47, 48, 62]. Thus, through its structural roles, Zn underpins transcriptional regulation, genomic stability, hormonal signaling, and metabolic homeostasis.

#### 2.3.2 Zn as a Catalytic Cofactor

As a catalytic cofactor, Zn serves over 300 enzymes across all six classes, typically acting as a Lewis acid that polarizes substrates or stabilizes transition states without undergoing redox changes, distinguishing it from Fe and Cu.

In redox regulation, Zn exerts both indirect and direct antioxidant effects. Indirectly, it is a structural component of SOD1 (where Cu provides catalytic activity) and induces metallothionein expression, which buffers Zn and scavenges free radicals. Directly, Zn competes with redox‐active metals for binding sites, thereby preventing Fenton chemistry, and inhibits NADPH oxidase to reduce ROS production [8, 20, 63]. These actions are particularly important in the retina, where Zn supplementation slows age‐related macular degeneration [8, 63, 64, 65].

In epigenetic regulation, Zn is indispensable for DNA methyltransferase 1, histone methyltransferases, demethylases (e.g., KDM2A), histone acetyltransferases, deacetylases, and NAD^+^‐dependent sirtuins, thereby preserving the epigenetic landscape and cellular identity [9, 43, 44, 45, 66, 67, 68].

In the nervous system, Zn modulates glutamatergic (including NMDA) receptors to regulate synaptic plasticity and neuronal excitability, while exchangeable Zn influences kinase/phosphatase networks and activates the Zn‐sensing receptor GPR39, which enhances sperm motility and acrosomal exocytosis, suggesting a role in male fertility [9, 66, 69, 70, 71]. Additional Zn‐dependent enzymes perform essential functions: alkaline phosphatase (ALP) supports bone mineralization and serves as a functional Zn marker; carbonic anhydrase regulates pH and CO_2_ transport; alcohol dehydrogenase participates in alcohol metabolism; and matrix metalloproteinases mediate tissue remodeling and wound healing [3, 4, 10, 12, 34, 72].

In summary, the dual roles of Zn as a structural scaffold and catalytic cofactor collectively support transcriptional regulation, genomic stability, hormone signaling, redox balance, epigenetic control, neurological function, and reproductive health.

### 2.4 Physiological Roles of Ca

Ca is a ubiquitous second messenger that plays a crucial role in regulating mitochondrial workload and overall cellular homeostasis [9, 10]. Unlike transition metals such as Fe and Cu, free Ca is in the form of Ca^2+^ that is a redox‐inert metal and does not participate in redox reactions but instead functions as a signaling ion, with its concentration tightly controlled across cellular compartments. The unique ability of Ca to couple cellular energy demand with mitochondrial ATP production makes it indispensable for cell survival and function (Figure 1 and Table 1).

The physiological roles of Ca center on its function as a signaling ion, mediated by tightly regulated flux across mitochondrial and cellular membranes. Mitochondria possess outer and inner membranes; the inner membrane's selective permeability maintains the electrochemical gradient essential for ATP production [22, 73, 74, 75, 76]. Endoplasmic reticulum (ER)–mitochondria contacts (10–30 nm) creat high‐Ca^2+^ microdomains that facilitate efficient uptake [77] via the mitochondrial Ca uniporte (MCU) complex, whose gating by mitochondrial Ca uptake proteins (MICUs) prevented overload [78]. Once in the matrix, Ca^2+^ activated TCA dehydrogenases (pyruvate dehydrogenase, isocitrate dehydrogenase, α‐ketoglutarate dehydrogenase), increasing NADH/FADH_2_ and ATP synthesis, coupling energy demand to mitochondrial output [10, 23, 79]. Efflux via mitochondrial Na^+^/Ca^2+^ exchanger and Na^+^/Ca^2+^ exchanger (NCX) prevented prolonged overload and mitochondrial permeability transition pore (mPTP) opening [80, 81, 82, 83]. As a universal second messenger, Ca^2+^ released by IP_3_ binds calmodulin, which activated Ca/calmodulin‐dependent protein kinases (CaMKs), calcineurin, nuclear factor of activated T‐cells (NFAT), and cyclic adenosine monophosphate (cAMP) response element‐binding protein (CREB) to regulate gene expression, immunity, contraction, and secretion [9, 10]. In muscle, L‐type Ca^2+^ channels triggered ryanodine receptor (RyR)‐mediated Ca^2+^‐induced Ca^2+^ release, enabling contraction via troponin C, with sarco/ER Ca‐ATPase mediating relaxation [10, 80]. In neurons, presynaptic Ca^2+^ influx triggered vesicle release, while postsynaptic NMDA receptor activation of CaMKII underpins long‐term potentiation/long‐term depression [9, 10]. Thus, unlike redox‐active Fe/Cu and structural Zn, Ca acts predominantly as a signaling Fe that couples energy demand with ATP production and governs muscle and neural function.

### 2.5 Summary

Collectively, Fe (redox catalyst and cofactor for ∼1/3 of enzymes, supporting OXPHOS, genome integrity, epigenetics, and oxygen transport), Cu (redox cofactor for cuproenzymes, supporting antioxidant defense and respiration), Zn (redox‐inert structural and catalytic scaffold for hundreds of enzymes, supporting transcription, genomic stability, and epigenetics), and Ca (redox‐inert signaling ion, coupling energy to ATP and regulating muscle/neural activity) illustrate how metal chemistry dictates biological roles (Table 1 and Figure 1), with interconnections such as Cu facilitating Fe metabolism, Zn modulating Ca signaling, and Ca regulating Fe‐dependent respiration. This integrated understanding underpins the pathological consequences of metal imbalance discussed in the next section.

## 3 Pathological Associations of Metal Ions in Human Diseases

The physiological roles of metal ions detailed in Section 2 underscore their indispensability for normal cellular function. However, when the finely tuned homeostatic mechanisms that govern metal absorption, distribution, storage, and excretion are disrupted, the same chemical properties that make these ions biologically advantageous can become pathogenic. Redox‐active metals such as Fe and Cu, when accumulated in excess, catalyze the generation of ROS via Fenton chemistry, driving oxidative tissue damage and ferroptosis or cuproptosis. Redox‐inert metals such as Zn and Ca, when deficient or mislocalized, compromise structural integrity, signaling cascades, and metabolic regulation, contributing to a broad spectrum of human diseases. This section systematically examines the pathological associations of metal ions, structured by metal type, Fe (Section 3.1), Cu (Section 3.2), Zn (Section 3.3), and Ca (Section 3.4), each organized into deficiency and overload states where applicable, with emphasis on clinical manifestations, underlying mechanisms, and epidemiological evidence.

### 3.1 Fe‐Associated Diseases

Fe is a double‐edged sword in human health, as both deficiency and overload can lead to significant morbidity (Figure 3). The present section consolidates current understanding of Fe‐related diseases, categorized into deficiency and overload entities, and examines their clinical phenotypes, pathogenetic pathways, and available therapeutic modalities.

#### 3.1.1 Fe Deficiency

Fe deficiency is the most prevalent micronutrient deficiency globally, affecting approximately one in four individuals worldwide. The highest burden falls on children, women of reproductive age, and populations in low‐ and middle‐income countries [84, 85]. Fe deficiency spans a continuous spectrum, from depletion of body Fe stores (normal hemoglobin, low ferritin) to overt Fe deficiency anemia, characterized by reduced hemoglobin levels resulting from inadequate Fe availability [14].

The clinical manifestations of Fe deficiency extend beyond anemia, encompassing fatigue, cognitive impairment, pregnancy complications, and neurodevelopmental delay in children [14] (Table 1). In pregnancy, Fe deficiency poses a particularly critical concern: up to 84% of women in the third trimester are affected, with associated risks including preterm birth, low birth weight, and increased maternal morbidity [14, 84, 85].

Diagnosis is typically based on serum ferritin (<30 ng/mL in individuals without inflammation) or transferrin saturation (<20%). Oral Fe (e.g., ferrous sulfate 325 mg daily or on alternate days) remains the mainstay of first‐line therapy, whereas intravenous Fe is increasingly reserved for patients with intolerance, malabsorption, chronic inflammatory conditions, or ongoing blood loss  [14, 84, 85].

#### 3.1.2 Fe Overload

Fe overload disorders encompass a heterogeneous group of conditions characterized by progressive Fe accumulation in parenchymal organs, culminating in multisystem damage [15]. Tissue injury is primarily driven by ferroptosis, a form of Fe‐dependent cell death triggered by ROS generated via the Fenton reaction [35, 86]. Ferroptosis is driven by the convergence of Fe overload, enhanced lipid peroxidation (notably via ACSL4‐dependent phospholipid remodeling), and impaired antioxidant defenses, primarily mediated by the System Xc^−^/GPX4 axis [29]. Fe‐dependent enzymes, such as arachidonate lipoxygenase, further amplify lipid peroxidation, producing reactive aldehydes that impair protein function and provoke inflammation [29]. Collectively, these processes culminate in irreversible cellular injury, particularly in Fe‐rich organs such as the liver, heart, and endocrine glands [87, 88].

HH is the most common genetic Fe overload disorder, affecting an estimated 1/200 to 1/300 individuals of European descent [13]. HH is caused by mutations in Fe‐regulatory genes, most commonly *HFE* (Type 1), as well as *HJV*, *HAMP*, *TFR2*, and *SLC40A1*. These mutations lead to inappropriately low hepcidin levels, resulting in uncontrolled intestinal Fe absorption. Progressive Fe accumulation drives multiorgan damage, particularly in the liver, heart, and endocrine glands, through ferroptosis [89] (Table 1).

Transfusion‐dependent Fe overload occurs in patients with congenital anemias requiring chronic red blood cell transfusions, including β‐thalassemia major, sickle cell disease, and myelodysplastic syndromes. Each unit of packed red blood cells contains approximately 200–250 mg of Fe, and the body lacks any physiological mechanism for excess Fe excretion. Without adequate chelation, progressive Fe deposition occurs in the liver, heart, and endocrine glands [15, 90]. For instance, hepatic Fe accumulation progressed from fibrosis to cirrhosis [91] and hepatocellular carcinoma [13, 92, 93], whereas cardiac Fe overload remains a leading cause of mortality in transfusion‐dependent patients, manifesting as cardiomyopathy, arrhythmias, and heart failure [94].

Diabetes as a complication of Fe overload warrants special attention. Epidemiological evidence demonstrates that higher systemic Fe levels are associated with an elevated risk of Type 2 diabetes, whereas Fe depletion reduces the risk [89]. Mechanistically, Fe‐mediated oxidative damage to pancreatic β‐cells via Fenton chemistry impairs insulin secretion. In patients with HH or thalassemia, diabetes prevalence is substantially increased, and nontransferrin‐bound Fe (NTBI) levels correlate with disease severity [89].

The reproductive system is particularly vulnerable to Fe toxicity. Large‐scale analyses have quantified the substantial burden of Fe‐induced reproductive dysfunction. Among 1201 men and 2134 women with Fe overload disorders, hypogonadism was reported in 47.0% of men; among those specifically evaluated for spermatogenesis, azoospermia was present in 17.6% and other sperm abnormalities in 37.5%. In women, primary amenorrhea was reported in 45.7%, secondary amenorrhea in 20.0%, and the weighted mean age at menarche was significantly delayed (14.4 ± 2.1 years). Across 3536 reviewed pregnancies, assisted reproductive technology was required in approximately 20%, miscarriage occurred in 11.2%, and cesarean section was performed in approximately 80%. Mean gestational age at delivery was 37.1 ± 3.1 weeks, and mean birth weight was 2.64 ± 0.68 kg [15, 16, 90, 95, 96].

In males, Fe deposition predominantly affects pituitary gonadotrophs, leading to reduced gonadotropin secretion, impaired testosterone production, and spermatogenic failure. Erectile dysfunction affects approximately 50–70% of male patients with thalassemia major [15]. Mechanistically, ferroptosis has emerged as a key pathway underlying male reproductive damage. Early proteomic analyses documented altered expression of the antiferroptotic enzyme GPX4 in patients with spermatogenic failure [97]. Subsequent transcriptomic, ultrastructural, and biochemical analyses of testicular biopsies from men with idiopathic nonobstructive azoospermia demonstrated downregulation of ferroptosis‐related genes (including GPX4), Fe accumulation, increased lipid peroxidation, and mitochondrial morphological features consistent with ferroptotic cell death [24]. Furthermore, infertile men exhibit elevated ACSL4 levels, correlations between ferritin, isoprostanes, and impaired semen parameters, and localization of lipid peroxidation‐promoting enzymes in spermatozoa, collectively indicating activation of ferroptosis‐related pathways in human germ cells [25].

In females, Fe overload leads to delayed puberty, premature ovarian insufficiency, and menstrual disturbances. Fe accumulation in ovarian granulosa cells induces ferroptosis, contributing to reduced ovarian reserve and infertility [16, 90]. Beyond direct gonadal damage, systemic Fe‐related endocrine and metabolic disturbances, including hypothyroidism, growth hormone deficiency, diabetes mellitus, and cardiovascular disease, further compound reproductive impairments [95, 96].

### 3.2 Cu‐Associated Diseases

Dysregulated Cu homeostasis, whether manifested as deficiency or excess, has been implicated in a broad spectrum of human diseases [17]. Because redox‐active transition metals such as Cu may participate in electron transfer reactions, their homeostasis must be carefully controlled [8, 17] (Figure 2). Cu‐associated diseases can be broadly categorized into those resulting from insufficient Cu availability (Cu deficiency) and those arising from excessive Cu accumulation (Cu overload).

**FIGURE 2:**
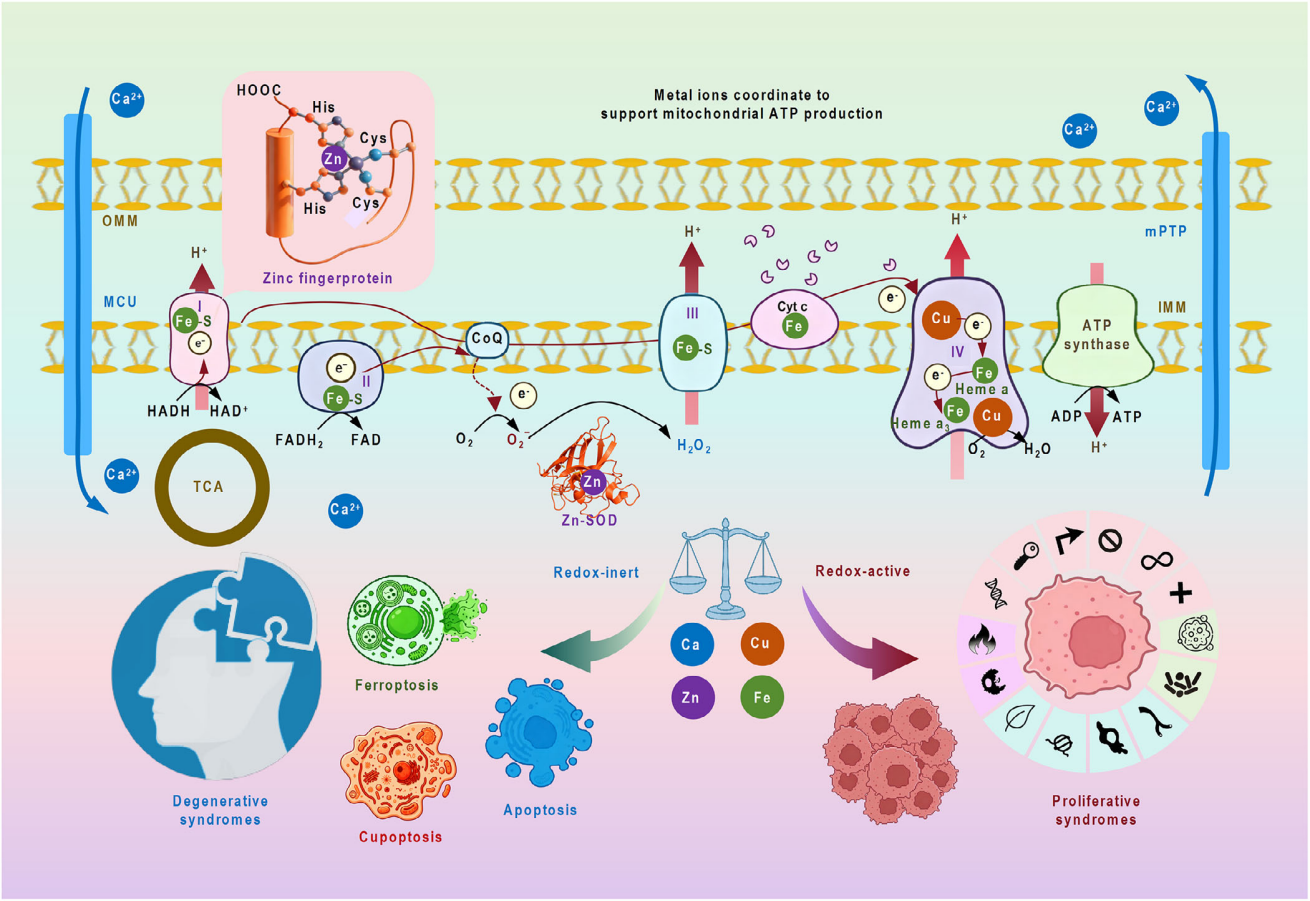
Coordination of redox‐active and redox‐inert metal ions in human health and disease. Oxidative phosphorylation exemplifies how these two classes of metal ions cooperatively support mitochondrial ATP production. Redox‐active Fe, embedded within Fe–S clusters in Complexes I–III, facilitates electron transfer along the respiratory chain. Redox‐active Cu, present as CuA and CuB centers in Complex IV (cytochrome *c* oxidase), enables the final electron transfer to molecular oxygen. Redox‐inert Zn maintains the structural integrity of mitochondrial proteins such as NDUFS6, which supports Complex I assembly and stability, and regulates the catalytic function of antioxidant SOD1. Redox‐inert Ca acts as a signaling molecule; mitochondrial Ca uptake via the MCU complex activates TCA cycle dehydrogenases, coupling energy demand to ATP production. Thus, OXPHOS uniquely integrates redox‐active metals (Fe, Cu) as electron carriers, a redox‐inert structural metal (Zn) as a regulator, and a redox‐inert signaling metal (Ca) as a metabolic pacemaker. Imbalance between redox‐active and redox‐inert metal ions leads to dysregulated energy production and ROS generation, which can either drive cells toward uncontrolled proliferation, resulting in proliferative syndromes such as cancer, or trigger undesirable cell death (e.g., ferroptosis, cuproptosis, apoptosis), contributing to degenerative disorders such as Alzheimer's disease. ATP, adenosine triphosphate; Ca, calcium; Cu, copper; Fe, iron; MCU, mitochondrial calcium uniporter; NDUFS6, NADH:ubiquinone oxidoreductase subunit S6; OXPHOS, oxidative phosphorylation; ROS, reactive oxygen species; SOD1, superoxide dismutase 1; TCA, tricarboxylic acid; Zn, zinc.

#### 3.2.1 Cu Deficiency

Cu deficiency, though relatively rare, can give rise to significant hematological and neurological abnormalities. Etiologically, acquired Cu deficiency syndromes may arise from malabsorption following gastric surgery (e.g., Roux‐en‐Y gastric bypass, gastrectomy), excessive Zn ingestion (which upregulates enterocyte metallothionein, thereby sequestering Cu and impeding its absorption), or rare genetic disorders [98, 99]. Additional contributing factors include malnutrition, prolonged total parenteral nutrition without adequate Cu supplementation, enteropathies with protein‐losing enteropathy, and chronic kidney disease treated with high‐dose Zn [98, 99].

Cu deficiency produces haematological (anemia, neutropenia) and neurological (myeloneuropathy) sequelae, reflecting its essential roles in hematopoiesis and myelin maintenance [98, 99, 100] (Table 1). The hematological features stem from Cu's requirement as a cofactor for enzymes involved in Fe metabolism (e.g., ceruloplasmin, hephaestin) and erythroid differentiation. The neurological features, Cu deficiency‐associated myeloneuropathy, closely resemble subacute combined degeneration seen in vitamin B_12_ deficiency, and may present with paresthesia, proprioceptive deficits, and spasticity.

Diagnosis is confirmed by low serum Cu and ceruloplasmin levels, with or without concomitant Zn elevation (see Section 5.2 for detailed diagnostic workup). Treatment consists of Cu repletion (oral or intravenous Cu supplementation). While hematological abnormalities often respond favorably to repletion, neurological deficits frequently show only partial or no improvement, underscoring the importance of early recognition and intervention [98, 99].

#### 3.2.2 Cu Overload

Cu excess is associated with a range of clinically significant conditions, from inherited disorders to acquired states of Cu accumulation. The most well‐characterized Cu‐associated disorder is Wilson's disease, an autosomal recessive disorder caused by mutations in the *ATP7B *gene, which encodes a Cu‐transporting P‐type ATPase required for biliary Cu excretion. Impaired ATP7B function leads to pathological Cu accumulation, primarily in the liver and brain. Wilson's disease manifests with hepatic, neurological (movement disorders), and psychiatric features; Kayser–Fleischer rings are pathognomonic when present [18, 101] (Table 1).

Diagnosis is based on a combination of clinical features, low serum ceruloplasmin, elevated 24‐h urinary Cu excretion, and confirmatory genetic testing. Treatment options includes Cu chelation therapy (D‐penicillamine, trientine) or Zn acetate, which blocks intestinal Cu absorption [18, 101].

Beyond Wilson's disease, Cu dysregulation has been implicated in a range of other pathological conditions involving disrupted redox‐active metal homeostasis, including neurological disorders (Alzheimer's, Parkinson's, and Huntington's diseases), psychiatric conditions, cardiovascular disease, cancer, and diabetes [8]. In Alzheimer's disease, Cu accumulation has been observed in senile plaques and may promote amyloid‐β aggregation and oxidative stress [8]. In Parkinson's disease, elevated Cu levels may contribute to α‐synuclein aggregation and dopaminergic neuron degeneration [8].

Elevated Cu concentrations have been reported in tumor tissues or serum of patients with various malignancies, including breast, ovarian, lung, gastric, thyroid, and prostate cancers [17, 28, 102]. Cu promotes tumor angiogenesis through its role as a cofactor for angiogenic factors such as vascular endothelial growth factor and basic fibroblast growth factor. Additionally, Cu‐dependent enzymes, including LOX and SOD1, facilitate extracellular matrix remodeling and protect cancer cells from oxidative stress, thereby promoting tumor progression and metastasis [17, 28, 102].

The discovery of cuproptosis, a Cu‐dependent, nonapoptotic form of regulated cell death, has provided a novel paradigm for understanding Cu‐mediated cytotoxicity [17, 28, 102]. Cuproptosis was first described in 2022 and is characterized by the direct binding of intracellular Cu to lipoylated components of the TCA cycle, triggering proteotoxic stress and aggregation of lipoylated proteins, rather than generalized ROS damage [26].

This form of cuproptosis is distinct from apoptosis, ferroptosis, necroptosis, and pyroptosis, and is critically dependent on mitochondrial respiration and the upstream regulator ferredoxin 1 (FDX1), which reduces Cu^2+^ to Cu^+^, the active form that binds lipoylated proteins [28]. Cells that rely on OXPHOS exhibit markedly greater sensitivity to Cu‐induced cuproptosis than those utilizing glycolysis, as the TCA cycle and lipoylated mitochondrial enzymes (including pyruvate dehydrogenase, α‐ketoglutarate dehydrogenase, branched‐chain ketoacid dehydrogenase, and glycine cleavage system protein H) are essential for this cell death pathway.

Cuproptosis has been implicated in the pathogenesis of various diseases, including genetic disorders, liver diseases, neurodegenerative disorders, and cancers [28]. The emerging discipline of “coprology” aims to systematically investigate the role of Cu in life activities and disease pathogenesis [17]. Further research is needed to fully elucidate the mechanisms linking Cu homeostasis and cuproptosis to human health and disease, and to develop targeted therapeutic strategies based on Cu metabolism, including Cu chelators, Cu ionophores, and modulators of cuproptosis for cancer therapy [28].

### 3.3 Zn‐Associated Diseases

Zn homeostasis is essential for numerous physiological processes, and its disruption, whether through insufficient intake, impaired absorption, excessive losses, or intracellular misdistribution, contributes to metabolic imbalance, accelerates aging‐associated decline, and participates in the etiology of both inherited and acquired pathologies [10, 12, 63]. Deficiency or mislocalization of Zn is implicated in a wide range of medical conditions, including cardiometabolic and renal disorders, immune dysfunction and chronic inflammation, neurodegeneration, neurodevelopmental disorders (including autism spectrum disorder and Kleefstra syndrome), endocrine disturbances, and age‐related decline [9, 12, 63] (Figure 2). Zn‐associated diseases can be broadly categorized into Zn deficiency states (acquired or inherited) and Zn toxicity, with the latter giving rise to a clinically important complication known as Zn‐induced Cu deficiency (ZICD).

#### 3.3.1 Zn Deficiency

Zn deficiency may be either acquired or inherited. Acquired Zn deficiency can result from insufficient dietary intake (as seen in anorexia nervosa), increased losses (chronic diarrhea or burns), increased physiological requirements (pregnancy and lactation), or malabsorption (Crohn's disease and coeliac disease) [20, 72, 103, 104, 105]. The clinical presentation, characterized by diarrhea, frequent infections, and dermatological lesions, typically emerges later in life, coinciding with the development of these predisposing conditions [19].

Inherited Zn deficiency is most commonly associated with mutations in the *SLC39A4 *gene on chromosome 8, which encodes the Zn transporter ZIP4 [70, 71]. The resulting pathological condition is acrodermatitis enteropathica, a rare autosomal recessive disorder with an incidence of approximately one in 500,000 births, affecting males and females equally [20, 72, 103, 104, 105, 106]. ZIP4 normally mediates the transport of Zn ions from the intestinal lumen into the enterocyte; loss‐of‐function mutations prevent this uptake, leading to insufficient Zn reaching the systemic circulation and distant tissues [107]. Affected infants typically become symptomatic early in life, particularly during the transition from breastfeeding to weaning [20, 72, 103, 104, 105, 108].

Zn deficiency presents with the classic triad of alopecia, diarrhea, and dermatitis [109], accompanied by growth failure and immune dysfunction [20, 72, 103, 104, 105] (Table 1). Without treatment, acrodermatitis enteropathica is fatal within the first few years of life [106].

Another genetic cause of Zn deficiency is transient neonatal Zn deficiency (TNZD), which arises from mutations in the maternal *SLC30A2* gene. This gene encodes ZnT2, a Zn transporter expressed in mammary glands [110]. Mutations reduce Zn secretion into breast milk, leading to severe Zn deficiency in exclusively breastfed infants despite their normal intestinal transport capacity [61].

Beyond acrodermatitis enteropathica and TNZD, mutations in other Zn transporters give rise to distinct clinical syndromes. *ZIP8* mutations result in cortical atrophy and consequent intellectual disability. Mutations in *SLC39A14* (encoding ZIP14) can lead to parkinsonism‐dystonia in children [111, 112], while* ZIP13 *mutations cause the spondylocheirodysplastic form of Ehlers–Danlos syndrome by impairing Zn export from the Golgi apparatus into the cytosol, thereby disrupting bone morphogenetic protein and transforming growth factor‐β signaling and compromising connective tissue development [55, 56]. Birk–Landau–Perez syndrome results from mutations in* SLC30A9* (ZnT9), manifesting as a cerebromelan syndrome with neurodevelopmental impairment and progressive renal dysfunction. The underlying mechanism may involve dysregulation of β‐catenin/Wnt signaling, contributing to the complex phenotype [56, 113].

Kleefstra syndrome and autism spectrum disorder represent additional neurodevelopmental conditions linked to Zn‐related pathways. Kleefstra syndrome is a 9q34 microdeletion resulting in haploinsufficiency of *EHMT1*, a repressive histone methyltransferase containing a Zn‐binding SET domain [69]. Loss of *EHMT1* leads to enhanced expression of neuronal genes, including the nuclear receptor 1 subunit of the NMDA receptor, a process strongly implicated in autism spectrum disorder‐related synaptic dysfunction [69, 114]. Furthermore, SHANK3, a postsynaptic density protein essential for glutamatergic synapse integrity, is stabilized by Zn. Experimental models have demonstrated that Zn supplementation can rescue defective synaptic architecture in *SHANK3* deficiency [66, 67, 68, 69, 114].

Wilson's disease, although primarily a disorder of Cu metabolism, also involves Zn from a therapeutic perspective. This autosomal recessive condition is caused by loss‐of‐function mutations in *ATP7B*, which encodes a Cu‐transporting P‐type ATPase essential for biliary Cu excretion from hepatocytes. Defective *ATP7B* function resulted in progressive hepatic Cu accumulation, leading to hepatic cirrhosis, neuropsychiatric manifestations, and Kayser–Fleischer rings [9, 12, 56, 57].

PSTPIP1‐associated myeloid‐related proteinemia inflammatory syndrome (PAMI syndrome) presents a paradoxical combination of markedly elevated circulating Zn concentrations and concurrent functional Zn deficiency at the tissue level. Excessive calprotectin (MRP8/14) in the circulation sequestered Zn within nonbioavailable complexes, thereby depleting exchangeable Zn pools in tissues despite apparent hyperzincemia [115, 116].

Beyond these specific disorders, several other clinical conditions may present with low Zn levels. These include human immunodeficiency virus infection (reduced absorption) [117], chronic kidney disease (inadequate dietary intake, malabsorption, and Zn removal during hemodialysis) [118], liver diseases (poor diet and increased urinary excretion) [62, 119], polycystic ovary syndrome (high estrogen levels decreasing plasma Zn) [62, 120], and sickle cell disease or beta thalassemia (Fe chelation leading to Zn deficiency) [121, 122].

The diagnosis of Zn deficiency relies on a combination of clinical assessment, biochemical testing, and, in selected cases, genetic analysis. Serum Zn concentration is the most widely used laboratory marker, with low serum ALP activity serving as a useful adjunctive marker [72]. For detailed diagnostic criteria, preanalytical considerations, and genetic testing indications, refer to Section 5.3.

#### 3.3.2 Zn Overload

To date, no cases of Zn overload or toxicity due to mutations in Zn transporters have been reported. All cases of Zn toxicity are acquired rather than inherited [19, 70, 72, 103, 104, 105]. Causes include pesticide exposure, exposure to compounds used in paints, rubber, and dyes, occupational exposure to Zn, overprescribing of Zn‐containing medications, Zn present in dental fixtures, and overconsumption of over‐the‐counter Zn supplements [19, 70, 72, 103, 104, 105, 123]. The most common cause of Zn excess is excessive supplementation [19, 70, 72, 103, 104, 105]. According to the US Institute of Medicine, the tolerable upper intake levels for Zn are 4 mg for infants, 12 mg for children aged 4–8 years, 34 mg for adolescents aged 14–18 years, and 40 mg for persons aged 19 years or older [33]. Acute and chronic Zn toxicities are defined as intakes exceeding 200 and 50–150 mg/day, respectively [19, 70, 72, 103, 104, 105]. At doses above 50 mg/day, symptoms such as nausea, diarrhea, and abdominal discomfort may occur, whilst doses exceeding 150 mg/day can adversely affect the body's lipid profile and immune system. Symptoms of Zn toxicity may not manifest until intake exceeds 1–2 g [124, 125]. The clinical presentation of Zn toxicity varies according to the mode of overload. Acute dietary ingestion presents with nausea, vomiting, diarrhea, and muscle cramps. Inhalation of Zn fumes (resulting in metal fume fever) presents with flu‐like symptoms including cough, fever, and chills. Chronic ingestion slowly leads to a syndrome of neuropathy, anemia, fatigue, and spasticity [124, 125].

An important complication of chronic Zn toxicity is ZICD. The pathophysiological mechanism involves excess Zn within the intestinal lumen, which upregulates metallothionein expression in enterocytes. Because Cu has a greater affinity for metallothioneins than Zn does, Cu preferentially binds to these proteins and is subsequently lost via enterocyte sloughing, resulting in decreased Cu absorption and eventual Cu deficiency [126]. The coexistence of hyperzincemia (elevated serum Zn) and hypocupremia (low serum Cu) is highly suggestive of ZICD [127]. ZICD typically develops insidiously over months to years [128]. Early manifestations include neutropenia, leukopenia, and anemia [127]. If left undiagnosed, the condition can progress to severe and permanent neurological complications, including gait disturbances, paresthesia, and myelopathy [129]. Indeed, one study highlighted that inappropriate Zn overprescription was a significant cause of ZICD, underscoring the potentially serious consequences of injudicious Zn use  [128].

Zn plays diverse roles in neurodegenerative diseases. In Alzheimer's disease, Zn participates in amyloid precursor protein processing through members of the MetZnin family, particularly ADAM10 and ADAM17, which compete with β‐ and γ‐secretase complexes for amyloid precursor protein cleavage [62, 130, 131]. Epidemiological studies have associated higher dietary intake of Zn, vitamin B_12_, and polyunsaturated fatty acids with reduced Alzheimer's disease incidence [130]. In Parkinson's disease, Huntington's disease, amyotrophic lateral sclerosis, and prion disorders, impaired lysosomal acidification and deficient autophagy–lysosomal pathway activity contribute to protein aggregation. Zn availability is critical for maintaining vacuolar‐type ATPase integrity and autophagic flux in these conditions [132, 133, 134].

Aging is accompanied by a measurable decline in systemic and cellular Zn status, which has been implicated in metabolic dysregulation, cardiovascular disease, immune system deterioration, neurodegenerative disorders, and several malignancies [132, 135, 136, 137].

### 3.4 Ca‐Associated Diseases

Dysregulation of mitochondrial Ca signaling contributes to a range of pathological processes. Abnormal Ca handling promotes mitochondrial membrane permeabilization, leading to the release of either mitochondrial damage‐associated molecular patterns and mitochondria‐derived vesicles that drive and sustain inflammation, or cytochrome *c* and proapoptotic factors that activate caspases and trigger apoptosis, thereby exacerbating disease progression [22, 73] (Figure 2). Ca‐associated diseases can be broadly categorized into those resulting from insufficient Ca availability (Ca deficiency) and those arising from excessive Ca accumulation or dysregulated Ca signaling (Ca overload).

#### 3.4.1 Ca Deficiency

Ca deficiency, or hypocalcemia, typically results from inadequate dietary Ca intake, vitamin D deficiency, parathyroid hormone (PTH) deficiency or resistance, or chronic kidney disease. Hypocalcemia manifests predominantly as neuromuscular irritability (paresthesia, tetany, Chvostek/Trousseau signs) [23, 79] and, if chronic, may cause cataracts and dental anomalies [138] (Table 1).

At the mitochondrial level, the ability of mitochondria to buffer cytosolic Ca^2+^ levels declines with age [22]. This impaired buffering can lead to prolonged elevation of intracellular Ca, rather than deficiency, but the net effect may mimic functional Ca deficiency at specific signaling nodes. This dysregulation is localized to ER–mitochondria contact sites, where age‐related reductions in mitochondrial Ca uptake, driven by alterations in MCU complex components and their regulatory proteins, compromise buffering and promote cytosolic Ca overload [74, 75, 76]. The sustained rise in Ca activates Ca‐dependent enzymes, including calpains, phospholipase A, and Ca/Mg‐dependent endonucleases, which degrade cytoskeletal proteins, lipid membranes, and DNA, thereby exacerbating injury and amplifying inflammatory signaling [77, 78].

The diagnosis of hypocalcemia requires biochemical confirmation using albumin‐corrected total Ca (typically <8.5 mg/dL or 2.12 mmol/L) or ionized Ca, with the latter representing the biologically active form [23, 79, 80]. Concurrent measurement of serum phosphate, magnesium, PTH, and 25‐hydroxyvitamin D is essential for determining the underlying etiology: inappropriately low or normal PTH indicates hypoparathyroidism, whereas elevated PTH suggests vitamin D deficiency, chronic kidney disease, pseudohypoparathyroidism (PHP), or magnesium abnormalities. Serum magnesium should always be measured, as hypomagnesemia is a common reversible cause [79, 81, 82]. Clinical assessment should identify neuromuscular manifestations including perioral and digital paresthesia, muscle cramps, tetany, Chvostek's sign, Trousseau's sign and, in severe cases, seizures or QT prolongation on electrocardiogram [23, 79, 80]. In patients with suspected hereditary disorders, particularly those presenting at a young age or with syndromic features, genetic testing for *CASR*, *GNAS*, *AIRE*, or *22q11.2* deletion should be considered [58, 139, 140, 141]. Early diagnosis and prompt treatment are essential to prevent irreversible neurological complications [79, 81].

#### 3.4.2 Ca Overload

Ca overload, or dysregulated intracellular Ca handling, is implicated in a wide spectrum of diseases, including neurodegenerative disorders, cancer, cystic fibrosis, cardiovascular diseases, and inflammatory conditions of the gut and central nervous system.

Ca dysregulation plays a key role in neurodegenerative diseases. In Alzheimer's disease, mitochondrial impairment is an early and prominent feature, with Ca overload observed in affected neurons [142]. Amyloid‐β accumulation induces oxidative stress and enhances mitochondrial ROS production, which in turn exacerbates mitochondrial damage [143]. *MICU3* overexpression has been shown to rescue mitochondrial dysfunction, reducing oxidative stress, restoring mitochondrial membrane potential, and elevating ATP production. Notably, these protective effects of *MICU3* are PINK1‐dependent, as knockdown of *PINK1* abolished MICU3's benefits [144]. In Parkinson's disease, mitochondrial Complex I deficiency and mutations in genes regulating mitochondrial quality control, including *PINK1* and *Parkin*, key regulators of mitochondrial quality control, leads to the accumulation of depolarized mitochondria and elevated ROS production [145]. *Parkin* loss promoted microglial NLRP3 inflammasome activation and contributes to neuronal damage and loss [145]. Furthermore, the cytosolic DNA sensor cGAS, through activation of the cGAS–STING pathway in microglia, amplifies neuroinflammation and dopaminergic neurodegeneration in Parkinson's disease models [146].

Ca dysregulation also contributes to proliferative syndromes like cancer. In oncogenesis, mitochondrial Ca signaling plays a significant role: oncogenic transformation was accompanied by marked alterations in mitochondrial membrane potential and Ca handling, with a concomitant increase in ROS production [147]. In the tumor microenvironment (TME), hypoxia stabilized of HIF‐1α [148] and elevated intracellular Ca levels, often through altered activity of Ca channels and transporters  [149, 150, 151]. This rise in Ca activated CaMKII [152], which in turn stimulated the IKK complex and activated NF‐κB [153]. Furthermore, HIF‐1α upregulated Ca effectors such as TRPC1 and STIM1, reinforcing Ca entry and further stabilizing HIF‐1α in a self‐perpetuating loop [149]. This mutual reinforcement between Ca signaling and HIF‐1α enhanced the transcription of proinflammatory genes, thereby contributing to a sustained inflammatory state in the TME [153]. In breast cancer, loss of *ULK1* led to impaired mitophagy and increased mitochondria ROS (mtROS), which activated the NLRP3 inflammasome and promoted secretion of interleukin‐1β (IL‐1β) and IL‐6, fostering bone metastasis [154]. Promyelocytic leukemia protein localized at ER–mitochondria contact sites, forming a trimeric complex with NLRP3 and the P2×7 receptor, thereby modulating inflammasome activation and shaping the TME [155]. In cystic fibrosis, disturbed Ca signaling between the ER and mitochondria enhanced mitochondrial Ca uptake via MCU, activating the NLRP3 inflammasome and promoting IL‐1β and IL‐18 secretion [156, 157, 158, 159]. Pharmacologic MCU inhibition restored autophagy and immune function in cystic fibrosis models, highlighting MCU as a promising target to modulate inflammation [160].

Ca dysregulation is also a key driver of cardiovascular diseases, particularly ischemia/reperfusion (I/R) injury [161, 162]. During ischemia, intracellular Ca accumulates due to impaired ATP‐dependent pumps and altered ion exchange. Upon reperfusion, the sudden influx of O_2_ and restoration of pH exacerbate Ca^2+^ overload, especially within mitochondria. Excessive mitochondrial Ca uptake triggered mPTP opening, leading to mitochondrial depolarization, ATP depletion, swelling, and release of proapoptotic factors culminating in cell death [163, 164]. MICU1 functions as a gatekeeper of mitochondrial Ca levels and influences ROS production through downregulation of SIRT3. *MICU1* deficiency led to unregulated Ca influx into mitochondria, triggering mitochondrial dysfunction and inflammation [165]. Similarly, *MICU2* deficiency resulted in excessive mitochondrial Ca accumulation, prompting ROS production, mPTP opening, and activation of inflammatory pathways [166]. Hyperhomocysteinemia, a recognized cardiovascular risk factor, promoted ER stress and enhanced ER–mitochondria contact sites, facilitating rapid Ca transfer to mitochondria in macrophages. Consequently, mitochondrial ROS production and mtDNA release were increased, ultimately driving macrophage pyroptosis and amplifying inflammation [167].

Ca dysregulation also underlies other inflammatory diseases, including intestinal inflammation induced by the mycotoxin citrinin, inflammatory bowel disease (IBD), and multiple sclerosis. In citrinin‐induced intestinal inflammation, activation of the IP_3_R1–GRP75–VDAC1 ER–mitochondria tethering complex promotes mitochondrial Ca overload, increased mtROS, and subsequent pyroptosis of intestinal epithelial cells. Treatment with Koumine significantly attenuates citrinin‐induced intestinal damage by downregulating the expression or activation of the IP_3_R1–GRP75–VDAC1 complex, thereby decreasing mitochondrial Ca uptake [168]. In IBD, mitochondrial dysfunction is a central pathogenic mechanism. Variants in mitophagy‐related genes (*ATG16L1*, *IRGM*, *LRRK2*) impaired mitochondrial quality control, resulting in accumulation of dysfunctional mitochondria and excessive mtROS production [169, 170, 171]. Proinflammatory cytokines TNFα and IL‐1β, upregulated in IBD, targeted mitochondria to inhibit ETC Complex I and increase mtROS production [172, 173]. Anti‐TNFα therapy has demonstrated protective effects against mitochondrial dysfunction in the intestine [174]. In multiple sclerosis, inflammatory conditions impaired oligodendrocyte differentiation by disrupting mitochondrial function, including altered mitochondrial Ca uptake, reduced membrane potential, decreased Complex I activity, and elevated ROS levels [175].

### 3.5 Summary

The pathological consequences of metal imbalance mirror the diversity of their physiological roles. Fe deficiency imposes a major global health burden, compromising cognitive development, immunity, and maternal–fetal health; conversely, Fe overload unleashes ferroptosis‐driven damage in the liver, heart, and endocrine glands, manifesting as hypogonadism, diabetes, and cardiomyopathy. Cu deficiency, although rare, causes hematological and neurological syndromes that can become irreversible without early intervention; in contrast, Cu overload in Wilson's disease and related conditions triggers cuproptosis. Zn deficiency, whether acquired or inherited, presents with growth failure, immune dysfunction, dermatological lesions, and neurodevelopmental impairment; specific transporter mutations cause distinct syndromes, including acrodermatitis enteropathica and Birk–Landau–Perez syndrome. Chronic Zn toxicity, though uncommon, precipitates ZICD, a potentially permanent neurological disorder that highlights the competitive dynamics between these two metals. Ca dysregulation, whether hypo‐ or hypercalcemia, underpins a broad range of conditions, from hypoparathyroidism and osteoporosis to ischemic heart disease, IBD, and neurodegeneration, with mitochondrial Ca handling being increasingly recognized as a key determinant of cell fate (Table 1).

Across these four metals, common principles emerge: a narrow therapeutic window between deficiency and toxicity; reliance on tissue‐specific homeostatic control; convergence of metal dysregulation on shared cell death pathways (ferroptosis, cuproptosis, and Ca‐dependent apoptosis); and inflammation amplified by mitochondrial damage signals. These mechanistic insights both explain disease pathogenesis and suggest novel therapeutic avenues. The following section transitions from mechanisms to risk factors, exploring the predisposing conditions that translate metal imbalance into clinical disease and linking fundamental biology to preventive care.

## 4 Risk Factors for Metal Imbalance

The disruption of metal ion homeostasis arises from a complex interplay of dietary, environmental, genetic, and demographic factors. Understanding these risk determinants is essential for identifying vulnerable populations, establishing preventive strategies, and guiding precision medicine approaches in metal‐related disorders.

The pathological consequences of metal imbalance detailed in Section 3 do not arise in isolation but are precipitated by a complex interplay of risk factors that operate across the lifespan. Understanding these risk determinants is essential for identifying vulnerable populations, establishing preventive strategies, and guiding precision medicine approaches in metal‐related disorders. The disruption of metal ion homeostasis can be traced to four major categories of risk factors. Dietary factors (Section 4.1) represent the primary source of essential metals, yet both inadequate intake and excessive supplementation can lead to disease. Environmental exposure (Section 4.2), through air, water, soil, and occupation, introduces additional metal burdens, particularly from nonessential toxic elements. Genetic predisposition (Section 4.3), including mutations in metal transporters and regulators (e.g., *HFE, ATP7B, SLC39A4*), can confer susceptibility even in the absence of dietary or environmental triggers. Finally, age, sex, and comorbidity (Section 4.4) modulate metal absorption, distribution, and excretion, shaping individual risk profiles and often exacerbating genetic or environmental vulnerabilities. Accordingly, this section systematically examines each of these four domains, providing a framework for risk assessment that bridges the pathophysiological insights of Section 3 with the diagnostic approaches of Section 5.

### 4.1 Dietary Factors

Dietary intake is the predominant source of essential metal ions for humans, yet it represents a double‐edged sword. Insufficient intake precipitates deficiency syndromes, whereas excessive intake, whether from oversupplementation or contaminated food, leads to toxic accumulation. The therapeutic window for essential metals is narrow, and deviations at either extreme carry substantial health risks.

Deficiency‐related dietary risks predominantly affect populations with limited access to metal‐enriched foods, such as red meat (Fe and Zn), seafood and nuts (Cu and Zn), and dairy products (Ca). Fe deficiency affects roughly one in four individuals globally, with the highest burden observed in children, women of reproductive age, and residents of low‐ and middle‐income countries [14]. Zn deficiency similarly stems from inadequate intake, particularly in regions where cereal‐based diets prevail, because phytates in grains impede Zn absorption [176]. Cu deficiency, although less common, may arise from chronic malnutrition or prolonged total parenteral nutrition lacking adequate Cu supplementation [177].

Overload‐related dietary risks typically involve excessive supplementation or consumption of metal‐contaminated foods. Chronic high‐dose Zn supplementation (exceeding 40 mg/day in adults) is a well‐recognized cause of Cu‐deficiency myelopathy, as excess Zn upregulates intestinal metallothionein expression, which sequesters Cu and prevents its absorption [128]. Dietary Fe overload is rare in individuals without genetic predisposition; however, HH, the most common genetic disorder among populations of European descent, causes unregulated intestinal Fe absorption even with normal dietary intake, leading to progressive parenchymal Fe deposition in the liver, heart, and endocrine glands [16].

The deficiency‐overload paradox is particularly striking for essential metals: the same homeostatic mechanisms that guard against deficiency can, when dysregulated, precipitate overload. Hepcidin, the master regulator of Fe metabolism, is suppressed in Fe deficiency to enhance absorption, but is inappropriately low in HH, resulting in hyperabsorption

The paradox of deficiency versus overload is particularly pronounced for essential metals, in that the same homeostatic pathways that avert insufficiency can, upon dysregulation, engender pathological accumulation. Hepcidin, the principal regulator of Fe homeostasis, is downregulated in states of Fe deficiency to augment intestinal absorption; however, in HH, hepcidin remains inappropriately suppressed, resulting in unregulated Fe uptake [16]. By analogy, Cu homeostasis is governed by a delicate interplay between CTR1 and ATP7B; loss‐ or gain‐of‐function mutations in these transporters produce either Menkes disease (Cu deficiency) or Wilson's disease (Cu overload) [178].

### 4.2 Environmental Exposure

Environmental exposure to metal ions occurs through multiple pathways, including air, water, soil, and food, and constitutes a major global public health concern. In contrast to dietary intake, environmental exposure predominantly involves nonessential toxic metals (e.g., lead, cadmium, mercury, and arsenic) that serve no biological function and accumulate progressively in the body over time.

Water and soil contamination represent another critical exposure route. Industrial discharges, mining activities, and agricultural runoff release metals such as cadmium, lead, and arsenic into water sources and soil, from which they subsequently enter the food chain [179]. Groundwater arsenic contamination, endemic in parts of South and Southeast Asia, remains a leading cause of chronic arsenic poisoning, manifesting as skin lesions, peripheral neuropathy, and an elevated risk of cancer [179].

Ambient air pollution constitutes a significant pathway for metal exposure, particularly in urban and industrial areas. A comprehensive systematic review of heavy metals in indoor air reported that Zn dominated indoor metal concentrations, accounting for 69.72% of total metals, with primary sources being traffic emissions and tobacco smoking [180]. Rural areas exhibited elevated arsenic and lead levels owing to solid‐fuel combustion for cooking and heating, with concentrations one to two orders of magnitude higher than those in urban settings [180]. Health risk assessments revealed that residents in Southeast and East Asia faced noncarcinogenic risks from multiple heavy metals, driven chiefly by solid‐fuel use and industrial emissions [180].

Occupational exposure affects workers in high‐risk industries, including mining, smelting, welding, and battery manufacturing. Inhalation of metal‐containing fumes or dusts results in higher systemic metal burdens than environmental exposure. For instance, welders exposed to manganese fumes are at risk of manganism, a Parkinsonian syndrome; Cu smelter workers may develop Cu toxicity; and chronic cadmium exposure in battery manufacturing causes nephropathy and bone demineralization [179].

The toxicological impact of environmental metal exposure depends on multiple factors, such as the specific metal, dose and duration of exposure, route of entry, chemical speciation, and individual susceptibility [179]. Importantly, humans are rarely exposed to single metals; rather, they are exposed to complex mixtures, and synergistic or antagonistic interactions among metals can modulate overall toxicity [179, 181].

Children represent a particularly vulnerable population with respect to environmental metal exposure. Their developing organs, higher intake of air and food per unit body weight, and frequent hand‐to‐mouth behavior increase metal absorption and retention [182, 183, 184, 185]. Lead exposure, even at low levels, impairs neurodevelopment, reducing intelligence quotient and heightening the risk of attention deficits [186]. Cadmium and arsenic exposure during development has been linked to impaired cognitive function and an increased risk of cancer later in life [182, 183, 184, 185].

### 4.3 Genetic Predisposition

Inherited mutations in genes that govern metal ion transport, storage, and excretion constitute a major risk factor for metal imbalance disorders. In contrast to dietary or environmental factors, genetic predisposition typically manifests earlier in life and follows distinct inheritance patterns.

Fe‐related genetic disorders are exemplified by HH, an autosomal recessive condition most commonly caused by mutations in the *HFE* gene (C282Y and H63D variants) [16]. These mutations result in inappropriately low hepcidin levels, permitting unregulated intestinal Fe absorption regardless of body Fe stores. Non‐*HFE* forms of HH, arising from mutations in *HJV*, *HAMP*, *TFR2*, and *SLC40A1*, typically present earlier and with more severe clinical phenotypes [16]. Importantly, the penetrance of *HFE*‐related HH is incomplete; only a subset of C282Y homozygotes developed end‐organ damage, suggesting that additional genetic and environmental modifiers influence disease expression [16].

Cu‐related genetic disorders are predominantly attributable to mutations in the P‐type ATPases ATP7A and ATP7B. Loss‐of‐function mutations in *ATP7B* cause Wilson's disease, an autosomal recessive condition in which Cu accumulates pathologically in the liver and brain. To date, over 500 *ATP7B* mutations have been described, giving rise to a broad clinical spectrum that encompasses hepatic (hepatitis, cirrhosis, acute liver failure), neurological (dystonia, tremor, dysarthria), and psychiatric presentations [178, 187]. Conversely, *ATP7A* mutations underlie Menkes disease, an X‐linked recessive disorder that manifests in infancy with severe Cu deficiency, neurodegeneration, and connective tissue involvement; milder alleles give rise to occipital horn syndrome [178, 187].

Zn‐related genetic disorders encompass acrodermatitis enteropathica, caused by mutations in *SLC39A4*, which encodes the ZIP4 Zn transporter. This autosomal recessive condition impairs intestinal Zn absorption and produces the classic triad of alopecia, diarrhea, and dermatitis [9]. Mutations in *SLC30A2* cause TNZD, wherein affected mothers secrete Zn‐deficient breast milk [188]. Furthermore, mutations in other Zn transporters, including *ZIP8* (associated with cortical atrophy and intellectual disability), *ZIP13* (spondylocheirodysplastic Ehlers–Danlos syndrome), and *ZnT9* (Birk–Landau–Perez syndrome), have been linked to distinct clinical syndromes, underscoring the expanding spectrum of Zn‐related genetic disorders [9].

Ca‐related genetic disorders are primarily associated with mutations in genes regulating Ca sensing, transport, and intracellular signaling. CASR, a G protein‐coupled Ca‐sensing receptor expressed in parathyroid glands and renal tubules, plays an essential role in maintaining mineral ion homeostasis by sensing extracellular Ca concentrations and modulating PTH secretion [189, 190]. Gain‐of‐function mutations in *CASR* caused autosomal dominant hypocalcemia Type 1 (ADH1), characterized by inappropriately low PTH levels, hypocalcemia, and hypercalciuria [189, 190]. Conversely, loss‐of‐function *CASR* mutations led to familial hypocalciuric hypercalcemia (FHH), a generally benign condition of mild‐to‐moderate hypercalcemia, whereas homozygous mutations cause neonatal severe hyperparathyroidism [189, 190]. A comprehensive database recently catalogued 498 CASR variants, of which 121 have been associated with ADH1 and 377 with FHH1 [189]. Mutations in* TRPV6*, an epithelial Ca channel essential for placental Ca transport, caused transient neonatal hyperparathyroidism with severe skeletal demineralization, bone deformities, and elevated PTH levels [191, 192]. Biallelic loss‐of‐function *TRPV6* variants disrupted maternal–fetal Ca transport, leading to fetal Ca deficiency and defective bone mineralization, which typically resolves with Ca supplementation during early infancy [191, 192]. Additionally, mutations in *SLC34A1* (encoding the sodium‐phosphate cotransporter NPT2a) caused infantile hypercalcemia Type 2, presenting with hypophosphatemia, elevated 1,25‐dihydroxyvitamin D_3_, hypercalcemia, and hypercalciuria [193, 194]. *CLDN16* mutations, which disrupt Claudin‐16‐mediated tight junction function in the renal thick ascending limb, caused familial hypomagnesemia with hypercalciuria and nephrocalcinosis, characterized by severe renal Ca and magnesium wasting [195, 196]. Beyond monogenic disorders, polymorphisms in *VDR*, a gene encoding vitamin D receptor, have been linked to altered serum Ca levels, hypercalciuria, and an increased risk of nephrolithiasis, underscoring the polygenic contribution to Ca homeostasis [195, 196].

The emerging field of metallo‐genomics aims to systematically identify genetic variants that modify metal homeostasis and disease susceptibility. Genome‐wide association studies have linked polymorphisms in metal‐related genes to altered trace element levels, and ongoing research seeks to integrate metallomics with genomics to enable personalized risk assessment and targeted prevention [16, 178].

### 4.4 Demographic Factors

Demographic factors profoundly influence metal homeostasis and the risk of metal imbalance disorders, often through interactions with dietary, environmental, and genetic determinants.

#### 4.4.1 Age

Age affects metal homeostasis at multiple levels. Infants and young children have heightened requirements for essential metals (Fe, Zn, Cu) to support rapid growth and neurodevelopment; however, their immature homeostatic regulatory mechanisms and lower body stores increase their susceptibility to deficiency. Conversely, children absorb and retain environmental lead more efficiently than adults, and their developing brains are particularly vulnerable to lead‐induced neurotoxicity [182, 183, 184, 185]. In the elderly, deficiency risk is elevated owing to reduced dietary intake, impaired absorption (e.g., atrophic gastritis diminishing Fe and vitamin B_12_ absorption), and polypharmacy. Furthermore, age‐related decline in mitochondrial function may compromise cellular metal handling, potentially contributing to the accumulation of redox‐active metals in age‐associated neurodegenerative disorders [178].

#### 4.4.2 Sex

Both biological sex and gender‐related factors exert significant effects on metal homeostasis. Biological sex influences metal metabolism primarily through hormonal regulation and differential physiological losses. Premenopausal women have higher Fe requirements, approximately 18 mg/day compared with 8 mg/day in men, owing to menstrual blood loss, rendering Fe deficiency substantially more prevalent in this population [14]. Pregnancy further elevates the demands for Fe and Zn to support fetal development and placental transfer [197, 198]. Estrogen also influences Cu metabolism, as serum Cu and ceruloplasmin levels rise during pregnancy and with oral contraceptive use [199]. In contrast, men are more susceptible to Fe overload disorders such as HH, as women are partially protected by menstrual Fe losses until menopause [16]. Gender‐related differences in occupational exposure patterns also contribute to disparities: men are more likely to work in mining, smelting, and welding, whereas women may face higher exposure through domestic cooking with contaminated fuels [179, 182, 183, 184, 185].

#### 4.4.3 Comorbidity

Comorbidities substantially modulate metal homeostasis and disease risk through diverse mechanisms that affect metal absorption, distribution, excretion, and tissue deposition.

Chronic kidney disease impairs the renal excretion of multiple metals, particularly those cleared primarily by the kidneys, such as Zn and chromium. In patients undergoing hemodialysis, metal balance may be further perturbed: dialysate water purity determines whether metals are removed from or inadvertently introduced into the circulation, with aluminum and Cu contamination remaining a concern in resource‐limited settings [178].

Liver disease disrupts the synthesis of metal‐binding proteins, including ceruloplasmin (Cu), transferrin (Fe), and metallothioneins (Zn and Cu), and also impairs biliary excretion, the primary route for Cu and manganese elimination. Consequently, chronic liver disease predisposes patients to Cu accumulation and manganese neurotoxicity, while simultaneously altering systemic Fe distribution [178].

Gastrointestinal disorders, including coeliac disease, Crohn's disease, and bariatric surgery, impair metal absorption from the intestinal lumen, leading to deficiency syndromes of Fe, Zn, and Cu. In coeliac disease, villous atrophy reduces the absorptive surface area, whereas bariatric procedures bypass critical segments of the small intestine where metal absorption predominantly occurs [14, 98, 99].

Diabetes mellitus exemplifies a bidirectional relationship between comorbidity and metal homeostasis. On one hand, Fe overload increases the risk of Type 2 diabetes through Fe‐mediated oxidative damage to pancreatic β‐cells via Fenton chemistry, resulting in impaired insulin secretion [16, 89, 200]. On the other hand, diabetes‐associated hyperglycemia and oxidative stress can alter Zn and Cu distribution. Zn deficiency is commonly observed in diabetic patients owing to hyperzincuria (increased urinary Zn excretion), which may contribute to delayed wound healing and immune dysfunction [201]. Furthermore, metformin, a first‐line antidiabetic medication, interferes with vitamin B_12_ and possibly folate metabolism, although its direct effects on Zn and Cu homeostasis require further investigation [202, 203]. This bidirectional interplay underscores the importance of monitoring metal status in diabetic patients and considering metal dyshomeostasis as a potential contributor to diabetic complications, including cardiovascular disease and neuropathy [200].

Inflammatory states, including chronic infections and autoimmune diseases, induce hepcidin expression via IL‐6 signaling. Elevated hepcidin promotes internalization and degradation of ferroportin, the sole cellular Fe exporter, leading to functional Fe‐deficiency anemia despite adequate body Fe stores. This condition, known as anemia of chronic disease, is typically normocytic and normochromic, distinguishing it from true Fe‐deficiency anemia  [178].

Collectively, these comorbid conditions highlight the need for integrated clinical assessment that considers metal homeostasis alongside primary disease management.

#### 4.4.4 Geographic and Socioeconomic Factors

Geographic and socioeconomic factors interact with age, sex, and comorbidities to shape the overall burden of metal‐related diseases. Populations in low‐ and middle‐income countries are particularly vulnerable, facing elevated risks of both nutritional deficiencies, owing to limited access to metal‐enriched foods, and toxic exposures resulting from insufficient environmental regulation. This dual burden of malnutrition, manifesting as concurrent deficiency and overload, is increasingly recognized in rapidly industrializing nations, where traditional cereal‐based diets often lack bioavailable Fe and Zn, while industrial pollution introduces toxic metals into the environment [14, 179].

### 4.5 Summary

In summary, the risk factors for metal imbalance are multifaceted and interconnected, encompassing dietary inadequacies, environmental toxicants, genetic susceptibilities, and demographic characteristics. Deficiencies most commonly arise from insufficient dietary intake, malabsorption, or increased physiological demands, with children, pregnant women, and the elderly being particularly affected. Overload states, conversely, may result from excessive supplementation, occupational or environmental exposure, or inherited mutations in metal transporters such as *HFE* (Fe), *ATP7A/B *(Cu), *SLC39A4* (Zn), and *CASR* (Ca). Notably, the same homeostatic mechanisms, that is, hepcidin for Fe, and metallothioneins for Zn and Cu, can paradoxically predispose to overload when genetically dysregulated, illustrating the deficiency‐overload paradox. Age and sex further modulate risk: premenopausal women are protected from Fe overload but vulnerable to deficiency, whereas men face higher risks of HH. Comorbidities, including chronic kidney disease, liver disease, diabetes mellitus, and inflammatory states, can exacerbate metal imbalances through impaired excretion, altered synthesis of binding proteins, or hepcidin‐mediated Fe sequestration. Geographically, low‐ and middle‐income countries bear a double burden of malnutrition and environmental pollution, driving concurrent deficiency and toxicity. Recognizing these risk factors is not merely an academic exercise; it is a clinical imperative that enables early identification of at‐risk individuals, targeted prevention strategies, and personalized monitoring. The diagnostic approaches that translate this risk assessment into confirmatory testing and clinical decision‐making are the focus of the following section (Figure 3).

**FIGURE 3:**
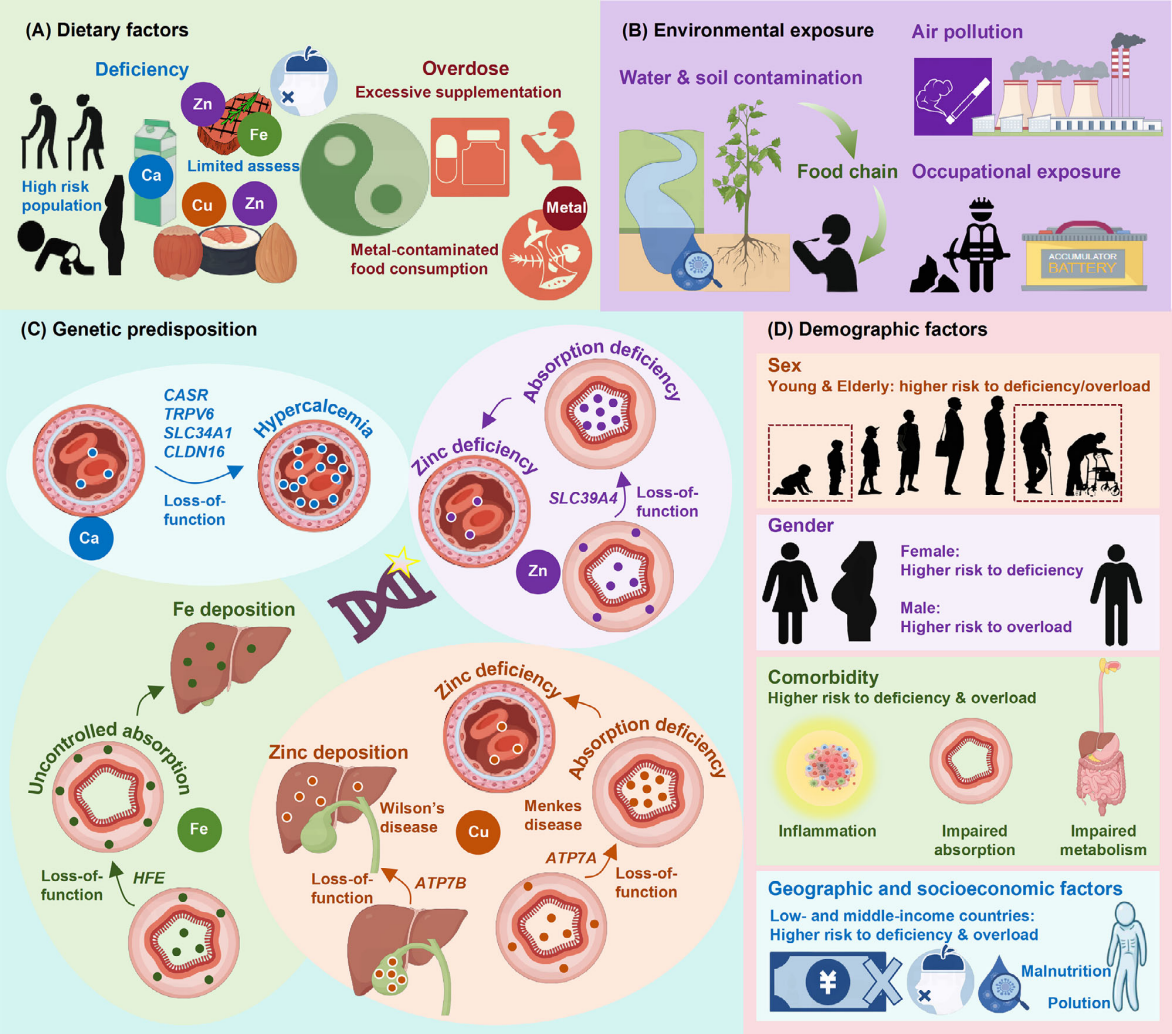
Risk factors for metal‐related diseases. Four major categories are illustrated. (A) Dietary factors cause both deficiency (limited intake of essential metals such as Fe, Cu, Zn, Ca, especially in children, elderly, and pregnant women) and overload (excessive supplementation or consumption of contaminated food). (B) Environmental exposure disrupts metal homeostasis via three routes: food‐chain transfer from contaminated soil/water, inhalation of airborne metallic pollutants, and occupational exposure in high‐risk groups (e.g., miners, battery workers). (C) Genetic predisposition involves mutations in metal transporters/regulators: *HFE* loss‐of‐function leads to hereditary hemochromatosis (Fe overload); *ATP7A* mutations cause Menkes disease (Cu deficiency), *ATP7B* mutations cause Wilson's disease (Cu overload); *SLC39A4* mutations cause acrodermatitis enteropathica (Zn deficiency); and mutations in *CASR*, *TRPV6*, *SLC34A1*, or *CLDN16* result in hypercalcemia. (D) Demographic factors include age (infants and elderly prone to both deficiency and toxicity due to immature/declining homeostasis), sex (premenopausal women at higher Fe‐deficiency risk, men more prone to Fe overload), comorbidities (chronic kidney/liver disease, diabetes, inflammation impair absorption/metabolism and induce hepcidin‐mediated Fe sequestration), and geographic/socioeconomic conditions (low‐/middle‐income countries face double burden of malnutrition and environmental pollution, increasing both deficiency and toxicity risks). ATP7A, ATPase copper transporting alpha; ATP7B, ATPase copper transporting beta; Ca, calcium; CASR, calcium‐sensing receptor; CLDN16, Claudin 16; Cu, copper; Fe, iron; HFE, homeostatic iron regulator; HH, hereditary hemochromatosis; SLC34A1, solute carrier family 34 member 1; SLC39A4, solute carrier family 39 member 4; TRPV6, transient receptor potential vanilloid 6; Zn, zinc.

## 5 Diagnostic Approaches for Metal‐Related Disorders

Building upon the multifactorial risk factors for metal imbalance discussed in the previous section, the accurate diagnosis of metal‐related disorders represents the essential bridge between risk identification and targeted therapeutic intervention. The diagnostic approach varies substantially across metals, reflecting their distinct physiological roles, homeostatic mechanisms, and the nature of the imbalance (deficiency versus overload). For Fe, the diagnostic paradigm distinguishes deficiency, the most prevalent micronutrient deficiency globally, from overload conditions such as HH and transfusion‐dependent Fe accumulation. Cu disorders span acquired deficiency, often due to malabsorption or excessive Zn ingestion, to inherited overload syndromes, most notably Wilson's disease. Zn diagnosis distinguishes deficiency from toxicity, the latter being always acquired and characterized by ZICD. Ca disorders require differentiation of systemic Ca dysregulation (hypocalcemia vs. hypercalcemia) from crystal deposition diseases such as CPPD. Accordingly, this section is structured into four subsections, each organized by clinical assessment, biochemical testing, imaging, and genetic analysis, providing a practical framework for clinicians to confirm specific etiologies and guide subsequent management (Table 2).

**TABLE 2:** Diagnostic approaches to disorders of Fe, Cu, Zn, and Ca.

Metal	Disorder	Clinical features	Biochemical testing	Imaging	Genetic analysis
Fe	Deficiency	Fatigue, pallor, pica, glossitis, angular cheilitis, restless legs syndrome, pagophagia (ice craving), neurodevelopmental delay in children Data sources: [14]	Low serum ferritin (<30 µg/L); low transferrin saturation (<20%); low serum Fe; elevated TIBC; low hepcidin Data sources: [14]	Not routinely indicated; bone marrow Fe aspiration (gold standard but invasive) Data sources: [14]	Indicated only if hereditary forms suspected (e.g., *TMPRSS6* mutations in Fe‐refractory Fe deficiency anemia) Data sources: [14]
	Overload (hemochromatosis)	Fatigue, arthralgia (MCP joints), skin hyperpigmentation (“bronze diabetes”), hepatomegaly, diabetes, cardiomyopathy, hypogonadism, endocrine dysfunction (hypoparathyroidism, adrenal insufficiency) Data sources: [16]	Elevated serum ferritin (>300 µg/L in men, >200 µg/L in premenopausal women); elevated transferrin saturation (>45%); elevated serum Fe; genetic testing for HFE mutations (C282Y, H63D); secondary causes: elevated ferritin with normal transferrin saturation suggests inflammation/hemophagocytosis Data sources: [16]	Liver MRI with T_2_* or R_2_* for quantitative assessment of liver Fe concentration; cardiac MRI for myocardial Fe (especially in thalassemia); CT may show hepatic density increased (>75 HU) Data sources: [204]	*HFE* genotyping (C282Y, H63D) for HH; non‐*HFE* genes (*HJV*, *HAMP*, *TFR2, SLC40A1*) in atypical cases Data sources: [16]
Cu	Deficiency	Hematological: anemia (refractory to Fe), neutropenia; neurological: myelopathy, gait disturbance, paresthesia, sensory ataxia; connective tissue: skin laxity, vascular fragility (Menkes: kinky hair, neurodegeneration) Data sources: [205]	Low serum Cu (<70 µg/dL); low serum ceruloplasmin (<20 mg/dL); low 24‐h urinary Cu excretion; low serum Zn (if ZICD suspected); normal or low serum Fe with anemia Data sources: [205]	Spinal cord MRI may show T_2_ hyperintensities in dorsal columns (subacute combined degeneration‐like); brain MRI in Menkes shows cerebral and cerebellar atrophy, white matter changes, tortuous vessels Data sources: [205]	*ATP7A* for Menkes disease/occipital horn syndrome (X‐linked); *ATP7B* for Wilson's disease (differentiates from acquired deficiency) Data sources: [205]
	Overload (Wilson's disease)	Hepatic (any age): asymptomatic hepatomegaly, fatty liver, acute hepatitis, cirrhosis, acute liver failure; neurological (often second‐third decade): dysarthria, dystonia, tremor (wing‐beating), parkinsonism, ataxia; psychiatric: depression, personality change, psychosis; Kayser–Fleischer rings (Cu deposition in cornea) Data sources: [205]	Low serum Cu (paradoxically low in advanced disease due to accumulation in tissues); low serum ceruloplasmin (<20 mg/dL; <5–10 mg/dL highly suggestive); elevated 24‐h urinary Cu excretion (>100 µg/24 h); elevated hepatic Cu (>250 µg/g dry weight); slit‐lamp examination for Kayser–Fleischer rings Data sources: [205]	Brain MRI: T_1_ hyperintensity in basal ganglia (putamen, globus pallidus), midbrain “face of giant panda” sign; T_2_ hyperintensities in thalamus, brainstem; liver imaging for hepatosplenomegaly, cirrhosis, steatosis Data sources: [205]	*ATP7B* genotyping (autosomal recessive); targeted mutation analysis (e.g., H1069Q in European populations) Data sources: [205]
Zn	Deficiency	Mild: fatigue, poor wound healing, irritability; moderate‐severe: alopecia, diarrhea, dermatitis (periorificial, acral); growth retardation, delayed sexual maturation, hypogonadism, night blindness, immune dysfunction, increased infection susceptibility Data sources: [20]	Low serum Zn (<70 µg/dL, fasting morning); low serum alkaline phosphatase (adjunctive marker); hair Zn (chronic deficiency only, confounded by external contamination); measure serum albumin to correct for binding (70% bound to albumin) Data sources: [20]	Not routinely indicated; spinal cord MRI for neurological complications (ZICD: T_2_ dorsal column hyperintensities); plain radiography for bone age (pediatric growth retardation, nonspecific) Data sources: [100]	*SLC39A4* (autosomal recessive); maternal *SLC30A2*; broader panels (*ZIP8*, *ZIP13*, *ZnT9*) for complex neurodevelopmental or connective tissue syndromes Data sources: [20, 71]
	Overload/toxicity	Acute: metal fume fever (fever, chills, myalgia, metallic taste, 4–12 h postexposure to ZnO fumes); chronic: ZICD presenting with neurological deficits (gait disturbance, paresthesia, myelopathy), hematological (neutropenia, leukopenia, anemia), often insidious onset over months to years Data sources: [125]	Elevated serum Zn (>150–200 µg/dL in symptomatic patients); low serum Cu (hypocupremia); low serum ceruloplasmin; elevated urinary Zn; low ALP (Cu‐dependent); neutropenia on CBC Data sources: [125]	Chest X‐ray (metal fume fever: to exclude alternative pulmonary pathology, typically normal); spinal cord MRI (chronic ZICD myelopathy: T_2_ dorsal column hyperintensities); normal MRI does not exclude diagnosis Data sources: [100, 125]	Not indicated (no inherited forms of Zn overload); may be used to exclude other metal overload disorders (*HFE*, *ATP7B*) in differential diagnosis Data sources: [125]
Ca	Deficiency (hypocalcemia)	Neuromuscular: paresthesia (perioral, digital), muscle cramps, tetany, Chvostek's sign, Trousseau's sign, laryngospasm, seizures; prolonged QT interval; chronic: cataracts, dental abnormalities, dry skin, coarse hair Data sources: [23]	Low albumin‐corrected or ionized Ca (<8.5 mg/dL); PTH guides algorithm: low/inappropriately normal PTH (hypoparathyroidism) vs. elevated PTH (vitamin D deficiency, chronic kidney disease, pseudohypoparathyroidism); measure magnesium (hypomagnesemia causes reversible hypocalcemia); serum phosphate, creatinine, 25(OH)D Data sources: [23]	DXA (chronic hypocalcemia: assess bone mineral density); plain radiography (rickets/osteomalacia); brain MRI/CT (Fahr's syndrome: cerebral calcifications in chronic hypoparathyroidism) Data sources: [23]	*22q11.2* deletion (DiGeorge); *AIRE* (APS1); *CASR* activating mutations (autosomal dominant hypocalcemia); *GNAS* (pseudohypoparathyroidism); *HFE* (hemochromatosis with hypoparathyroidism)
	Overload (hypercalcemia)	“Bones, stones, groans, psychiatric overtones”: bone pain, nephrolithiasis, polyuria/polydipsia, constipation, fatigue, confusion, depression; severe: cardiac arrhythmias, acute kidney injury Data sources: [23]	Elevated albumin‐corrected or ionized Ca (>10.5 mg/dL); PTH guides algorithm: elevated/inappropriately normal PTH (primary hyperparathyroidism, FHH) vs. suppressed PTH (malignancy, vitamin D intoxication, granulomatous disease, milk‐alkali syndrome, thyrotoxicosis, immobilization); PTHrP, 25(OH)D, 1,25(OH)_2_D; urinary Ca/creatinine ratio (FHH: <0.01) Data sources: [23, 81]	DXA (chronic hyperparathyroidism: bone mineral density); plain radiography (subperiosteal bone resorption, osteopenia, nephrocalcinosis); MRI (brown tumors, cord compression) Data sources: [23]	*CASR* (FHH, inactivating mutations); *MEN1*, *RET*, *CDKN1B* (MEN‐associated hyperparathyroidism) Data sources: [58]
	Crystal deposition (CPPD)	Asymptomatic CPPD; OA with CPPD; acute CPP crystal arthritis (pseudogout): sudden‐onset, self‐limiting mono/oligoarthritis (knee, wrist, MCP), fever, elevated acute‐phase reactants; chronic CPP inflammatory arthritis; pseudo‐PMR; pseudoneuropathic arthropathy; crowned dens syndrome (occipital pain, neck stiffness, fever) Data sources: [206]	No specific diagnostic blood test; recommended: serum Ca, phosphate, magnesium, ferritin (hemochromatosis), ALP, TSH to exclude underlying metabolic disorders; synovial fluid aspiration: CPP crystals (weakly positively birefringent rhomboids) under polarized light microscopy Data sources: [206, 207]	X‐ray: chondrocalcinosis (punctate/linear calcifications in fibrocartilage/hyaline cartilage; sensitivity ∼40%); CT: more sensitive for deep/axial calcifications, crowned dens syndrome; DECT: discriminates CPP from BCP (>90% sensitivity); Ultrasound: high sensitivity (knee: 91%, wrist: 93.7%), OMERACT‐validated; MRI: limited for crystals, valuable for complications (cord compression) Data sources: [208]	*ANKH* (familial chondrocalcinosis, autosomal dominant, rare); not routine for sporadic age‐related CPPD Data sources: [209]

Abbreviations: ALP, alkaline phosphatase; APS1, autoimmune polyendocrine syndrome Type 1; BCP, basic Ca phosphate; CaM, calmodulin; CaMK, Ca/calmodulin‐dependent protein kinase; CASR, Ca‐sensing receptor; CBC, complete blood count; CPPD, Ca pyrophosphate deposition disease; CT, computed tomography; DECT, dual‐energy computed tomography; DXA, dual‐energy X‐ray absorptiometry; ER, endoplasmic reticulum; FHH, familial hypocalciuric hypercalcemia; HH, hereditary hemochromatosis; HU, Hounsfield units; IP3, inositol 1,4,5‐trisphosphate; MCP, metacarpophalangeal; MCU, mitochondrial Ca uniporter; MEN, multiple endocrine neoplasia; MRI, magnetic resonance imaging; OA, osteoarthritis; OMERACT, Outcome Measures in Rheumatology; PAM, peptidylglycine α‐amidating monooxygenase; PHP, pseudohypoparathyroidism; PTH, parathyroid hormone; PTHrP, PTH‐related peptide; ROS, reactive oxygen species; SERCA, sarco/endoplasmic reticulum Ca‐ATPase; SIRT, sirtuin; TCA, tricarboxylic acid; TIBC, total Fe‐binding capacity; TPO, thyroid peroxidase; ZICD, Zn‐induced Cu deficiency.

### 5.1 Diagnosis of Fe‐Associated Disorders

The diagnosis of Fe‐associated disorders requires a dual‐pronged approach that reflects the opposing ends of the Fe homeostasis spectrum: Fe deficiency and Fe overload. The diagnostic workup for Fe deficiency focuses on identifying the underlying cause of blood loss or malabsorption, whereas the evaluation of Fe overload integrates clinical assessment of end‐organ damage (e.g., hypogonadism, cardiomyopathy, diabetes mellitus), quantitative imaging like magnetic resonance imaging (MRI), and, when indicated, genetic testing for *HFE* and non‐*HFE* mutations. Accordingly, this section is structured into two subsections: Fe deficiency (Section 5.1.1) and Fe overload (Section 5.1.2), each organized by clinical assessment, biochemical testing, imaging, and genetic analysis.

#### 5.1.1 Fe Deficiency

The diagnosis of Fe deficiency requires a systematic approach integrating clinical assessment, biochemical testing, and, in selected cases, imaging and genetic analysis. Fe deficiency exists on a spectrum, progressing from depletion of Fe stores to Fe deficiency anemia, and early recognition is critical to prevent hematological and neurodevelopmental complications.

##### 5.1.1.1 Clinical Assessment

Clinical assessment of Fe deficiency begins with a thorough history to identify risk factors and characteristic manifestations. Key historical elements include menstrual blood loss in premenopausal women, gastrointestinal bleeding (from peptic ulcer disease, colorectal cancer, or angiodysplasia), inadequate dietary intake (particularly in children, adolescents, and pregnant women), malabsorption (coeliac disease, atrophic gastritis, bariatric surgery), and increased physiological demands during pregnancy and lactation [14, 84, 85]. A medication history should also be obtained, as certain drugs (e.g., proton pump inhibitors, aspirin, nonsteroidal anti‐inflammatory drugs [NSAIDs]) may contribute to gastrointestinal blood loss or impair Fe absorption.

Clinical manifestations of Fe deficiency vary with severity. Even in the absence of anemia, individuals may experience fatigue, difficulty concentrating, irritability, depression, exercise intolerance, and restless legs syndrome (affecting 32–40% of patients) [14]. As deficiency progresses to Fe deficiency anemia patients may develop pallor, glossitis, angular cheilitis, koilonychia (spoon‐shaped nails), and pica (craving for non‐nutritive substances such as ice, dirt, or starch). In children, Fe deficiency is associated with growth retardation, delayed neurodevelopment, and behavioral disturbances [14, 84, 85].

##### 5.1.1.2 Biochemical Testing

Biochemical testing is essential for confirming Fe deficiency and guiding replacement therapy. In the absence of inflammation, low serum ferritin and low transferrin saturation (TSAT) confirm the diagnosis (Table 2). However, ferritin is an acute‐phase reactant and may be falsely elevated in the setting of inflammation, infection, malignancy, or liver disease; therefore, concurrent measurement of inflammatory markers such as C‐reactive protein is recommended  [14, 84, 85].

TSAT, calculated as serum Fe divided by total Fe‐binding capacity (TIBC), reflects circulating Fe availability. A TSAT below 20% is suggestive of Fe deficiency, although this parameter exhibits diurnal variation and is influenced by recent dietary intake. Additional supportive markers include low serum Fe, elevated TIBC, and increased soluble transferrin receptor levels; the latter is less affected by inflammation and thus offers an advantage in inflammatory states. In the absence of inflammation, the combination of low ferritin and low TSAT provides high diagnostic accuracy for Fe deficiency anemia [14, 84, 85].

##### 5.1.1.3 Imaging

Imaging modalities are not routinely used for the primary diagnosis of Fe deficiency. However, in patients with suspected gastrointestinal bleeding as the underlying cause, endoscopic evaluation (esophagogastroduodenoscopy and colonoscopy) is indicated to identify bleeding lesions, particularly in older patients or those with alarm symptoms such as weight loss, hematochezia, or melena. In premenopausal women with uncomplicated Fe deficiency and no gastrointestinal symptoms, endoscopic evaluation may be deferred; however, persistent or severe deficiency warrants further investigation. Fecal occult blood testing and capsule endoscopy may be employed in select cases where conventional endoscopy is unrevealing [14].

##### 5.1.1.4 Genetic Analysis

Genetic testing is not routinely indicated for acquired Fe deficiency. Nevertheless, in patients with Fe deficiency refractory to oral Fe supplementation and no identifiable acquired cause, testing for rare inherited disorders of Fe metabolism may be considered. One such condition is Fe‐refractory Fe deficiency anemia, caused by mutations in the *TMPRSS6* gene encoding matriptase‐2, and is characterized by inappropriately high hepcidin levels, impaired Fe absorption, and a poor response to oral Fe therapy. Additionally, genetic testing for coeliac disease (*HLA‐DQ2/DQ8*) or other malabsorptive disorders may be pursued in selected cases [14].

#### 5.1.2 Fe Overload

The diagnosis of Fe overload requires a multifaceted approach integrating clinical assessment, biochemical testing, imaging, and genetic analysis.

##### 5.1.2.1 Clinical Assessment

Clinical assessment serves as the foundation for diagnosing Fe overload and its associated endocrine complications. In males, the diagnostic evaluation of hypogonadism should begin with a comprehensive clinical history, encompassing hematological diagnosis (e.g., thalassemia, HH), transfusion burden, serum ferritin levels, and the regimen of Fe chelation therapy (ICT). This should be followed by an andrological physical examination to identify risk factors for male infertility and to detect clinical signs and symptoms of hypogonadism, including reduced libido, erectile dysfunction, fatigue, decreased muscle mass, and infertility [210].

In females, assessment of menstrual history, including age at menarche, cycle regularity, and the presence of primary or secondary amenorrhea, is equally essential. Clinical manifestations of hypogonadism in women may include oligomenorrhea, amenorrhea, infertility, vasomotor symptoms (hot flushes), and vaginal dryness [211, 212]. A thorough medication history should also be obtained, as certain Fe chelators may have differing effects on endocrine function.

Beyond the reproductive axis, clinical evaluation should assess for other endocrine sequelae of Fe overload, including growth hormone deficiency (manifesting as short stature or reduced growth velocity in children and adolescents), hypothyroidism (fatigue, cold intolerance, weight gain), and hypoparathyroidism (paresthesia, muscle cramps, tetany). Bone pain or fragility fractures should prompt consideration of osteoporosis secondary to hypogonadism and direct Fe toxicity on bone metabolism [213, 214].

##### 5.1.2.2 Biochemical Testing

Biochemical testing is essential for confirming endocrine dysfunction and guiding replacement therapy. In males, proactive monitoring of serum testosterone levels is recommended in patients with Fe overload disorders, such as transfusion‐dependent thalassemia, even before overt symptoms of hypogonadism emerge [210]. Low serum testosterone, measured alongside gonadotropin levels to distinguish central from primary hypogonadism, guides hormone replacement therapy [215] (Table 2). The European Academy of Andrology further emphasized the importance of calculated free testosterone as a critical diagnostic parameter, particularly in men with total testosterone in the “grey zone” (8–12 nmol/L) or those with abnormal sex hormone‐binding globulin (SHBG) levels [215]. Measurement of SHBG is especially relevant in Fe overload disorders, as its levels are frequently elevated secondary to liver damage [211, 212, 216, 217]. Additionally, hormonal profiling should include serum follicle‐stimulating hormone (FSH) and luteinizing hormone (LH) to distinguish hypogonadotropic (central) from hypergonadotropic (primary gonadal) hypogonadism. In females, endocrine assessments should ideally be conducted during the early follicular phase (Days 2–5 of the menstrual cycle) and include serum FSH, LH, estradiol, prolactin, and anti‐Müllerian hormone (AMH) concentrations. AMH serves as a reliable biomarker of ovarian reserve and is particularly useful in assessing gonadotoxicity in women with Fe overload disorders [211, 212, 216].

Beyond reproductive hormones, biochemical evaluation should include serum Ca, phosphate, and PTH to exclude hypoparathyroidism, as well as thyroid function tests (TSH; free T_4_) to assess for hypothyroidism [218]. Serum ferritin remains the primary marker of Fe burden, although it must be interpreted in the context of inflammation or liver disease [219]. Transferrin saturation is also a useful indicator of Fe overload, with values consistently exceeding 45% suggestive of HH [220].

##### 5.1.2.3 Imaging

Imaging plays a critical role in detecting Fe deposition in endocrine organs and predicting functional impairment. Quantitative MRI (T_2_*) is the reference standard for noninvasive assessment of hepatic, cardiac, and pituitary Fe burden; and its values correlate with endocrine dysfunction severity and guide chelation intensity [204, 213, 221, 222, 223, 224, 225, 226, 227] (Table 2).

Bone mineral density assessment via dual‐energy X‐ray absorptiometry is strongly recommended in hypogonadal patients of any age, as Fe‐mediated oxidative stress and androgen/estrogen deficiency synergistically impair bone remodeling, thereby increasing fracture risk [213, 214]. Hypogonadism during critical periods of bone accretion, such as adolescence and young adulthood, can compromise the achievement of peak bone mass, predisposing individuals to premature osteopenia and osteoporosis [228]. In adults, sustained sex hormone deficiency accelerates bone resorption relative to formation, leading to cortical thinning and trabecular bone loss, particularly at the lumbar spine and femoral neck [229]. Patients with Fe overload disorders often present with multiple additional risk factors for bone fragility, including growth hormone deficiency, chronic liver disease, vitamin D insufficiency, and the direct toxic effects of Fe on osteoblasts [230]. Therefore, routine dual‐energy X‐ray absorptiometry monitoring is essential. Baseline assessment should be performed at the diagnosis of hypogonadism or at the onset of puberty, with follow‐up scans every 1–2 years, depending on clinical risk factors and initial *Z*‐scores [231].

##### 5.1.2.4 Genetic Analysis

When Fe overload is identified without an obvious acquired cause (e.g., multiple transfusions or chronic hemolysis), genetic testing should be considered to identify hereditary forms of Fe overload. Genetic testing for *HFE* is first‐line in suspected HH [232, 233]; if negative in young patients with severe overload, non‐*HFE* genes should be pursued at referral centers [234, 235, 236, 237, 238, 239, 240, 241, 242, 243] (Table 2).

Genetic testing is particularly important in younger patients presenting with hypogonadotropic hypogonadism, cardiomyopathy, diabetes mellitus, or chronic liver disease of uncertain etiology, as early diagnosis enables timely intervention to prevent irreversible organ damage [244]. The diagnostic approach typically begins with first‐level testing for *HFE* C282Y and H63D mutations, which are widely available [245, 246]. If no *HFE* mutation was identified but clinical suspicion remained high, particularly in young patients, those with severe Fe overload, or those with a family history suggestive of juvenile hemochromatosis, second‐level genetic testing using next‐generation sequencing panels targeting non‐*HFE* genes (*HJV*, *HAMP*, *TFR2*, *SLC40A1*) should be pursued at referral centers with expertise in Fe disorders [247]. Interpretation of non‐*HFE* variants may require bioinformatics analysis and expert review, as some variants may be classified as variants of uncertain significance [233].

Early genetic diagnosis enables targeted therapeutic intervention, primarily phlebotomy (venesection) as first‐line therapy to reduce Fe burden, or ICT (e.g., deferasirox, deferoxamine) for patients who cannot tolerate phlebotomy due to anemia or poor venous access [234, 235, 236, 237, 238, 248]. In patients with end‐stage liver disease due to hemochromatosis, liver transplantation may be required. Importantly, early initiation of Fe depletion therapy before the development of cirrhosis, cardiomyopathy, or permanent hypogonadism may significantly improve long‐term outcomes and the quality of life [244, 248].

### 5.2 Diagnosis of Cu‐Associated Disorders

The diagnosis of Cu‐associated disorders requires a multifaceted approach integrating clinical assessment, biochemical testing, imaging, and genetic analysis. These disorders span a spectrum from Cu deficiency (acquired or inherited) to Cu overload, with Wilson's disease being the most well‐characterized overload disorder and Menkes disease representing a severe inherited deficiency syndrome [205, 249]. The diagnostic approach differs largely between Cu deficiency and Cu overload, each with distinct risk factors, clinical presentations, biochemical markers, and genetic underpinnings. Acquired Cu deficiency, often resulting from malabsorption, excessive Zn ingestion, or malnutrition, presents with hematological and neurological manifestations that may become irreversible if treatment is delayed, whereas inherited Cu deficiency (Menkes disease) manifests in early infancy with severe neurological degeneration and connective tissue abnormalities. In contrast, Cu overload is dominated by Wilson's disease, an autosomal recessive disorder caused by *ATP7B* mutations, which leads to pathological Cu accumulation in the liver and brain, with protean hepatic, neurological, and psychiatric presentations. Accordingly, this section is structured into two subsections: Cu deficiency (Section 5.2.1) and Cu overload (Section 5.2.2), with the latter further distinguished by the availability of quantitative biochemical and genetic diagnostic tools, including serum ceruloplasmin, 24‐h urinary Cu excretion, hepatic Cu quantification, and ATP7B mutational analysis.

#### 5.2.1 Cu Deficiency

The diagnostic workup for suspected Cu deficiency integrates clinical assessment, biochemical testing, and, when indicated, imaging and genetic analysis.

##### 5.2.1.1 Clinical Assessment

Clinical assessment of suspected Cu deficiency begins with a thorough history to identify risk factors, including malabsorption following gastric surgery (e.g., Roux‐en‐Y gastric bypass, gastrectomy), excessive Zn ingestion (from over‐the‐counter supplements or denture adhesives), malnutrition, prolonged total parenteral nutrition without adequate Cu supplementation, enteropathies with protein‐losing enteropathy, and chronic kidney disease treated with high‐dose Zn  [98, 99]. Physical examination may reveal signs of hematological abnormalities (pallor, fatigue) and neurological deficits (sensory ataxia, gait disturbances, paresthesia, and spasticity). Cu deficiency myelopathy, also termed Cu deficiency‐associated myeloneuropathy, closely resembles subacute combined degeneration seen in vitamin B_12_ deficiency and may present with proprioceptive deficits and spasticity [100].

##### 5.2.1.2 Biochemical Testing

Low serum Cu and low serum ceruloplasmin confirm the diagnosis of Cu deficiency [18, 100, 101]; concurrent serum Zn measurement is essential to rule out ZICD [98, 99, 128, 250] (Table 2). A complete blood count may reveal anemia (typically microcytic, hypochromic, or normocytic, often refractory to Fe supplementation), as well as neutropenia or pancytopenia in severe cases [98, 99, 128, 250]. Low serum ALP activity may also be observed, as ALP is a Cu‐dependent enzyme, though this finding is nonspecific [72].

##### 5.2.1.3 Imaging

In patients presenting with myelopathy, spinal cord MRI may show dorsal column T_2_ hyperintensities, resembling the findings seen in subacute combined degeneration of the spinal cord. Nevertheless, a normal MRI study does not exclude the diagnosis of Cu deficiency myelopathy [100]. Imaging is directed at assessing neurological sequelae rather than establishing the primary diagnosis of Cu deficiency.

##### 5.2.1.4 Genetic Analysis

Genetic testing for *ATP7A* should be reserved for suspected inherited disorders, particularly in young patients presenting with early‐onset neurological symptoms, kinky or brittle hair, and connective tissue abnormalities characteristic of Menkes disease or occipital horn syndrome [205]. For the majority of adult patients with acquired Cu deficiency, genetic testing is not indicated. Early diagnosis is critical because hematological abnormalities often respond well to Cu repletion, whereas neurological deficits may become irreversible if treatment was delayed [98, 99].

#### 5.2.2 Cu Overload

##### 5.2.2.1 Clinical Assessment

Clinical assessment focuses on identifying organ‐specific manifestations of Cu accumulation such as Kayser–Fleischer rings, a golden‐brown ring surrounding the iris caused by Cu deposition in the Descemet's membrane though they may be absent in up to 50% of patients with predominantly hepatic involvement [205, 251]. Neurological manifestations include movement disorders such as tremors, dystonia (involuntary muscular spasms), dysarthria (difficulties speaking), and ataxia, as well as psychiatric disturbances including anxiety, depression, personality changes, and cognitive impairments [252, 253]. Hepatic presentations range from asymptomatic hepatomegaly and isolated transaminitis to acute hepatitis, cirrhosis, and acute liver failure.

##### 5.2.2.2 Biochemical Testing

Serum ceruloplasmin concentration is a key initial test for Wilson's disease, levels of which are typically low due to impaired Cu binding [205]. However, ceruloplasmin is an acute‐phase reactant and may be normal in a minority of patients, particularly those with active inflammation or concurrent liver disease. Elevated 24‐h urinary Cu and, when indicated, hepatic Cu quantification confirm the diagnosis of Wilson's disease [205] (Table 2). In addition, relative exchangeable Cu has emerged as a valuable diagnostic parameter, offering improved sensitivity and specificity compared with traditional markers [254, 255].

##### 5.2.2.3 Imaging

Imaging techniques play a supportive but nondiagnostic role in Cu‐associated disorders. Brain MRI reveals characteristic basal ganglia and brainstem abnormalities in Wilson's disease [256] (Table 2). In advanced stages, atrophy of these structures may be observed [257]. While MRI findings are highly suggestive in the appropriate clinical context, they are not specific to Wilson's disease and may overlap with other metabolic or neurodegenerative disorders [258]. Abdominal imaging, including ultrasound or MRI, can assess liver parenchyma for signs of cirrhosis, steatosis, or hepatomegaly, and may detect splenomegaly secondary to portal hypertension [259]. Unlike Fe overload, for which T_2_* MRI provides quantitative assessment of tissue Fe deposition, no equivalent noninvasive imaging modality exists for quantitative Cu measurement in the liver or brain, making biopsy and biochemical analysis the current gold standard [260, 261].

##### 5.2.2.4 Genetic Analysis

Genetic analysis plays a definitive role in the diagnosis of inherited Cu metabolism disorders. *ATP7B* testing confirms Wilson's disease [262, 263], whereas *ATP7A* testing is reserved for suspected inherited Cu deficiency [205, 264, 265, 266] (Table 2).

### 5.3 Diagnosis of Zn‐Associated Disorders

The clinical relevance of Zn first entered modern medical recognition through the seminal observations of Prasad in 1963, who identified severe dietary Zn deficiency and thereby established Zn as an essential trace element required for normal human growth, immune competence, neurodevelopment, and sexual maturation [20]. Six decades later, the spectrum of Zn‐associated disorders has expanded to encompass not only acquired and inherited deficiency syndromes but also chronic toxicity states, most notably ZICD resulting from excessive supplementation or occupational exposure. Accurate diagnosis of these conditions requires a systematic approach integrating clinical assessment, biochemical testing, and, in selected cases, imaging and genetic analysis. The diagnostic approach differs substantially between Zn deficiency and Zn toxicity, each with its own set of risk factors, clinical presentations, biochemical thresholds, and interpretive considerations. Accordingly, this section is structured into two subsections: Zn deficiency (Section 5.3.1) and Zn overload (Section 5.3.2), with the latter further distinguished by the absence of inherited forms, unlike Fe and Cu overload disorders, and the critical need to identify exogenous sources of excess Zn.

#### 5.3.1 Zn Deficiency

The diagnosis of Zn deficiency requires a systematic approach integrating clinical assessment, biochemical testing, and, in selected cases, genetic analysis. The heterogeneous presentation of Zn deficiency, ranging from mild, nonspecific symptoms to severe dermatological and neurological manifestations, necessitates a low threshold for laboratory evaluation in at‐risk populations.

##### 5.3.1.1 Clinical Assessment

Clinical assessment begins with a thorough history and physical examination to identify risk factors and characteristic manifestations of Zn deficiency. Key historical elements include dietary intake (particularly cereal‐based diets high in phytates), gastrointestinal disorders affecting absorption (coeliac disease, Crohn's disease, bariatric surgery), increased physiological demands (pregnancy, lactation, rapid growth in children), and excessive losses (chronic diarrhea, burns, hemodialysis) [70]. A medication history should also be obtained, as certain drugs (e.g., proton pump inhibitors, histamine H_2_ receptor antagonists, and some Fe chelators) may impair Zn absorption [70].

Physical examination findings in Zn deficiency vary with severity. Mild deficiency may present with nonspecific symptoms including fatigue, irritability, and poor wound healing. Moderate to severe deficiency manifests with the classic triad of alopecia, diarrhea, and dermatitis, particularly in acrodermatitis enteropathica. Dermatological findings typically involve periorificial (perioral, perianal) and acral (hands, feet) areas, presenting as erythematous, scaly, or vesiculobullous lesions. Additional features include growth retardation in children, delayed sexual maturation, hypogonadism, night blindness (due to impaired vitamin A metabolism), and increased susceptibility to infections resulting from immune dysfunction [19, 20].

In patients presenting with neurological symptoms such as gait disturbances, paresthesia or myelopathy, a detailed neurological examination is essential to assess for complications of chronic Zn deficiency or ZICD, which may develop insidiously over months to years [127, 129].

##### 5.3.1.2 Biochemical Testing

Serum Zn concentration remains the most widely used and practical laboratory marker for assessing Zn status. Fasting serum Zn is the primary biomarker, though it must be interpreted with concurrent serum albumin (Table 2). However, reference ranges may vary between laboratories and must be interpreted in the context of age, sex, and diurnal variation, as Zn levels fluctuate in response to meals and circadian rhythms [20, 103].

Important preanalytical and analytical considerations significantly influence the accuracy of Zn measurements. Since approximately 70% of circulating Zn is bound to albumin, concurrent measurement of serum albumin levels should be measured for accurate interpretation of Zn results [20]. Hypoalbuminemia may produce falsely low Zn values even when functional Zn status is normal [20]. To optimize the diagnostic accuracy, fasting morning samples should be collected, plasma or serum should be separated from cells within 45 min of collection (as prolonged contact with red blood cells leads to Zn uptake and falsely low readings), and Zn‐free vacuum tubes should be used to avoid exogenous contamination [20, 103].

Low serum ALP activity serves as a valuable adjunctive marker of functional Zn deficiency. ALP is a Zn‐dependent enzyme whose activity declines in the setting of inadequate Zn availability [267]. Therefore, an unexplained low ALP level in a patient with compatible symptoms should prompt consideration of Zn deficiency, even if serum Zn levels were at the borderline levels [72].

Other biomarkers have limited utility in routine clinical practice. Hair Zn levels may be useful only in the context of chronic deficiency, reflecting long‐term Zn status over weeks to months rather than acute fluctuations [268]. However, hair Zn analysis is confounded by external contamination from hair products and environmental exposure [269]. Urinary Zn is not considered a reliable parameter for assessing Zn deficiency due to high intra‐individual variability and dependence on renal function, dietary intake, and metabolic status [20, 103].

##### 5.3.1.3 Imaging

Imaging modalities are not routinely used in the diagnosis of Zn deficiency itself. However, in patients presenting with neurological complications, particularly those with suspected ZICD or chronic, severe Zn deficiency, spinal cord MRI may be indicated to evaluate for myelopathy [270]. In such cases, MRI may demonstrate T_2_ hyperintensities in the dorsal columns, resembling the findings observed in subacute combined degeneration of the spinal cord (classically associated with vitamin B_12_ deficiency) [270]. It is important to note that a normal MRI study does not exclude the diagnosis of Zn deficiency‐related myelopathy [100].

In pediatric patients with growth retardation or delayed bone age, plain radiography of the left hand and wrist may be performed to assess skeletal maturation; however, this finding is nonspecific and should not replace biochemical confirmation of Zn status [271].

##### 5.3.1.4 Genetic Analysis

Genetic testing is indicated when inherited disorders of Zn metabolism are suspected, particularly in patients presenting with early‐onset, severe Zn deficiency that is refractory to standard dietary management or in those with a family history of consanguinity [272]. *SLC39A4* testing confirms acrodermatitis enteropathica [70, 71]; maternal *SLC30A2* testing identifies TNZD [61] (Table 2). Beyond these classic disorders, mutations in other Zn transporters, including* ZIP13* and *ZnT9*, have been associated with distinct clinical syndromes (e.g., intellectual disability, Ehlers–Danlos‐like phenotypes, Birk–Landau–Perez syndrome) [273, 274]. In patients with complex neurodevelopmental or connective tissue disorders and concurrent biochemical evidence of Zn dyshomeostasis, broader genetic testing using next‐generation sequencing panels targeting metal transporter genes may be considered [9, 55, 56].

#### 5.3.2 Zn Overload

The diagnosis of Zn overload (Zn toxicity) requires a distinct approach from that of Zn deficiency, as the clinical presentation, biochemical targets, and interpretive thresholds differ substantially. Unlike Fe and Cu overload disorders, which have well‐characterized inherited forms, all reported cases of Zn toxicity are acquired, making the identification of exogenous Zn sources a critical diagnostic step [103, 104, 105].

##### 5.3.2.1 Clinical Assessment

Clinical assessment forms the foundation of diagnosing Zn toxicity, with emphasis on identifying the mode of overload and recognizing the insidious onset of complications [125]. Most reports of Zn toxicity originate from either experimental animal studies employing extreme supraphysiological doses or isolated case reports involving inappropriate chronic exposure, such as excessive denture cream use or misguided self‐administration of very high supplemental doses [125]. A thorough history is essential to identify the source of Zn excess, which may include occupational exposure (welding or galvanizing fumes), overuse of Zn‐containing supplements, or use of Zn‐containing denture adhesives [103, 104, 105, 123, 124, 125]. The US Institute of Medicine has established a tolerable upper intake level of 40 mg/day for Zn in adults (≥19 years). Intakes exceeding 200 mg/day are classified as acute toxicity, while chronic toxicity is associated with prolonged daily intakes of 50–150 mg/day [103, 104, 105, 125].

Physical examination may reveal signs of secondary Cu deficiency in cases of chronic Zn toxicity, including neurological deficits such as gait disturbances, paresthesia, and myelopathy, which develop insidiously over months to years [127, 129]. Metal fume fever, an acute condition resulting from occupational inhalation of Zn oxide fumes, presents with flu‐like symptoms including fever, chills, myalgia, headache, and a metallic taste in the mouth, typically occurring 4–12 h after exposure [275]. The diagnosis of metal fume fever is primarily clinical, based on history and symptom resolution after removal from exposure, though chest X‐ray may be indicated to exclude alternative pulmonary pathology [124, 125, 276].

##### 5.3.2.2 Biochemical Testing

Biochemical testing provides confirmatory evidence of Zn toxicity. Serum Zn concentration remains the primary laboratory marker, with levels typically exceeding 150 µg/dL in symptomatic patients being suggestive of Zn overload [124]. As detailed in Section 5.3.1, interpretation of serum Zn levels requires attention to preanalytical factors (fasting morning sampling, prompt plasma/serum separation, and Zn‐free collection tubes) and concurrent albumin measurement [20, 103, 104, 105].

The most clinically significant complication of chronic Zn toxicity is ZICD, the biochemical hallmark of which is the coexistence of hyperzincemia and hypocupremia [127]. Confirmatory testing for ZICD includes measurement of serum ceruloplasmin, which is typically low (below 20 mg/dL), and urinary Zn excretion, which is usually elevated due to increased systemic Zn load [127, 128]. A complete blood count may reveal hematological manifestations of ZICD, including neutropenia (the most common finding), leukopenia, and anemia, which often develop insidiously over months to years and lead to delayed diagnosis [127, 129]. Serum ALP activity may also be reduced in ZICD, as ALP is a Cu‐dependent enzyme; however, this finding is nonspecific and should be interpreted in conjunction with serum Cu and Zn levels [72].

Urinary Zn, while not useful for diagnosing Zn deficiency owing to high intra‐individual variability and dependence on renal function, dietary intake, and metabolic status, plays an important role in ZICD diagnosis, as levels are typically elevated in this condition [20, 103, 104, 105, 128].

##### 5.3.2.3 Imaging

Imaging modalities are not routinely used for the primary diagnosis of Zn toxicity but play a supportive role in specific clinical contexts. Chest X‐ray is indicated in cases of suspected metal fume fever to exclude alternative pulmonary pathology such as pneumonia or other occupational lung diseases. However, the diagnosis remains primarily clinical based on history and temporal relationship to exposure, with imaging findings typically normal in uncomplicated cases [124, 125, 276]. In patients presenting with neurological complications of chronic ZICD, particularly myelopathy, gait disturbances, or sensory ataxia, spinal cord MRI may be employed to evaluate for structural abnormalities [270]. In such cases, MRI may demonstrate T_2_ hyperintensities in the dorsal columns, resembling the findings observed in subacute combined degeneration of the spinal cord classically associated with vitamin B_12_ deficiency [270]. However, it is important to note that a normal MRI study does not exclude the diagnosis of ZICD‐related myelopathy, and such an imaging is directed at assessing sequelae rather than establishing the diagnosis of Zn dyshomeostasis itself [100].

##### 5.3.2.4 Genetic Analysis

Genetic analysis has a limited role in the diagnosis of Zn overload, as no inherited conditions of Zn toxicity due to mutations in Zn transporters have been reported up to date. Unlike Fe overload (HH) and Cu overload (Wilson's disease), all cases of Zn toxicity are acquired, and genetic testing is therefore not indicated for the diagnosis of Zn overload per se [103, 104, 105]. However, genetic testing may be relevant in the differential diagnosis of Zn‐associated disorders in specific scenarios, such as excluding other inherited metal overload disorders (*HFE* for Fe overload, *ATP7B* for Wilson's disease) in patients presenting with features suggestive of metal accumulation but without clear acquired risk factors. In patients with complex neurodevelopmental phenotypes and concurrent abnormalities in Zn homeostasis, testing for rare genetic syndromes affecting metal transporters (e.g., *SLC30A9* mutations in Birk–Landau–Perez syndrome) may be considered, though these are primarily disorders of Zn deficiency or mislocalization rather than true overload [9, 56]. In the context of suspected ZICD, genetic testing for *ATP7A* (Menkes disease, occipital horn syndrome) is not indicated unless inherited Cu deficiency is suspected based on early‐onset neurological symptoms, kinky hair, or connective tissue abnormalities in a young patient [205].

### 5.4 Diagnosis of Ca‐Associated Disorders

Ca‐associated disorders encompass a broad spectrum of conditions characterized by either dysregulation of systemic Ca homeostasis (hypercalcemia or hypocalcemia) or abnormal deposition of Ca‐containing crystals in articular and periarticular tissues (e.g., Ca pyrophosphate deposition disease, CPPD). The diagnostic approach therefore varies according to the clinical presentation but generally integrates clinical assessment, biochemical testing, imaging, and, in selected cases, genetic analysis. Accordingly, this section is structured into three subsections: Ca deficiency (hypocalcemia) (Section 5.4.1), Ca overload (hypercalcemia) (Section 5.4.2), and CPPD (Section 5.4.3), each organized by clinical assessment, biochemical testing, imaging, and genetic analysis where applicable.

#### 5.4.1 Ca Deficiency (Hypocalcemia)

##### 5.4.1.1 Clinical Assessment

Hypocalcemia typically results from inadequate dietary Ca intake, vitamin D deficiency, PTH deficiency or resistance, or chronic kidney disease. Clinical assessment begins with a thorough history to identify risk factors, including thyroid or parathyroid surgery (postsurgical hypoparathyroidism), use of Ca‐chelating agents (e.g., citrate in blood transfusions), bisphosphonates, denosumab, or calcitonin [81, 277].

Symptoms of hypocalcemia are primarily neuromuscular, including paresthesia (perioral and digital), muscle cramps, tetany, Chvostek's sign (facial muscle twitching upon tapping the facial nerve), Trousseau's sign (carpopedal spasm following ischemic maneuver), and, in severe cases, laryngospasm, seizures, and prolonged QT interval predisposing to ventricular arrhythmias [80]. Chronic hypocalcemia may present with cataracts, dental abnormalities, dry skin, and coarse hair [83].

##### 5.4.1.2 Biochemical Testing

Biochemical testing is the cornerstone of diagnosing hypocalcemia. The diagnostic algorithm for hypocalcemia is PTH‐guided: low/undetectable PTH indicates hypoparathyroidism, while elevated PTH points to vitamin D deficiency, chronic kidney disease, or PHP [81, 278, 279, 280, 281, 282, 283, 284]. Serum magnesium must always be measured, as hypomagnesemia represents a reversible cause of hypocalcemia [82] (Table 2).

##### 5.4.1.3 Imaging

Imaging plays a supportive role in Ca deficiency disorders. Dual‐energy X‐ray absorptiometry is indicated in patients with chronic hypocalcemia to assess bone mineral density, as chronic hypocalcemia is associated with accelerated bone loss and increased fracture risk [208]. Plain radiography may be used to assess for rickets in children or osteomalacia in adults [285]. In patients with chronic hypoparathyroidism, brain MRI or computed tomography (CT) may reveal cerebral calcifications (Fahr's syndrome) [286].

##### 5.4.1.4 Genetic Analysis

Genetic testing is indicated in young patients with hypoparathyroidism or those with syndromic features [287]. DiGeorge syndrome (22q11.2 deletion) presents with hypocalcemia due to parathyroid agenesis, often accompanied by cardiac defects and characteristic facies [139]. Autoimmune polyendocrine syndrome Type 1 (APS1), caused by* AIRE* gene mutations, manifests with hypoparathyroidism, adrenal insufficiency, and chronic mucocutaneous candidiasis [140]. Activating mutations of *CASR *cause autosomal dominant hypocalcemia (ADH), characterized by mild hypocalcemia with inappropriately low PTH and often hypomagnesemia [141]. PHP, typically due to *GNAS *mutations (maternally inherited), presents with PTH resistance, hypocalcemia, hyperphosphatemia, and elevated PTH, often accompanied by Albright's hereditary osteodystrophy (short stature, round facies, brachydactyly, subcutaneous ossifications) [58].

Additionally, in patients with HH, which can cause hypoparathyroidism due to Fe deposition in the parathyroid glands, *HFE* gene testing (C282Y, H63D) is recommended in those with unexplained Fe overload, particularly when accompanied by endocrine dysfunction including hypoparathyroidism [288].

#### 5.4.2 Ca Overload (Hypercalcemia)

##### 5.4.2.1 Clinical Assessment

Hypercalcemia typically results from primary hyperparathyroidism, malignancy, vitamin D intoxication, or granulomatous diseases [289]. Clinical assessment begins with a thorough history to identify risk factors, including thiazide diuretics, lithium, excessive vitamin D or Ca supplementation, and milk‐alkali syndrome [290, 291].

Clinical manifestations of hypercalcemia range from mild, nonspecific symptoms (fatigue, constipation, polyuria, polydipsia) to severe complications including nephrolithiasis, acute kidney injury, neuropsychiatric disturbances (confusion, depression, lethargy), and, in extreme cases, cardiac arrhythmias [206, 292, 293]. The classic mnemonic ‘bones, stones, groans, psychiatric overtones’ remains clinically useful for recalling the classical features of hypercalcemia [80].

##### 5.4.2.2 Biochemical Testing

Biochemical testing is the cornerstone of diagnosing hypercalcemia. The initial laboratory evaluation should include measurement of serum Ca (preferably albumin‐corrected or ionized Ca), serum phosphate, magnesium, creatinine, and intact PTH [278, 279, 280, 281].

In hypercalcemia, the differential diagnosis is guided by the PTH level. PTH‐mediated hypercalcemia is most commonly caused by primary hyperparathyroidism (elevated or inappropriately normal PTH), which may be sporadic or familial (e.g., multiple endocrine neoplasia [MEN] Types 1, 2A, and 4; FHH) [294, 295]. PTH‐independent hypercalcemia (suppressed PTH) is typically caused by malignancy (e.g., squamous cell carcinoma, breast cancer, myeloma, lymphoma) via PTH‐related peptide (PTHrP) production, vitamin D intoxication, granulomatous diseases (sarcoidosis, tuberculosis) with increased 1,25‐dihydroxyvitamin D production, thyrotoxicosis, adrenal insufficiency, immobilization, or milk‐alkali syndrome [289, 296, 297, 298]. Measurement of PTHrP, 25‐hydroxyvitamin D, and 1,25‐dihydroxyvitamin D helps localize the etiology [299]. Urinary Ca excretion (urinary Ca‐to‐creatinine ratio or 24‐h urinary Ca) distinguishes primary hyperparathyroidism (normal or elevated excretion) from FHH (low excretion, typically <0.01) [295].

##### 5.4.2.3 Imaging

Imaging plays a supportive role in Ca overload disorders. Dual‐energy X‐ray absorptiometry is indicated in patients with chronic hyperparathyroidism to assess bone mineral density, as hyperparathyroidism is associated with accelerated bone loss and increased fracture risk [296]. Plain radiography may show subperiosteal bone resorption (classically in primary hyperparathyroidism), osteopenia, or nephrocalcinosis [300]. In patients with suspected brown tumors or spinal cord compression, MRI is valuable for assessing complications [286].

##### 5.4.2.4 Genetic Analysis

Genetic testing is indicated in specific clinical contexts. In FHH, caused by inactivating mutations in *CASR*, genetic testing can be used to confirm the diagnosis and distinguish FHH from primary hyperparathyroidism, thereby avoiding unnecessary parathyroid surgery [301, 302, 303]. FHH presents with lifelong mild asymptomatic hypercalcemia, hypocalciuria (urinary Ca‐to‐creatinine clearance ratio <0.01), and normal or mildly elevated PTH [295].

In primary hyperparathyroidism, genetic testing is recommended when hereditary syndromes are suspected. MEN Types 1, 2A, and 4 are associated with hyperparathyroidism; and testing for *MEN1*, *RET*, or *CDKN1B *mutations guides surveillance for other endocrine tumors (like pituitary, pancreatic, medullary thyroid, pheochromocytoma) [209, 304].

#### 5.4.3 Ca Overload (CPPD)

##### 5.4.3.1 Clinical Assessment

In patients with CPPD, clinical features vary according to the specific phenotype. The European Alliance of Associations for Rheumatology classifies CPPD into four main phenotypes: (1) asymptomatic CPPD; (2) osteoarthritis with CPPD; (3) acute CPP crystal arthritis (pseudogout); and (4) chronic CPP inflammatory crystal arthritis [207, 305]. Additional variants include pseudo‐polymyalgia rheumatica (pseudo‐PMR), pseudoneuropathic arthropathy, and tumoural CPPD [306]. The acute presentation of pseudogout is characterized by sudden‐onset, self‐limiting episodes of joint pain, swelling, erythema, and tenderness, most commonly affecting the knee, wrist, or metacarpophalangeal joints, and is often accompanied by fever and elevated acute‐phase reactants [307].

A detailed medication history is essential, as certain drugs may induce or exacerbate crystal deposition. Investigations for underlying metabolic disorders have been reported, particularly among younger patients (<60 years) or those with recurrent or severe diseases [306, 308].

##### 5.4.3.2 Biochemical Testing

CPPD has no specific diagnostic blood test. However, recommended laboratory evaluation includes serum Ca, phosphate, magnesium, ferritin (for hemochromatosis), ALP, and thyroid‐stimulating hormone to exclude underlying metabolic disorders [306, 308].

##### 5.4.3.3 Imaging

Imaging modalities are essential for detecting Ca crystal deposition and guiding therapy, including radiography (X‐ray), CT, MRI, and ultrasound. CPPD diagnosis relies on imaging detection of crystal deposits: X‐ray shows chondrocalcinosis, though with a sensitivity approximately 40% [308, 309, 310]; CT is valuable for axial involvement [311, 312]; and ultrasound offers superior sensitivity [313, 314, 315, 316, 317], particularly at the knee and wrist [315, 318] (Table 2). MRI is limited in the primary diagnosis of CPPD yet is valuable in assessing complications such as spinal cord compressions [319].

##### 5.4.3.4 Genetic Analysis

Genetic testing is not a routine practice for sporadic, age‐related CPPD. However, familial chondrocalcinosis has been associated with mutations in the *ANKH* gene (encoding the progressive ankylosis protein), which regulates inorganic pyrophosphate transport [320, 321]. These rare autosomal dominant conditions present with early‐onset, often severe, CPPD and may benefit from genetic counseling [321].

### 5.5 Summary

In summary, the diagnosis of metal‐related disorders integrates clinical assessment, biochemical testing, imaging, and genetic analysis, with the specific algorithm varying according to the metal and the type of imbalance. Early and accurate diagnosis is critical, as neurological, endocrine, and organ‐specific damage may become irreversible if the treatment was delayed. For Fe, the combination of ferritin, transferrin saturation, T_2_* MRI, and *HFE* genotyping enables precise characterization of deficiency or overload. Cu diagnosis relies on serum Cu, ceruloplasmin, urinary Cu excretion, and *ATP7A/ATP7B* testing to differentiate acquired deficiency from Wilson's or Menkes disease. Zn disorders are distinguished by serum Zn levels, with deficiency marked by values below 70 µg/dL and toxicity by hyperzincemia with secondary hypocupremia, though no inherited form of Zn overload exists. Ca diagnosis requires PTH‐guided evaluation of hypocalcemia or hypercalcemia, while CPPD depends on imaging detection of crystal deposits. Once the diagnosis is established, whether deficiency, overload, or crystal deposition, the next critical step is the implementation of targeted therapeutic strategies, which form the focus of the next section.

## 6 Therapeutic Strategies Targeting Metal Ions

Building upon the diagnostic frameworks established in Section 5, this section transitions from disease identification to therapeutic intervention. The management of metal‐related disorders has evolved considerably over recent decades, moving from purely supportive measures to targeted strategies that address the underlying metal imbalance, whether deficiency, overload, or mislocalization, and its downstream molecular consequences. Mirroring the structure of the previous sections, this section addresses each metal in turn, covering mainstream treatments and supportive therapeutic avenues.

### 6.1 Therapeutics Targeting Fe

The management of Fe overload disorders requires a multifaceted therapeutic approach tailored to the underlying etiology, severity of Fe burden, and presence of end‐organ complications. This section provides a comprehensive overview of current and emerging therapeutic strategies for Fe overload disorders, from first‐line phlebotomy and chelation to hormone replacement and experimental ferroptosis‐targeted interventions.

#### 6.1.1 Fe Chelation Therapy

ICT represents the cornerstone of treatment for systemic Fe overload, aimed at reducing the systemic Fe burden and preventing endocrine complications when initiated early [322, 323, 324]. Several chelation agents are available, including deferoxamine, deferasirox, and deferiprone. In a large cohort of 713 patients undergoing ICT, the prevalence of hypogonadism differed depending on the chelation agent, that is, 24.5% with deferasirox, 16.8% with deferoxamine, 6.5% with deferiprone, and 52.5% in those receiving combination regimens [325].

The timing of ICT initiation is a major determinant of gonadal outcomes in patients with transfusion‐dependent thalassemia. In an early cohort of 40 female patients with β‐thalassemia major, initiation of deferoxamine before the age of 10 years was associated with normal pubertal development and preserved gonadotropin responses, whereas delayed initiation was linked to primary amenorrhea and hypogonadotropic hypogonadism [221]. Combined intensive ICT regimens may provide additional benefits in reversing or mitigating gonadal dysfunction. In a cohort of 26 hypogonadal women treated with a combination of deferoxamine and deferiprone for 5–7 years, significant hormonal improvements were observed, including increases in basal and GnRH‐stimulated gonadotropins and estradiol levels; remarkably, six women achieved pregnancy [213, 326]. Despite these benefits, evidence for reversing established reproductive dysfunction remains limited, underscoring the need for early optimization of Fe control to preserve reproductive health.

#### 6.1.2 Hormone Replacement Therapy

For male hypogonadism, testosterone replacement therapy represents the cornerstone of treatment for patients not seeking paternity. Transdermal testosterone should be the preferred route of administration, as it allows for more precise dose titration [215] and avoids hepatic first‐pass metabolism, thereby reducing the risk of liver toxicity [327].

For women, estrogen replacement therapy is essential to alleviate symptoms related to estrogen deficiency, prevent long‐term complications, and improve the quality of life [328]. Transdermal estrogen administration should be strongly preferred, as it bypasses first‐pass hepatic metabolism, thereby reducing the impact on liver function [329]. In women with an intact uterus, estrogen has been administered in combination with a progestin to protect the endometrium [59].

#### 6.1.3 Supportive Therapies

Beyond chelation and hormone replacement, emerging therapeutic approaches are being explored. Direct inhibition of lipid peroxidation using small‐molecule ferroptosis inhibitors, such as Ferrostatin‐1 and Liproxstatin‐1, attenuated ferroptotic cell death and preserved cellular viability in Fe‐ and oxidative stress‐exposed tissues [27, 330, 331]. In germ cell systems, suppression of lipid peroxidation and modulation of key regulators of membrane polyunsaturated fatty acid composition, including *ACSL4*, reduced susceptibility to ferroptotic injury [25, 332]. Reinforcement of endogenous antioxidant defenses along the System Xc‐/glutathione/GPX4 axis similarly limited lipid peroxide accumulation and mitigated ferroptosis‐mediated cellular damage [29].

Experimental strategies aimed at enhancing tissue‐specific delivery have also been explored. In preclinical models, nanoparticle‐based formulations combined with Fe chelators have been shown to improve tissue penetration and reduce oxidative stress and DNA damage more effectively than chelators alone [333, 334, 335]. Although these approaches remain experimental, they offer proof‐of‐concept that overcoming pharmacokinetic and tissue barriers may enhance the therapeutic modulation of ferroptosis.

Future therapeutic development should prioritize the validation of ferroptosis biomarkers in well‐characterized Fe‐overload cohorts, the integration of tissue‐specific assessments of Fe burden and lipid peroxidation, and the exploration of combination regimens in which conventional ICT is complemented by targeted ferroptotic pathway modulation.

#### 6.1.4 Preclinical Animal Assays Targeting Fe

In addition to the established chelation and ferroptosis‐modulating strategies, a growing number of preclinical animal studies have explored novel interventions for Fe‐related pathologies (Table 3). In lipopolysaccharide (LPS)‐induced cognitive impairment, intracerebroventricular deferoxamine prevented hippocampal Fe accumulation, alleviated ferroptosis, and rescued memory deficits [336]. Ferroptosis inhibitors have also shown the therapeutic efficacy. For instance, Liproxstatin‐1 reduced both ferroptosis and necroptosis in a mouse stroke model, thereby limiting brain injury [337]; oltipraz attenuated cerebral I/R injury via Nrf2‐mediated suppression of oxidative stress and ferroptosis [338]; and the volatile inhibitor (2,2,6,6‐tetramethylpiperidin‐1‐yl)oxyl reduced pressure ulcer formation in cutaneous ischemia–reperfusion [339]. In hepatic ischemia–reperfusion, prophylactic ticlopidine reduced necrosis and fibrosis [340]. Furthermore, quercetin alleviated LPS/iE‐DAP‐induced liver injury by decreasing hepatic Fe accumulation through IL‐6/STAT3 signaling [341], and Ferrostatin‐1 alleviated experimental cerebral malaria by modulating immune cell functions and brain endothelial ferroptosis [342]. These preclinical in vivo findings have collectively supported the therapeutic potential of Fe chelation and ferroptosis inhibition across a wide range of inflammatory and ischemic disorders.

**TABLE 3:** Summary of preclinical animal assays targeting iron and other exemplified metal ions.

Metal ion	Disease model/context	Animal model	Intervention	Key findings	References
Fe	LPS‐induced cognitive impairment	Mouse model (C57BL/6)	Deferoxamine (DFO, 0.5 µg, i.c.v. pretreatment)	DFO prevented LPS‐induced hippocampal iron accumulation, modulated Fe‐related protein imbalance, alleviated ferroptosis, and significantly improved memory deficits in Morris water maze tests.	[336]
Fe	LPS‐induced acute lung injury	Mouse model	DFO or Fer‐1	Both DFO and Fer‐1 reversed lipid peroxidation and significantly attenuated lung injury; ferroptosis depends on iron overload and mitochondrial dysfunction.	[343]
Fe	Hepatic ferroptosis and insulin resistance	Mouse model + HepG2 cells	Deferasirox (iron chelator)	Fe overload induced hepatic ferroptosis and insulin resistance via JAK2/STAT3/SLC7A11 pathway inhibition; deferasirox improved hepatic insulin resistance.	[344]
Fe	Cerebral ischemia–reperfusion	Mouse model (MCAO)	Liproxstatin‐1 (ferroptosis inhibitor)	Fe promotes both ferroptosis and necroptosis in early reperfusion; Liproxstatin‐1 inhibited both pathways and reduced brain injury.	[337]
Fe	Cerebral ischemia–reperfusion injury	Mouse model	Oltipraz	Oltipraz attenuated cerebral ischemia–reperfusion injury through inhibiting oxidative stress and ferroptosis via *NRF2* pathway activation.	[338]
Fe	Cutaneous ischemia–reperfusion injury	Mouse model	TEMPO (volatile ferroptosis inhibitor)	TEMPO significantly reduced ulcer formation after cutaneous ischemia–reperfusion injury; reversed *GPX4, ACSL4*, and *4‐HNE* expression changes.	[339]
Fe	Hepatic ischemia–reperfusion injury	Mouse model (C57BL/6)	Ticlopidine	Prophylactic ticlopidine significantly reduced necrotic and fibrotic areas in liver, and serum ALT/AST levels.	[340]
Fe	Neonatal hypoxic–ischemic encephalopathy	Mouse model	*NUPR1* overexpression/*FTH1* modulation	*NUPR1* suppresses neuronal ferroptosis by increasing *FTH1* expression and improving Fe storage.	[345]
Fe	Hepatic ferroptosis gene signature	Mouse model	Transcriptome‐wide analysis	Established an Fe overload‐induced ferroptosis model and identified commonly regulated genes in hepatic ferroptosis response.	[346]
Fe	Type 1 diabetes‐related ferroptosis	Mouse model (NOD)	Spontaneous diabetes model	Fe accumulation and ferroptosis involved in early pathological changes in liver and kidney of NOD mice.	[347]
Fe	LPS/iE‐DAP‐induced liver injury	Mouse model	Quercetin	Quercetin alleviated ferroptosis and liver injury by decreasing hepatic Fe accumulation via IL‐6/STAT3 signaling.	[341]
Fe	Experimental cerebral malaria	Mouse model	Fer‐1	Fer‐1 alleviated experimental cerebral malaria by regulating immune cell functions and brain endothelial ferroptosis.	[342]
Cu	Periodontitis inflammation	Mouse model	TTM	TTM reduced cuproptosis, enhanced autophagy flux, decreased Ctsb levels, and alleviated macrophage dysfunction.	[348]
Cu	Acetaminophen‐induced liver injury	Mouse model	ATTM	ATTM modulated cuproptosis‐related gene expression; identified *SDHB, PDHA1, NDUFB2, NDUFB6* as hub genes in acetaminophen‐induced liver injury.	[349]
Cu	Sleep fragmentation‐induced myocardial ischemia–reperfusion injury	Male mouse	Cu overload model	16 weeks sleep fragmentation elevated cardiac Cu levels and exacerbated myocardial ischemia–reperfusion injury with increased cuproptosis and apoptosis; effects rescued by Cu modulation.	[350]
Cu	Polystyrene nanoplastics‐induced cognitive impairment	Mouse model (C57BL/6)	NAC, TTM, PD98059 (MAPK inhibitor)	Polystyrene nanoplastics increased cerebral Cu accumulation and induced cuproptosis (FDX1, LIAS, HSP70 dysregulation, DLAT oligomerization) via ERK‐MAPK pathway; NAC, TTM, and PD98059 reversed these effects.	[351]
Cu	Sepsis‐induced acute kidney injury	Mouse model	GSH	GSH significantly improved survival, reduced bacterial loads, attenuated inflammation and oxidative stress, restored kidney function, and reduced copper levels and *SLC31A1* expression.	[352]
Cu	Diabetic critical limb ischemia	Mouse model	Cu chelator tetrathiomolybdate	Tetrathiomolybdate inhibited cuproptosis in hindlimb muscles of diabetic critical limb ischemia mice; alleviated high glucose‐induced cuproptosis in HMEC‐1 cells.	[353]
Cu	Diabetic kidney disease	Mouse model	Qidantang granule	Qidantang granule ameliorated diabetic kidney disease by diminishing oxidative stress, modulating energy metabolism, and inhibiting cuproptosis.	[354]
Cu	Hyperoxia‐induced lung injury	Mouse model (C57BL/6)	Cuproptosis pathway analysis	Hyperoxia caused time‐dependent increase in *FDX1* expression, activated *SLC31A1*, and elevated copper levels in serum and lung tissue.	[355]
Cu	Gouty arthritis	Mouse models	SSG	MSU induced cuproptosis in chondrocytes; SSG suppressed chondrocyte cuproptosis and alleviated gouty arthritis.	[356]
Cu	Wilson's disease	Mouse model (CRISPR/Cas9‐generated R778L mutant)	Cu metabolism analysis	R778L mice exhibit aberrant Cu metabolism, pathological cellular injury, inflammation, severe hepatic fibrosis, and neurological manifestations resembling Wilson's disease.	[357]
Zn	Zn deficiency model establishment	Mouse model (C57BL/6J, male)	Dietary Zn deficiency	Validated dietary strategies to alter whole‐body Zn in mice; growth rates, plasma and urine Zn levels confirmed zinc status.	[358]
Zn	Lung inflammation and fibrosis in obese mice	Mouse model (obese)	Zn deficiency	Zn deficiency increased lung inflammation and fibrosis in obese mice by promoting oxidative stress.	[359]
Zn	Short bowel syndrome	Mouse model	Zn supplementation (dietary)	Zn‐supplemented mice achieved normal plasma Zn levels (12–25 µmol/L) and showed significant weight gain and improved survival; increased *KI67* and *PCNA* expression in intestinal tissue.	[360]
Zn	Autism spectrum disorder (*Shank2* knockout)	Mouse model (*Shank2* ^−/−^)	Dietary Zn supplementation	Zn supplementation reversed hyperactivity and social preference behavior in *Shank2* ^−/−^ mice, but did not alter working memory deficits.	[361]
Zn	Cognitive impairment (*ZnT3* deficiency)	Mouse model (*ZnT3*‐deficient)	Genetic deletion	*ZnT3* knockout decreased Zn levels in hippocampus and cortex, associated with progressive cognitive impairments via impaired dendritic spine plasticity and glucose metabolism.	[362]
Zn	Alzheimer's disease	Mouse (*NLRP3*‐deficient, *APP/PS1* double transgenic model)	Zn deficiency/supplementation	Zn deficiency accelerated cognitive decline via *NLRP3*‐dependent inflammation; zinc supplementation associated with reduced risk and slower cognitive decline.	[363]
Zn	DSS‐induced ulcerative colitis	Mouse model (DSS‐induced)	Natural Zn‐rich oyster peptides	Oyster peptides ameliorated weight loss, colonic damage, mucosal edema, goblet cell exhaustion, and crypt disappearance via antioxidation, anti‐inflammation, and intestinal barrier repair.	[364]
Ca	Medulloblastoma	Mouse model	Mibefradil (T‐type Ca channel inhibitor)	Mibefradil inhibited cancer cell xenograft growth and prolonged survival; T‐type calcium channels regulate medulloblastoma and represent a therapeutic target.	[365]
Ca	Lung cancer	Mouse model (BALB/c)	Amlodipine (L‐type Ca channel blocker)	Amlodipine exerted anticancer effects via modulating GSK‐3β, NF‐κB, PPARγ, and apoptosis pathways.	[366]
Ca	Bladder cancer/melanoma	Mouse model	Amlodipine + anti‐PD1	Amlodipine suppressed tumour growth by regulating *NFAT2* expression and enhanced anti‐PD1 immune checkpoint inhibitor therapy.	[367]
Ca	Multidrug resistance reversal	Mouse model	Manidipine (calcium channel blocker)	Manidipine reversed P‐gp‐mediated multidrug resistance through modulation of Ca signaling via *NFAT2*.	[368]
Ca	Cerebral ischemia–reperfusion brain injury	Mouse model	ML218 (T‐type calcium channel inhibitor)	ML218 modulated calcium binding protein, oxidative stress, and inflammation during cerebral ischemia–reperfusion injury.	[369]
Ca	Esophageal carcinoma	Mouse model	Amlodipine	Amlodipine inhibited proliferation and migration of esophageal carcinoma cells through induction of ER stress.	[370]

Abbreviations: 4‐HNE, 4‐hydroxynonenal; ACSL4, acyl‐CoA synthetase long‐chain family member 4; ALT, alanine aminotransferase; APAP, acetaminophen; APP, amyloid precursor protein APP; AST, aspartate aminotransferase; ATTM, ammonium tetrathiomolybdate; Ca, calcium; Cu, copper; DFO, deferoxamine; DLAT, dihydrolipoamide S‐acetyltransferase; DSS, dextran sulfate sodium; ER, endoplasmic reticulum; ERK, extracellular signal‐regulated kinase; Fe, iron; Fer‐1, Ferrostatin‐1; FTH1, ferritin heavy chain 1; GPX4, glutathione peroxidase 4; GSH, glutathione (reduced); HSP70, heat shock protein 70; i.c.v., intracerebroventricular; i.p., intraperitoneal; iE‐DAP, γ‐d‐glutamyl‐meso‐diaminopimelic acid; IL‐6, interleukin‐6; JAK2, Janus kinase 2; LIAS, lipoic acid synthetase; LPS, lipopolysaccharide; MAPK, mitogen‐activated protein kinase; MCAO, middle cerebral artery occlusion; MSU, monosodium urate; NAC, N‐acetylcysteine; NOD, nonobese diabetic; Nrf2, nuclear factor erythroid 2‐related factor 2; NUPR1, nuclear protein 1; PD‐1, programmed cell death protein 1; PS1, presenilin 1; SCFA, short‐chain fatty acids; SLC31A1, solute carrier family 31 member 1; SLC7A11, solute carrier family 7 member 11; SSG, syringaresinol‐4‐O‐β‐d‐glucoside; STAT3, signal transducer and activator of transcription 3; STZ, streptozotocin; TEMPO, (2,2,6,6‐tetramethylpiperidin‐1‐yl)oxyl; TTM, tetrathiomolybdate; Zn, zinc.

#### 6.1.5 Clinical Trial Evidence

Recent clinical trials have focused on optimizing chelation regimens for transfusion‐dependent Fe overload (Table 4). Real‐world efficacy data on dual oral Fe chelation (deferiprone + deferasirox) in 45 children with transfusion‐dependent thalassemia demonstrated a mean ferritin reduction from 2929 to 1962 ng/dL at 30 months, with 82.2% achieving ferritin <2000 ng/dL and no severe adverse effects [371]. A 2024 randomized controlled trial from Sri Lanka evaluated triple combination therapy (deferoxamine + deferasirox + deferiprone) in patients with β‐thalassemia and very high Fe overload (ferritin >3500 ng/mL). After 6 months, the triple therapy arm showed a mean ferritin reduction of 1094 ± 907 ng/mL compared with an increase of 82 ± 1588 ng/mL in the dual therapy control arm (*p* = 0.042). Cardiac T_2_* improved in 67% of triple therapy patients versus 20% of controls. However, deferiprone‐related arthralgia led to discontinuation in five patients [372]. A 2025 systematic review confirmed deferiprone's noninferiority to deferasirox with a favorable safety profile in pediatric patients, though long‐term data remained limited [373]. Two additional Phase 2 trials have been registered (NCT00901199: deferasirox plus deferoxamine; NCT00600938: deferasirox alone for myocardial Fe deposition), but their full publications are pending. Meanwhile, an ongoing Phase 3 study (NCT03037931) is examining ferric carboxymaltose for Fe deficiency anemia in heart failure and is currently enrolling patients.

**TABLE 4:** Summary of example clinical trials targeting iron and other exemplified metal ions.

Metal	Drug(s)/intervention	Mechanism	Indication	Phase/design	Key findings	Safety/adverse events	NCT ID	Status	Reference
Fe	Deferoxamine + deferasirox + deferiprone	Fe chelation	β‐Thalassemia	Phase 2/3 (*N* = 23)	Ferritin reduction: −1094 ± 907 ng/mL (triple) vs. +82 ± 1588 ng/mL (dual), *p* = 0.042; cardiac T_2_* improved in 67 vs. 20%.	Deferiprone‐related arthralgia led to discontinuation in five patients.	Not registered	Completed	[372]
Fe	Deferiprone + deferasirox	Fe chelation	Pediatric transfusion‐dependent thalassemia	Real‐world (*N* = 45)	Ferritin: 2929 → 1962 ng/dL at 30 months; 82.2% achieved ferritin <2000 ng/dL.	Not reported.	Not registered	Completed	[371]
Fe	Deferasirox + deferoxamine	Fe chelation	β‐Thalassemia	Phase 2 (*N* = 22)	Median ferritin decreased by 24% from 2465 to 1875 ng/mL (*p* = 0.002). All six subjects with elevated myocardial Fe showed improvement in MRI T_2_* (*p* = 0.031). The mean ± S.E. plasma NTBI declined from 3.10 ± 0.25 to 2.15 ± 0.29 µM (*p* = 0.028). The administration of DFX during infusion of DFO further lowered NTBI (−0.28 ± 0.08 µM, *p* = 0.004) and labile plasma Fe (LPI, −0.03 ± 0.01 µM, *p* = 0.006). The simultaneous administration of DFO and DFX rapidly reduced systemic and myocardial Fe, and provided an excellent control of the toxic labile plasma Fe species without an increase in toxicity.	Not reported.	NCT00901199	Completed	[374]
Fe	Deferiprone + deferoxamine	Fe chelation	β‐Thalassemia	Phase 4 (*N* = 213)	Sequential deferiprone‐deferoxamine significantly decreased serum ferritin vs. deferiprone alone (*p* = 0.005); no survival difference	Adverse events comparable between groups.	NCT00733811	Completed	[375]
Fe	BBI‐001	Nonabsorbed oral polymer binds dietary Fe	Hereditary hemochromatosis	Phase 1b (*N* = 24)	No results posted.	Not reported.	NCT05238207	Terminated	ClinicalTrials.gov
Fe	Deferasirox	Fe chelation	β‐Thalassemia	Phase 2 (*N* = 197)	Myocardial (m)T_2_* increased from baseline 11.6 to 15.9 ms in patients receiving deferasirox for 24 months (*n* = 74; geometric mean [*G*_mean_] ratio of month 24/baseline 1.38 [95% confidence interval 1.28, 1.49]) and from 10.8 to 14.2 ms in those receiving deferasirox (*n* = 29; *G*_mean_ ratio 1.33 [1.13, 1.55]; *p* = 0.93 between groups). Improved mT_2_* with deferasirox was evident across all subgroups evaluated irrespective of baseline myocardial (mT_2_* < 10 vs. ≥ 10 ms) or liver (LIC <15 vs. ≥15 mg Fe/g dw) Fe burden. Mean LVEF was stable and remained within normal limits with deferasirox or deferasirox. Liver Fe concentration decreased from high baseline values of 30.6 ± 18.0 to 14.4 ± 16.6 mg Fe/g dw at month 24 in deferasirox patients and from 36.8 ± 15.6 to 11.0 ± 12.1 mg Fe/g dw in deferasirox patients. Continued treatment of severely Fe‐overloaded beta thalassemia patients with deferasirox or deferasirox led to sustained improvements in myocardial Fe irrespective of high or low baseline myocardial or liver Fe burden, in parallel with substantial improvements in liver Fe.	Serious drug‐related adverse events reported in 6.8% of deferasirox and 6.9% of deferasirox patients.	NCT00600938	Completed	[376]
Fe	Ferric carboxymaltose	Intravenous Fe replacement	Fe deficiency anemia in heart failure	Phase 3 (*N* = 1132)	Ongoing, no results posted.	Not reported.	NCT03037931	Recruiting	ClinicalTrials.gov
Cu	Trientine tetrahydrochloride	Cu chelation	Wilson's disease	Phase 3 (*N* = 53)	Trientine tetrahydrochloride noninferior to penicillamine (mean difference −15.5 µg/L, 95% CI −34.5 to 3.6).	Penicillamine: 3 Severe adverse events (leukopenia, cholangiocarcinoma, HCC).	NCT03539952	Completed	[377]
Cu	Bis‐choline tetrathiomolybdate	Cu chelation	Wilson's disease	Phase 2 (*N* = 31)	Reduced exchangeable Cu and improved neurological symptoms.	Not reported.	NCT04422431	Completed	ClinicalTrials.gov
Cu	ALXN1840	Cu chelation	Wilson's disease	Phase 3 (*N* = 29)	16 (57%) treated with WTX101 either achieved or maintained normalized nonceruloplasmin‐bound Cu (NCC) corrected concentrations and 4 (14%) had at least a 25% reduction from baseline NCC corrected. Mean NCC corrected was reduced by 72% from baseline to week 24 (least squares mean difference −2.4 µmol/L [SE 0·4], 95% CI −3.2 to −1.6; *p* < 0·0001).	11 serious adverse events reported in seven (25%) patients and included psychiatric disorders, gait disturbance, elevated liver aminotransferases, and decline in neurological functioning.	NCT02273596	Completed	[378]
Cu	Trientine	Cu chelation	Wilson's disease	Phase 4 (*N* = 48)	No results posted.	Not reported.	NCT03299829	Completed	ClinicalTrials.gov
Cu	GC310 (AAV gene therapy)	Gene replacement (*ATP7B*)	Wilson's disease	Phase 1/2 (*N* = 15)	No results posted.	Not reported.	NCT07173933	Not recruiting	ClinicalTrials.gov
Cu	DMPS + DMSA	Cu chelation	Wilson's disease	RCT (*N* = 100 patients vs. 20 healthy)	DMPS/DMSA: faster neurological improvement (*p* = 0.023 at 1 year); urinary Cu: 300 vs. 200 µg/day.	Not reported.	Not registered	Completed	[379]
Cu	UX701 (AAV gene therapy)	Gene replacement (*ATP7B*)	Wilson's disease	Phase 1/2/3 (*N* = 82)	Ongoing, no results posted.	Not reported.	NCT04884815	Recruiting	ClinicalTrials.gov
Cu	Cu histidinate	Cu replacement	Menkes disease	Phase 2/3 (*N* = 60)	Ongoing, evaluating neurological outcomes and survival.	Not reported.	NCT00001262	Recruiting	ClinicalTrials.gov
Zn	Zn sulfate + diiodohydroxyquinolein	Zn supplementation	Uremic patients	RCT (*N* = 20)	Combined therapy: plasma Zn 9 → 14 µmol/L at 1 week.	Not reported.	Not registered	Completed	[380]
Zn	Zn sulfate + chelation	Zn supplementation	Lead poisoning	Preliminary (*N* = 20)	Reduced time to anemia correction vs. chelation alone.	Not reported.	Not registered	Completed	[381]
Zn	Zn citrate	Zn supplementation	Severe clinical infection in infants (0–59 days, Tanzania)	Phase 3 RCT (*N* = 3280)	Ongoing, evaluating 90‐day all‐cause mortality and treatment failure.	Not reported.	NCT06102044	Recruiting	ClinicalTrials.gov
Zn	Zn + nicotinamide riboside	Nutritional supplementation	Idiopathic pulmonary fibrosis	Phase 2 (*N* = 60)	Ongoing, evaluating feasibility, symptom improvement, and lung function.	Not reported.	NCT06567717	Recruiting	ClinicalTrials.gov
Ca	Ca channel blockers	VGCC inhibition	Pancreatic cancer	Retrospective cohort (*N* = 164)	CCB associated with improved OS: HR 0.496 (*p* = 0.007); median survival: 15.3 vs. 10.1 months.	Not reported.	Not registered	Completed	[382]
Ca	Denosumab	RANKL inhibitor	Pediatric osteogenesis imperfecta	Phase 2/3 (*N* = 153)	Terminated due to Ca‐related adverse events.	Hypercalcemia and hypocalcemia reported.	NCT02352753	Terminated	[383]
Ca	Amlodipine, nifedipine	Ca channel blockade	Hypertension	Phase 4 (*N* = 1215)	No results posted.	Not reported.	NCT05947448	Unknown	ClinicalTrials.gov
Ca	Ca gluconate	Ca supplementation	Traumatic hemorrhage hypocalcemia	Phase 1/2 (*N* = 81)	No results posted.	Not reported.	NCT06820645	Not recruiting	ClinicalTrials.gov
Ca	Mibefradil	T‐type Ca channel inhibition	High‐grade glioma	Phase 1 (*N* = 27)	Steady‐state peak plasma concentration at MTD was 1693 ± 287 ng/mL. ^18^F‐FLT PET imaging showed a significant SUV in two of 10 patients after 7 days of mibefradil, indicating biological activity.	The MTD of mibefradil was established at 87.5 mg orally four times daily when followed by TMZ in rHGG patients. Dose‐limiting toxicities included Grade 3 elevation of ALT/AST and sinus bradycardia. No other severe or unexpected toxicities were reported. The regimen was generally well tolerated in this patient population.	NCT01480050	Completed	[384]
Ca	Amlodipine + progestin	L‐type Ca channel blockade	Early‐stage endometrial cancer	Phase 2 (*N* = 140)	Ongoing, no results posted.	Not reported.	NCT06942377	Recruiting	ClinicalTrials.gov

Abbreviations: CI, confidence interval; deferasirox; DFO, deferoxamine; DFX, deferasirox; DMPS, dimercaptopropane sulfonate; DMSA, dimercaptosuccinic acid; HCC, hepatocellular carcinoma; LVEF, left ventricular ejection fraction; MTD, maximum tolerated dose; MRI, magnetic resonance imaging; NCC, nonceruloplasmin‐bound Cu; NTBI, nontransferrin‐bound Fe; OS, overall survival; RCT, randomized controlled trial; rHGG, recurrent high‐grade glioma; SAEs, serious adverse events; SUV, decline in proliferation signal; TEAEs, treatment‐emergent adverse events; TMZ, standard temozolomide; URTI, upper respiratory tract infection; VGCC, voltage‐gated Ca channel; WTX101, ALXN1840 (investigational Cu chelator).

### 6.2 Therapeutics Targeting Cu

The management of Cu‐associated disorders requires a stratified approach that reflects the nature and severity of Cu imbalance, ranging from Cu overload in Wilson's disease to Cu deficiency states and acute Cu poisoning. For Wilson's disease, the most well‐characterized Cu overload disorder, treatment strategies aim to reduce pathological Cu accumulation through chelation therapy or Zn‐mediated blockade of intestinal Cu absorption, with liver transplantation reserved for cases of pharmacological failure or severe hepatic decompensation. Beyond these established interventions, emerging therapeutic approaches, including modulation of the cuproptosis pathway, probiotics, gene therapy, and stem cell‐based techniques, are being actively explored to address the limitations of current treatments and to expand options for inherited Cu metabolism disorders such as Menkes disease. This section provides a comprehensive overview of current and emerging therapeutic strategies targeting Cu, from first‐line pharmacotherapy to novel experimental approaches.

#### 6.2.1 Cu Chelation Therapy

Chelation therapy is the cornerstone of treatment for Wilson's disease, with three main chelating agents being currently used [385, 386]. D‐penicillamine has traditionally served as the first‐line chelator; however, it is associated with significant adverse effects including neurological worsening, bone marrow suppression, and nephrotoxicity [387]. Trientine (triethylenetetramine) is an alternative chelator with a somewhat better safety profile than penicillamine, though its metal specificity remains suboptimal  [388, 389]. Tetrathiomolybdate (TTM), a third chelator that forms stable complexes with Cu, has demonstrated efficacy particularly in the initial treatment of neurological Wilson's disease, where it helps avoid the paradoxical neurological worsening occasionally observed with penicillamine [390].

Recent developments have focused on more specific Cu chelators. A novel drug candidate, TDMQ20, a specific Cu^2+^ chelator, has been evaluated in the TX mouse model of Wilson's disease [391]. Compared with D‐penicillamine and trientine, TDMQ20 exhibited superior efficacy at doses eight times lower, efficiently increasing ceruloplasmin ferroxidase activity and decreasing hepatic Cu load while showing no toxicity during long‐term administration in mice [391]. The enhanced efficacy of TDMQ20 is attributed to its design as a specific Cu^2+^ chelator that forms discrete, soluble complexes with Cu, whereas D‐penicillamine and trientine form oligomeric or cluster structures that may be retained in circulation or sequestered by proteins [391].

Monitoring of chelation therapy is based on clinical symptoms, liver function tests, and Cu metabolism parameters including 24‐h urinary Cu excretion and exchangeable Cu [261]. A study of children with Wilson's disease found that both 24‐h urinary Cu excretion and exchangeable Cu levels dropped significantly in the first year of chelation therapy and subsequently stabilized, demonstrating the utility of these parameters for monitoring treatment response  [392].

#### 6.2.2 Zn Therapy

Zn salts represent an alternative first‐line therapy for Wilson's disease, particularly for maintenance therapy in asymptomatic patients and for presymptomatic individuals. Zn acts by inducing intestinal metallothionein, which preferentially binds Cu and prevents its absorption, leading to a negative Cu balance. However, for patients with significant liver disease, chelators remain the preferred initial therapy [261]. Zn supplementation is a useful strategy because it competes with Cu for absorption in the intestines, thereby lowering total Cu levels in the body [386].

Nevertheless, prolonged Zn use may cause Cu deficiency, which requires careful monitoring [393]. The clinical manifestations of Cu deficiency include anemia, decreased immune function, impaired growth and development (especially in children), osteoporosis, abnormal cholesterol metabolism, and neurological disturbances [250]. For acquired Cu deficiency, treatment is directed at addressing the underlying cause, and oral Cu, usually administered as Cu sulfate, is provided [394]. However, acute Cu sulfate poisoning presents a particular clinical challenge, manifesting with multiorgan failure, hemolysis, methemoglobinemia, and acute kidney injury [395]. In such cases, chelation therapy with D‐penicillamine may not be effective, and Cu is not dialyzable [395]. Plasmapheresis (therapeutic plasma exchange) has been shown to remove protein‐bound Cu, thereby reducing plasma and intracellular concentrations, and has been successfully used to rescue patients with severe Cu sulfate poisoning who did not improve with chelation therapy  [396].

#### 6.2.3 Liver Transplantation

Liver transplantation has a well‐defined role in the management of Wilson's disease, particularly in patients presenting with acute liver failure when pharmacological therapy is insufficient to save life. It may also be considered in selected patients with advanced neurological disease that is unresponsive to medical therapy [261]. More broadly, individuals with severe liver damage caused by Cu excess may require liver transplantation, which may be necessary in extreme circumstances or if drug intolerance develops [397, 398].

#### 6.2.4 Supportive Therapies

Emerging therapeutic strategies targeting the cuproptosis pathway are being actively explored. Modulation of FDX1 activity and lipoylation pathways represents a potential approach for conditions beyond Wilson's disease. In addition, probiotics such as Heyndrickxia coagulans have shown protective effects against Cu‐induced toxicity in animal models, improving renal function, lipid profiles, and antioxidant enzyme activity, suggesting a potential supportive role in mitigating Cu intoxication [399]. Beyond these novel agents, drugs such as TTM offer an additional mechanism by binding Cu and preventing its intestinal absorption [400]. Given that combination regimens may enable more comprehensive management of Cu metabolism disorders, further exploration of multimodal therapeutic strategies is warranted to optimize Cu control and prevent toxicity.

For inherited Cu disorders such as Menkes disease, ongoing research is exploring potential therapies, including gene therapy [401, 402] and stem cell‐based techniques [403]. These strategies may ultimately enable the correction of underlying genetic defects and restoration of normal Cu metabolism.

#### 6.2.5 Preclinical Animal Assays Targeting Cu

Preclinical studies targeting Cu homeostasis have expanded beyond Wilson's disease to include inflammatory, ischemic, and metabolic conditions, often through modulation of cuproptosis (Table 3). In a murine periodontitis model, the Cu chelator TTM attenuated inflammation by reducing cuproptosis and enhancing autophagy flux [348]. In acetaminophen‐induced liver injury, ammonium TTM modulated cuproptosis‐related hub genes (*SDHB, PDHA1, NDUFB2, NDUFB6*) and reduced liver damage [349]. Sleep fragmentation exacerbated myocardial I/R injury in mice through cardiac Cu overload and cuproptosis, effects rescued by Cu modulation [350]. Polystyrene nanoplastics‐induced cognitive impairment was linked to cerebral Cu accumulation and ERK/MAPK‐mediated cuproptosis, which were reversed by NAC, TTM, or the MAPK inhibitor PD98059 [351]. In sepsis‐associated acute kidney injury, reduced glutathione improved survival and renal function by lowering Cu levels and *SLC31A1* expression [352]. Cu chelation also proved beneficial in diabetic critical limb ischemia, inhibiting cuproptosis in hindlimb muscles [353]. In gouty arthritis, syringaresinol‐4‐O‐β‐d‐glucoside suppressed chondrocyte cuproptosis and alleviated arthritis [356]. Moreover, the R778L mutant mouse model of Wilson's disease (CRISPR/Cas9‐generated) recapitulated aberrant Cu metabolism, hepatic inflammation, and neurological manifestations, providing a valuable platform for testing novel Cu‐targeted therapies [357]. These diverse models underscore the broad pathological relevance of cuproptosis and the potential of Cu chelators and cuproptosis modulators beyond inherited Cu overload syndromes.

#### 6.2.6 Clinical Trial Evidence

Several randomized controlled trials have evaluated Cu chelation strategies for Wilson's disease, ranging from head‐to‐head comparisons of existing agents to novel combination regimens and emerging gene‐based therapies (Table 4). The CHELATE trial (NCT03539952), a Phase 3 randomized, open‐label, noninferiority study, compared trientine tetrahydrochloride (TETA4) versus penicillamine for maintenance therapy in 53 adults with stable Wilson's disease. After 48 weeks, TETA4 was noninferior to penicillamine in serum nonceruloplasmin‐bound Cu (mean difference: −15.5 µg/L, 95% CI: −34.5 to 3.6). Notably, penicillamine was associated with three serious adverse events (leukopenia, cholangiocarcinoma, hepatocellular cancer), whereas none were reported for TETA4 [377]. Besides, NCT04422431 (Phase 2) evaluated bis‐choline TTM in Wilson's disease patients, demonstrating reduced exchangeable Cu levels and improvement in neurological symptoms, with no drug‐related serious adverse events reported [378]. NCT02273596 (Phase 3) assessed ALXN1840, a once‐daily Cu chelator, showing efficacy in reducing nonceruloplasmin‐bound Cu; headache and gastrointestinal events were the most common adverse effects. NCT03299829 (Phase 4) confirmed the long‐term effectiveness of trientine for maintenance therapy in Wilson's disease, demonstrating stable Cu control and a well‐tolerated safety profile. Beyond single‐agent comparisons, alternative treatment regimens have been explored in untreated patients. A randomized controlled trial in 100 patients with untreated Wilson's disease compared D‐penicillamine versus a combination of dimercaptopropane sulfonate (DMPS) plus dimercaptosuccinic acid (DMSA). The DMPS/DMSA combination improved neurological symptoms more rapidly (*p* = 0.023 at 1 year) and achieved higher urinary Cu excretion (300 vs. 200 µg/day), with faster brain metal clearance on susceptibility‐weighted imaging. After 3 years of treatment, brain metal content normalized to control levels [379].

Emerging gene‐based therapies are under active investigation (Table 4). NCT07173933 (Phase 1/2) is evaluating GC310, an AAV5‐ATP7B gene replacement therapy for Wilson's disease, with the trial currently not yet recruiting. NCT04884815 (Phase 1/2/3) is assessing UX701, another AAV‐based ATP7B gene therapy, which is currently recruiting. For Menkes disease, NCT00001262 (Phase 2/3) is evaluating subcutaneous Cu histidinate replacement therapy, with neurological outcomes and survival as primary endpoints.

### 6.3 Therapeutics Targeting Zn

#### 6.3.1 Zn Chelation Therapy

Zn chelation therapy is indicated in cases of Zn toxicity, either acute or chronic, when excessive Zn accumulation poses health risks [404, 405]. Chronic Zn toxicity, defined as daily intake of 50–150 mg/day, may arise from multiple acquired causes: occupational exposure (e.g., welding or galvanizing fumes), overprescribing of Zn‐containing medications, the use of Zn‐containing dental fixtures, or chronic overconsumption of over‐the‐counter Zn supplements [123, 125]. Unlike Fe and Cu overload disorders, which have well‐characterized inherited forms (e.g., HH and Wilson's disease, respectively), no inherited conditions of Zn overload due to mutations in Zn transporters have been reported. Thus, Zn toxicity is always acquired as an indication for Zn chelation therapy [125].

For severe cases of Zn toxicity that require pharmacological intervention, diethylenetriamine pentaacetate (DTPA) is the chelating agent of choice [124]. DTPA belongs to the polyaminocarboxylic acid class of chelators. It functions by forming stable, water‐soluble complexes with divalent metal ions, including Zn, thereby binding excess Zn and facilitating its elimination via renal excretion [124]. The specific clinical indications for DTPA chelation include: (i) acute Zn toxicity presenting with intractable vomiting, metabolic acidosis, or evidence of multiorgan involvement; (ii) massive overdose, defined as ingestion well above 1–2 g of elemental Zn, where endogenous clearance mechanisms are insufficient to prevent systemic toxicity; and (iii) rare cases of severe chronic Zn toxicity with documented systemic Zn overload that proves refractory to source removal [124, 125].

It is important to emphasize that chelation therapy is rarely required in the majority of Zn toxicity cases. Zn is efficiently cleared through endogenous homeostatic mechanisms, and the risk of systemic toxicity remains low unless extremely high doses are ingested [124, 125]. Furthermore, DTPA is not indicated for the management of metal fume fever, a self‐limited condition resulting from occupational inhalation of Zn oxide fumes. In this context, Zn is not absorbed systemically in sufficient quantities via the inhalation route to cause systemic overload, rendering chelation both unnecessary and inappropriate [124, 276].

#### 6.3.2 Cu Therapy

Cu therapy is indicated for the management of ZICD [127, 406, 407]. From a pharmacological perspective, Cu repletion therapy employs oral Cu salts, with Cu sulphate and Cu gluconate representing the primary formulations. These agents function by providing exogenous Cu to restore systemic Cu balance, thereby counteracting the deficiency state induced by chronic Zn excess [125, 129]. The mechanistic rationale for this intervention is grounded in the competitive relationship between Cu and Zn for intestinal absorption: exogenous Cu administration reduces Zn absorption, helping to restore Cu balance while simultaneously limiting further Zn accumulation from dietary or supplemental sources [103, 104, 105].

In the clinical practice, the first‐line and most critical intervention in all cases of chronic Zn toxicity remains the identification and removal of the source of excess Zn. This may involve discontinuing unnecessary Zn supplements, replacing Zn‐containing denture adhesives, eliminating occupational exposure to Zn compounds, or addressing other iatrogenic causes of Zn overload [100, 105, 123, 124, 125]. Following source removal, oral Cu therapy has been initiated as an adjunctive measure. The combination of source removal and oral Cu gluconate treatment is often sufficient to reverse the hematological abnormalities like anemia, neutropenia, and leukopenia that are commonly observed in ZICD [129].

Dosing of Cu supplementation is typically individualized based on the severity of Cu deficiency and the patient's clinical response. Standard therapeutic doses range from 2 to 4 mg per day [125, 129]. In the specific context of Wilson's disease management, where high‐dose Zn therapy (up to 6 mg/kg/day) is employed to maintain Cu balance through metallothionein induction, periodic assessment of Cu status is essential [261]. Rare cases may require minimal Cu supplementation (≥0.9 mg/day) to maintain systemic Cu balance within the desired therapeutic range, although this must be carefully monitored to avoid interfering with the therapeutic effects of Zn [56, 57, 408].

Regarding prognosis, the hematological abnormalities associated with ZICD typically respond favorably to Cu repletion combined with source removal, with resolution of anemia, neutropenia, and leukopenia occurring over weeks to months [128]. However, the prognosis for neurological deficits is considerably less favorable [409]. While neurological manifestations may improve partially with treatment, many patients never achieve full recovery and are left with permanent neurological sequelae [409]. This observation underscores the critical importance of early recognition, prompt diagnosis, and timely intervention in patients at risk for chronic Zn toxicity and ZICD, as prevention of neurological injury is far more effective than attempting to reverse established deficits [129].

#### 6.3.3 Supportive Therapies

Supportive and symptomatic therapies play an essential role in the management of Zn toxicity, particularly for acute Zn ingestion and metal fume fever, where specific antidotal therapy (e.g., chelation) is rarely indicated. These interventions are directed at alleviating specific symptoms, preventing complications, and supporting physiological function while endogenous clearance mechanisms eliminate excess Zn [410].

Antiemetics are indicated for the management of persistent vomiting associated with acute Zn ingestion, which typically occurs following excessive supplementation at doses exceeding 200 mg/day [103, 104, 105, 124, 125]. The antiemetics most commonly employed in this context include ondansetron, a selective 5‐HT_3_ receptor antagonist, and metoclopramide, a dopamine D_2_ receptor antagonist [411]. Ondansetron is particularly effective for vomiting of gastrointestinal origin and has a favorable side effect profile, with the most common adverse effect being headache rather than the extrapyramidal symptoms associated with metoclopramide [124, 125]. Metoclopramide also enhances gastric emptying through prokinetic effects, which may theoretically facilitate the transit of ingested Zn through the gastrointestinal tract, although this benefit is primarily theoretical in the setting of acute metal ingestion [124, 125].

Proton pump inhibitors, including omeprazole and pantoprazole, may be used empirically in the setting of acute Zn ingestion. These agents irreversibly inhibit gastric H^+^/K^+^ ATPase (the proton pump), thereby substantially reducing gastric acid secretion [124, 125]. The rationale for proton pump inhibitor administration is double folded: first, reduced gastric acidity may decrease Zn solubility and absorption, although this effect is not well quantified; second, acid suppression may protect the gastric mucosa from direct irritation caused by high concentrations of Zn salts, potentially reducing epigastric discomfort and preventing mucosal injury [124, 125]. It should be noted that the evidence supporting the use of proton pump inhibitors specifically in Zn ingestion is limited to clinical experience and extrapolation from other caustic or irritant ingestions, rather than controlled trials [124, 125].

Fluid replacement therapy is a cornerstone of supportive management as fluid and electrolyte imbalance secondary to vomiting and diarrhea is a common complication of acute Zn ingestion. The route of administration depends on the severity of dehydration. For mild to moderate cases, oral rehydration solutions containing balanced electrolytes and glucose are sufficient to correct deficits and maintain hydration [124, 125, 276]. For more severe presentations, characterized by intractable vomiting, significant dehydration, or hemodynamic instability, intravenous crystalloids such as normal saline (0.9% sodium chloride) or Ringer's lactate are indicated [124, 125, 276]. These solutions restore intravascular volume, correct electrolyte abnormalities, and support renal perfusion, thereby facilitating the endogenous renal clearance of absorbed Zn [124, 125, 276].

Antipyretics are indicated for the management of fever and constitutional symptoms associated with metal fume fever, an acute influenza‐like syndrome resulting from occupational inhalation of Zn oxide fumes. Common occupational exposures occur during welding, galvanizing, and brass foundry work. Metal fume fever typically presents 4–12 h after exposure with fever, chills, myalgia, headache, and a metallic taste in the mouth [276]. Paracetamol (acetaminophen) acts centrally on COX‐2 in the hypothalamus to reduce fever by inhibiting prostaglandin synthesis in the thermoregulatory center [412]. It is generally well tolerated at therapeutic doses and is the preferred antipyretic in patients with contraindications to NSAIDs [276]. NSAIDs, such as ibuprofen, inhibit both peripheral COX‐1 and COX‐2, thereby reducing prostaglandin‐mediated inflammation and providing dual antipyretic and analgesic effects [413]. Ibuprofen is particularly effective for alleviating the myalgia and headache that frequently accompany metal fume fever [276].

Bronchodilators are indicated to relieve bronchospasm and improve airflow in patients suffering from respiratory symptoms including cough, wheezing, and dyspnea [276]. Salbutamol (also known as albuterol) is a short‐acting β_2_‐adrenergic receptor agonist that produces rapid bronchodilation by relaxing airway smooth muscle [414]. At the cellular level, binding of the agonist to β_2_ receptors on bronchial smooth muscle activates the Gs‐protein‐coupled enzyme adenylate cyclase, which converts adenosine triphosphate (ATP) to cAMP [414]. The subsequent rise in intracellular cAMP activates protein kinase A, which phosphorylates multiple target proteins, leading to decreased intracellular Ca concentrations and ultimately smooth muscle relaxation [414]. Salbutamol is administered via nebulizer or metered‐dose inhaler, with or without a spacer device. The onset of action occurs within 5–15 min, and the duration of effect is typically 4–6 h [276]. In the setting of metal fume fever, bronchodilator therapy is reserved for patients with demonstrable wheezing or objective evidence of airflow obstruction, as most individuals experience only mild, self‐limited respiratory symptoms that resolve with removal from exposure and supportive care alone [276].

Supplemental O_2_ is indicated for patients with metal fume fever who present with significant respiratory distress, hypoxemia (O_2_ saturation below 92% on room air), or pre‐existing cardiopulmonary disease that may be exacerbated by fume exposure [413]. Oxygen is typically delivered via nasal cannula at flow rates of 2–4 L per minute or via face mask at higher concentrations as needed to maintain adequate oxygenation [276]. Most patients with metal fume fever do not require supplemental O_2_, as respiratory symptoms are usually mild and self‐limited; however, its availability is important for those with more severe presentations or underlying pulmonary compromise [276].

#### 6.3.4 Preclinical Animal Assays Targeting Zn

Although Zn toxicity is acquired and rarely requires chelation, preclinical studies have investigated the consequences of Zn deficiency and the benefits of Zn supplementation in various disease models (Table 3). Dietary Zn deficiency in C57BL/6J mice was validated by reduced growth and lower plasma/urine Zn levels, providing a reliable model for studying Zn depletion [358]. In obese mice, Zn deficiency increased lung inflammation and fibrosis via oxidative stress [359]. In a murine short bowel syndrome model, dietary Zn supplementation normalized plasma Zn (12–25 µmol/L), promoted weight gain, improved survival, and increased intestinal proliferation markers Ki67 and PCNA [360]. In autism‐related *Shank2* knockout mice, Zn supplementation reversed hyperactivity and social preference deficits, though working memory remained unaffected [361]. *ZnT3* knockout mice, which exhibited reduced hippocampal and cortical Zn, developed progressive cognitive impairments associated with impaired dendritic spine plasticity and glucose metabolism [362]. In an Alzheimer's model, Zn deficiency accelerated cognitive decline via NLRP3‐dependent inflammation, while supplementation slowed progression [363]. Finally, natural Zn‐rich oyster peptides ameliorated DSS‐induced ulcerative colitis through antioxidant, anti‐inflammatory, and intestinal barrier‐protective effects [364]. These studies highlight the critical role of Zn status in neurological, metabolic, and inflammatory diseases, and support further exploration of Zn supplementation as an adjunctive therapy.

#### 6.3.5 Clinical Trial Evidence

No targeted chelation trials have been conducted specifically for Zn toxicity, reflecting the fact that all cases of Zn overload are acquired and management is primarily directed at identifying and removing the source of excess Zn [124]. However, two early clinical studies have explored pharmacological modulation of Zn status. A 1995 randomized trial in 20 uremic patients evaluated Zn sulfate (100 mg/day) with or without the chelator diiodohydroxyquinolein  [380]. Combined therapy increased plasma Zn from 9 ± 0.1 to 14 ± 1.6 µmol/L after 1 week, a greater effect than either agent alone, suggesting that diiodohydroxyquinolein may facilitate Zn absorption or redistribution (Table 4). In lead poisoning (saturnism), a preliminary study of 20 patients found that adding Zn sulfate (400 mg/day) to standard chelation therapy reduced the time required for anemia correction compared with chelation alone  [381] (Table 4). These represent the only available clinical data on Zn modulation; and no modern randomized controlled trials targeting primary Zn overload has been reported.

More recently, several ongoing trials are investigating Zn supplementation in infectious and inflammatory conditions. NCT06102044 is a randomized controlled trial evaluating Zn citrate (5 mg BID for 14 days) for severe clinical infection in infants aged 0–59 days in Tanzania, with 90‐day all‐cause mortality as the primary endpoint [415]. NCT06567717 is assessing Zn combined with nicotinamide riboside in patients with idiopathic pulmonary fibrosis, examining feasibility, symptom improvement, and lung function. Both trials are currently recruiting (Table 4).

### 6.4 Therapeutics Targeting Ca

The complexity of widespread Ca channels, transporters, and pumps, together with their diverse activation processes, offers an abundance of potential targets for pharmacological regulation and cancer chemotherapy. Progress in understanding the intracellular Ca signaling network, especially the structure of these channels, transporters, and pumps, has significantly advanced the field of drug design and development, with particular focus on selective inhibitors and regulators. A number of compounds or antibodies targeting cancer‐associated Ca signaling proteins have been studied in preclinical research or even in clinical trials, and several have demonstrated promising potential for use in cancer therapy [416, 417, 418, 419].

#### 6.4.1 Ca‐ATPase Inhibitors

The sustained elevation of cytoplasmic Ca is toxic to cells and can activate cell death signaling pathways. Ca‐ATPases can be targeted by inhibiting these pumps to generate toxic cytosolic Ca concentrations that induce either apoptosis or necrosis [420, 421, 422]. A selective inhibitor of the SERCA pump, thapsigargin, inhibits Ca uptake into the ER and depletes ER Ca stores. The application of thapsigargin as a chemotherapeutic agent has been extensively studied in cancers [423]. However, a barrier preventing the direct use of thapsigargin as a clinically effective drug is its nonselectivity, as thapsigargin disrupts intracellular Ca homeostasis not only in cancer cells but also in normal cells. Thus, research efforts have focused on developing tumour‐targeted thapsigargin derivatives. One successful example is G202 (also termed mipsagargin), in which an analogue of thapsigargin is conjugated to a prostate‐specific membrane antigen (PSMA)‐targeting peptide. PSMA is overexpressed in prostate cancer cells and most tumor endothelial cells but not in normal vasculature or normal tissue epithelium. As a prodrug, G202 itself is nontoxic because it cannot enter cells due to its size. Once it reaches the tumour, it binds to PSMA, which cleaves the peptide and releases the active cytotoxic thapsigargin analogue. G202 significantly inhibits tumour progression in prostate, breast, and bladder cancers while presenting minimal toxicity to host animals. G202 has shown promising results in several preclinical studies and is currently in Phase 2 clinical trials for breast cancer [424].

Cyclopiazonic acid is another SERCA inhibitor that has been studied in various cancer models, including hepatoma, colon, cervical, and breast cancers, as well as nasopharyngeal carcinoma [425, 426]. RL71, a small molecule targeting SERCA2 at a novel site, has been evaluated in the treatment of human colorectal cancer [427]. Additionally, a PMCA‐selective inhibitor, [Pt(O,O’‐acac)(γ‐acac)(DMS)], has been shown to rapidly induce apoptosis in MCF‐7 breast cancer cells, potentially through the generation of ROS in addition to cytosolic Ca elevation [428].

#### 6.4.2 Voltage‐Gated Ca Channel Inhibitors

The first well‐studied family of compounds targeting Ca signaling are the inhibitors of voltage‐gated Ca channels (VGCCs), which are widely used in cardiovascular and nervous system diseases. As accumulating evidence reveals the important roles of VGCCs in a number of cancers [429, 430, 431, 432], many investigators have launched studies to repurpose United States Food and Drug Administration (US FDA)‐approved drugs targeting VGCCs for cancer treatment [433]. As early as the 1990s, structurally unrelated L‐type VGCC antagonists were tested for their potent inhibitory effects on breast tumour progression [433, 434]. The dihydropyridine Ca channel blocker (CCB) amlodipine was found to inhibit the growth of human epidermoid carcinoma A431 cells both in vitro and in vivo by arresting the cell cycle at the G1 phase and reducing retinoblastoma protein phosphorylation, as well as the expression levels of Cyclin D1 and cyclin‐dependent kinase 4 [435].

Another interesting case is mibefradil, a T‐type and L‐type CCB originally used as an antihypertensive agent. Although it was voluntarily withdrawn from the market due to its side effect of inhibiting cytochrome P450 enzymes 2D6, 3A4, and P‐glycoprotein, mibefradil was shown to efficiently reduce tumour size and improve survival rates in glioma animal models as well as in patient‐derived pancreatic xenograft models [436]. Consequently, mibefradil was repurposed for pancreatic cancer and high‐grade glioma therapy. The US FDA swiftly approved mibefradil as an orphan drug for pancreatic cancer treatment in 2008 [437], and its use for glioma treatment has also moved into clinical trials [438]. Moreover, a mibefradil‐derived novel compound, NNC‐55‐0396, was developed to selectively target Ca channels and exhibits less inhibitory effect on cytochrome P450 3A4. This new derivative appears to be a promising chemotherapeutic agent, as it effectively inhibits angiogenesis in cancer cell lines with minimal off‐target effects [439]. KYS05047, another T‐type CCB, induces G1 phase cell cycle arrest by decreasing intracellular Ca levels in human lung adenocarcinoma A549 cells [440]. Ghrelin, which increases T‐type Ca channel protein expression, has been shown to inhibit proliferation in PC‐3 human prostate carcinoma cells [441].

#### 6.4.3 TRP Channel Regulators

The imidazole compound SKF‐96365 and related antimycotic compounds, including econazole, miconazole, and clotrimazole, can inhibit Ca release‐activated Ca channels (CRACs) and some TRP channels. While SKF‐96365 was first described as a blocker of receptor‐mediated Ca entry in human platelets, neutrophils, and endothelial cells [442], it was later used to inhibit ovarian cancer cell growth and tumourigenesis by reducing the activities of different subtypes of TRPC channels [443]. Treatment with SKF‐96365 was reported to enhance radiosensitization in glioblastoma cells that express high levels of TRPC6 channels [419]. 2‐APB (2‐aminoethoxydiphenyl borate) is another commonly used inhibitor that was initially identified as a noncompetitive antagonist of IP3 receptors at relatively high concentrations (≥100 µmol/L) [444, 445]. Several 2‐APB‐derived compounds, such as DPB‐162AE and DPB‐163AE, have been constructed as dimers of 2‐APB and are over 100‐fold more potent than 2‐APB at inhibiting store‐operated Ca entry (SOCE) without affecting IP_3_R function at such concentrations [446].

TH‐1177, a synthetic compound mimicking dihydropyridines developed as a TRPV channel blocker, inhibited prostate cancer cell proliferation in vitro and in vivo [447]. However, TH‐1177 preferentially inhibits TRPV5 but is less effective against TRPV6, which is the most abundant Ca entry channel in prostate cancer cells, with expression levels as high as 45‐fold more than TRPV5 [448]. TH‐1177 was subsequently modified to possess high selectivity for TRPV6, and this new agent has demonstrated significantly improved inhibitory effects on cell proliferation in prostate and breast cancers [448]. SOR‐C13 and SOR‐C27, derived from the C‐terminus of soricidin, bind TRPV6 with high affinity in ovarian cancer cells. SOR‐C13 is currently in Phase 1hematological clinical trials (NCT01578564, NCT03784677) for advanced cancers in which TRPV6 channels are normally overexpressed [449].

GSK1016790A is a selective TRPV4 channel agonist that is at least 300‐fold more potent than the commonly used TRPV4 activator 4α‐PDD. TRPV4 regulates tumour angiogenesis and vessel maturation, and GSK1016790A has been proposed for use together with other anticancer drugs, such as cisplatin, to improve tumour penetration for more effective cancer therapy [450, 451]. Capsaicin, a well‐known activator of TRPV1, was shown to induce apoptosis through its action on TRPV6 (independent of TRPV1), followed by activation of calpains, in human small cell lung cancer cells in vitro and in vivo [452, 453, 454]. The TRPV antagonist capsazepine has been demonstrated to effectively inhibit oral squamous cell carcinoma cell growth in vivo, with an anticancer mechanism that may also rely on ROS release independent of TRPV1 [455].

(−)‐Englerin A is a selective and potent activator of TRPC4 and TRPC5 channels, resulting in massive Ca influx followed by cell death and retarded tumour cell growth [456, 457]. However, its severe lethal side effects must be resolved before it can be considered a potential therapeutic agent. 20‐GPPD, a metabolite of ginseng saponin, induced TRPC‐mediated Ca influx and apoptosis in CT‐26 cells and reduced tumour mass by 75% in vivo, although the exact molecular target remains unknown [458]. Cannabidiol, a nonpsychotropic cannabinoid, stimulated TRPV1 and displays anti‐invasive action in human lung carcinoma A549 cells, dependent on the effect of cannabinoid receptors and TRPV1 [459, 460].

#### 6.4.4 Orai and STIM Inhibitors

Among the many known compounds targeting CRAC or SOCE, Orai channel blockers are particularly well studied. Because CRAC channels present highly selective Ca conductance, they are subject to blockage by trivalent ions such as La^3+^ and Gd^3+^, which directly block the CRAC pore formed by the I‐II loop region of Orai1. However, this effect is not specific to CRAC, as La^3+^ and Gd^3+^ also block other Ca channels, including Cav, TRP channels, and PMCA [461, 462, 463].

SKF‐96365 was the first Orai1 inhibitor to be investigated in cancer research. In breast cancer models, SKF‐96365 inhibited cell migration in vitro and reduced tumor growth and metastasis in vivo [464]. Similarly, in esophageal cancer cells, SKF‐96365 suppressed Orai1‐mediated SOCE and intracellular Ca oscillations, leading to significantly retarded tumor growth in nude mice [416]. BTP2 (also known as YM‐58483 or Pyr2) is a pyrazole derivative that acts as a potent inhibitor of both Ca release‐activated Ca (CRAC) channels and TRPC‐mediated Ca entry [465, 466]. However, an alternative mechanism has been proposed: BTP2 may inhibit Ca influx and cytokine release primarily by depolarizing the cell membrane via TRPM4 activation, thereby reducing the electrochemical driving force for Ca entry [467].

AnCoA4 is a novel STIM‐Orai inhibitor identified using high‐throughput screening of small‐molecule microarrays containing minimal functional domains of STIM1 and Orai1 [468]. AnCoA4 inhibited SOCE at submicromolar concentrations and attenuates T‐cell activation both in vitro and in vivo [468]. Mechanistically, AnCoA4 binds to the C‐terminus of Orai1, directly blocking Ca influx through the channel and reducing Orai1 recruitment into puncta; it also inhibits the activity of the constitutively active Orai1V102C channel independently of STIM1 [468]. ML‐9, an inhibitor of myosin light‐chain kinase, was found to disperse STIM1 puncta and thereby inhibit SOCE [469]. Although its precise molecular target and mechanism of action remain incompletely defined, ML‐9 is the only known inhibitor that suppresses SOCE through interference with STIM1 translocation [469]. As a monotherapy, ML‐9 effectively induces prostate cancer cell death associated with autophagy in a concentration‐ and Ca‐dependent manner. Furthermore, combining ML‐9 with existing anticancer drugs such as docetaxel significantly promotes cancer cell death, suggesting that ML‐9 may serve as a promising adjuvant to conventional chemotherapy [470].

RO2959 is a novel, potent, and selective inhibitor of SOCE, with an IC_50_ of approximately 40 nmol/L [471]. It suppresses a broad range of Ca‐dependent cellular functions, including gene expressions indirectly, rather than through direct channel blockade [471]. However, its potential anticancer activity and the precise molecular basis of its action, including whether it modulates STIM1 function or dynamics, remain to be elucidated.

Two pyrazole derivatives, GSK‐5503A and GSK‐7975A, have been identified as slow‐onset inhibitors of Orai1‐ and Orai3‐mediated SOCE currents, without affecting STIM1–Orai1 coupling. Their inhibitory effects require several minutes to reach full efficacy, suggesting a mechanism of action that is independent of the CRAC channel activation process itself. Notably, these compounds also inhibit TRPV6 channels, possibly reflecting structural similarities in the binding sites between CRAC and TRPV6 channels [472, 473]. CM2489 and CM3457 represent another class of promising SOCE inhibitors, with demonstrated activity in lymphocytes and T‐cell‐derived cytokine production. CM2489 is the first CRAC channel inhibitor to have been tested in humans and represents a promising lead for the development of novel therapeutics for human diseases, including cancers  [474].

Recombinant monoclonal antibodies targeting Orai1 exhibit strong and specific binding to amino acid residues 210–217 in the second extracellular loop of the channel [475]. Among these, clone 2C1.1 effectively inhibited CRAC currents in Orai1‐high‐expressing Jurkat T cells and HEK293 cells [476]. Functional assays further demonstrated that these antibodies potently suppressed NFAT‐dependent gene transcription in Jurkat cells and inhibit thapsigargin‐ or ionomycin‐induced cytokine secretion from human T cells [475]. Another specific anti‐Orai1 mAb, also directed against the second extracellular loop, has been shown to block T‐cell proliferation and cytokine production both in vitro and in vivo [477]. Mechanistic investigations suggested that antibody‐bound Orai1 underwent internalization, leading to a loss of functional SOCE activity [477]. Although current development of anti‐Orai1 mAb‐based therapies remains largely confined to immune‐related disorders, this strategy holds considerable promise for future application in cancer treatment.

#### 6.4.5 Other Agents Targeting Ca Signaling

Several regulators targeting other Ca channels, transporters, or pumps have been used in various areas, including immune suppression, antihypertensive therapy, and oncology. Xestospongin B, a specific IP_3_R inhibitor, has been demonstrated to reduce proliferation and colony formation while selectively inducing necrotic cell death in tumourigenic breast cells, with minimal effects on nontumourigenic lines [478, 479]. Similarly, caffeine, a RyR agonist, induced apoptosis in prostate cancer cells, while another agonist, 4‐chloro‐m‐cresol, inhibited breast cancer cell growth [480, 481].

Several compounds targeting the NCX have demonstrated proapoptotic activity through mitochondrial Ca overload. For example, OSW‐1 induced apoptosis in leukemia cells via this mechanism [482]. SKF‐96365 enhanced the reverse mode of NCX1 in glioblastoma cells, causing cell cycle arrest at the S and G2 phases independently of TRPCs [483]. Brilliant blue G, a P2×7 antagonist, exhibited an inhibitory effect on glioma growth [484]. A number of P2×7 receptor regulators, such as the antagonists A‐438079 and A‐740003, have been primarily investigated for pain relief [485, 486]. Given the established roles of P2 receptors in various cancers, these antagonists may hold potential as dual‐function therapeutics, combining anticancer activity with analgesic effects [487, 488, 489, 490, 491].

In summary, the therapeutic management of metal‐related disorders has evolved from symptomatic support to targeted correction of metal imbalance at its source. Fe overload is effectively managed with phlebotomy or chelation, and hormone replacement has substantially improved outcomes for hypogonadism; nevertheless, damage to organs such as the pituitary, heart, and pancreas remains largely irreversible. Emerging ferroptosis inhibitors and nanoparticle‐based chelators may offer tissue protection beyond simple metal removal, although clinical translation awaits confirmation. Cu chelation with D‐penicillamine or trientine, combined with Zn acetate for maintenance, can effectively control Wilson's disease; however, neurological deterioration may persist despite treatment, and novel agents such as TDMQ20 remain in early development. Zn toxicity, unique among these metals, has no inherited forms and is managed primarily by source removal, with Cu repletion for ZICD; chelation is rarely required. Ca channel modulators have demonstrated efficacy in cardiovascular disease and are being repurposed for oncology, though concerns regarding selectivity and toxicity remain. Looking forward, several priorities emerge for therapeutic development: validation of predictive biomarkers, design of tissue‐specific delivery systems, and integration of metal‐targeted therapies with conventional regimens. The ultimate goal is precision metallotherapy, that is, tailoring interventions to individual metal profiles, genetic susceptibilities, and disease stages, to not only manage but ultimately prevent the irreversible complications of metal imbalance.

#### 6.4.6 Preclinical Animal Assays Targeting Ca

Ca channel modulators have been extensively evaluated in preclinical cancer and ischemia models (Table 3). In an xenograft medulloblastoma model, the T‐type Ca channel inhibitor mibefradil suppressed tumour growth and prolonged survival [365]. The L‐type blocker amlodipine exhibited anticancer effects in a murine lung cancer model via modulation of GSK‐3β, NF‐κB, PPARγ, and apoptosis pathways [366]. In bladder cancer and melanoma syngeneic models, amlodipine enhanced anti‐PD‐1 immunotherapy by regulating *NFAT2* expression [367]. Manidipine reversed P‐glycoprotein‐mediated multidrug resistance in mouse xenografts through Ca/NFAT2 signaling [368]. In cerebral ischemia–reperfusion, the T‐type inhibitor ML218 modulated Ca‐binding proteins, oxidative stress, and inflammation [369]. Amlodipine also inhibited proliferation and migration of esophageal carcinoma cells in xenografts through ER stress induction [370]. These findings reinforce the repurposing potential of clinically approved CCBs for oncology and neuroprotection, and support ongoing clinical trials in these areas.

#### 6.4.7 Clinical Trial Evidence

CCBs have been investigated for repurposing in oncology. A retrospective cohort study of 164 patients with unresectable pancreatic ductal adenocarcinoma found that CCB prescription was associated with significantly improved overall survival on adjusted Cox regression (HR 0.496, 95% CI 0.297–0.827, *p* = 0.007). Median survival was 15.3 months for CCB users versus 10.1 months for nonusers [382] (Table 4). Mechanistically, manidipine, a clinically approved CCB, was shown to reverse P‐glycoprotein‐mediated multidrug resistance in colorectal and lung cancer cells by reducing intracellular Ca (52% reduction, *p* < 0.001) and downregulating NFAT2. In vivo, manidipine (3.5 mg/kg) synergized with paclitaxel to inhibit tumour growth by 68% in xenograft models [368]. These findings support ongoing investigation of CCBs as adjuvant cancer therapies.

A Phase 1 trial (NCT01480050) evaluated mibefradil, a selective T‐type Ca channel inhibitor, in recurrent high‐grade gliomas. Twenty‐seven patients were enrolled (20 Grade IV, 7 Grade III; median age 50 years; median Karnofsky performance status 90). The maximum tolerated dose (MTD) of mibefradil was established at 87.5 mg orally four times daily, followed by standard temozolomide (150–200 mg/m^2^ for 5 days per 28‐day cycle). Dose‐limiting toxicities were Grade 3 transaminase elevation and sinus bradycardia. Steady‐state peak plasma concentration at MTD was 1693 ± 287 ng/mL. ^18^F‐FLT PET imaging showed a significant decline in proliferation signal in two of 10 patients after 7 days of mibefradil. The regimen was well tolerated and warrants further investigation. Additionally, NCT06942377 is currently recruiting to evaluate amlodipine combined with progestin for conservative therapy in early‐stage endometrial cancer (Table 4).

Beyond oncology, several Ca‐targeting trials have been registered for other indications, though some have been terminated or lack published results. NCT02352753 (Phase 2/3) evaluated denosumab for pediatric osteogenesis imperfecta; the trial was terminated early due to Ca‐related adverse events, including hypercalcemia and hypocalcemia. NCT05947448 (Phase 4 observational) is assessing CCBs (amlodipine, nifedipine) for essential hypertension; as of 2026, the trial is ongoing with no results posted. NCT06820645 (Phase 1/2 feasibility) is evaluating Ca gluconate for hypocalcemia correction in traumatic hemorrhage; this trial was registered in 2025 and is currently ongoing without published results (Table 4).

## 7 Conclusions

This comprehensive review has traversed the landscape of metal ions in human health and disease, with Fe as the central anchor and Cu, Zn, Ca as equally essential but chemically distinct counterparts. Several key conclusions emerge from this synthesis, each underscoring Fe's preeminent role while acknowledging the unique contributions of the other metals.

First, the chemical properties of each metal dictate its biological functions. Transition metals, Fe and Cu, exploit redox versatility to catalyze electron transfer reactions, yet Fe, as the most abundant transition metal in the body, is uniquely positioned at the crossroads of O_2_ transport, energy metabolism, and oxidative stress. Nontransition metals (Zn and Ca) provide stable structural and signaling functions without redox cycling. This classification, while useful, is not absolute: Zn participates in redox regulation through antioxidant mechanisms, and Ca influences mitochondrial redox balance via metabolic control. Among these, Fe's Fenton chemistry is both indispensable and dangerously potent, making its homeostasis the most tightly regulated of all essential metals.

Second, metal homeostasis is maintained by an intricate network of transporters, chaperones, storage proteins, and regulatory molecules. For Fe, the hepcidin–ferroportin axis serves as the master systemic regulator, while Cu utilizes ATP7A/B chaperones, Zn is buffered by metallothioneins, and Ca is handled by the MCU complex and calbindins. Genetic or acquired disruptions in these systems lead to characteristic deficiency or overload syndromes, with Fe disorders (anemia and hemochromatosis) being the most prevalent and best characterized, followed by Cu (Wilson's disease, Menkes disease), Zn (acrodermatitis enteropathica), and Ca (hypo‐/hyperparathyroidism).

Third, the discoveries of ferroptosis and cuproptosis have revolutionized our understanding of metal‐induced cell death. Fe drives lipid peroxidation through Fenton chemistry, generating phospholipid hydroperoxides that perpetuate membrane damage, while Cu triggers proteotoxic stress by binding lipoylated TCA cycle enzymes. Ferroptosis is now recognized as a central driver of tissue injury in hemochromatosis, neurodegeneration, cardiac ischemia–reperfusion, and kidney injury, whereas cuproptosis is particularly implicated in Wilson's disease and Cu‐related malignancies. These metal‐dependent death pathways offer distinct therapeutic windows, with ferroptosis inhibitors already showing broad preclinical promise.

A critical insight that warrants further emphasis is the intimate connection between metal imbalance and inflammation. Mitochondrial dysfunction caused by Fe and Cu excess releases damage‐associated molecular patterns (mtDNA, mtROS, cardiolipin) that activate innate immune receptors and inflammasomes, perpetuating a vicious cycle of oxidative stress and inflammation. This “mito‐inflammation” is a common feature of aging, metabolic syndrome, and chronic inflammatory diseases. Fe overload, in particular, is a potent driver of this cascade due to its abundance and catalytic efficiency, suggesting that restoring Fe homeostasis may yield broad anti‐inflammatory benefits beyond correcting anemia or Fe toxicity.

Diagnosis of metal‐related disorders requires an integrated approach combining clinical assessment, biochemical testing, imaging, and genetic analysis. Early diagnosis is critical, as neurological and endocrine damage may become irreversible. Of note, T_2_* MRI has revolutionized noninvasive Fe quantification in liver, heart, and pituitary, serving as a model for precision diagnostics; analogous tools for Cu, Zn, and Ca are urgently needed. Clinicians must maintain a high index of suspicion for metal imbalance, particularly Fe overload, in patients with unexplained multisystem symptoms involving the liver, heart, brain, and endocrine glands.

Therapeutic strategies have advanced significantly over recent decades. Fe chelation with deferoxamine, deferasirox, and deferiprone, combined with hormone replacement, has transformed outcomes in transfusion‐dependent anemias. Cu chelation with D‐penicillamine or trientine, along with Zn acetate for maintenance, effectively manages Wilson's disease. For Ca disorders, calcilytics, calcimimetics, and channel modulators are in various stages of development. Looking forward, emerging approaches, mitochondrial transplantation, nanoparticle‐based chelators, and modulators of ferroptosis and cuproptosis, hold promise. Among these, ferroptosis modulation stands out: small‐molecule inhibitors of lipid peroxidation (Ferrostatin‐1, Liproxstatin‐1) and ACSL4–GPX4 axis modulators have shown preclinical efficacy in Fe‐overload cardiomyopathy, neurodegeneration, and I/R injury, though clinical translation remains challenging. Targeting the FDX1–lipoylation pathway in cuproptosis may offer new avenues for Cu‐related cancers and Wilson's disease.

Despite these advances, substantial knowledge gaps remain. First, tissue‐specific susceptibility to metal toxicity is poorly understood: why the pituitary, heart, and liver are vulnerable to Fe overload, whereas the basal ganglia and liver are primary targets in Cu excess, remains an open question, with Fe's unique transport and storage kinetics likely playing a key role. Second, metal–metal interactions (synergistic or antagonistic) have been largely overlooked, especially Fe–Cu crosstalk via ceruloplasmin and hephestin. Third, noninvasive biomarkers for tissue Cu, Zn, and Ca burdens are lacking, whereas Fe has T_2_* MRI, a disparity that needs addressing. Fourth, long‐term safety and efficacy of ferroptosis and cuproptosis modulators require rigorous clinical trials. Fifth, the integration of metallomics with genomics, proteomics, and metabolomics, the emerging field of “metallo‐genomics,” promises personalized risk assessment, with Fe‐related genes (*HFE*, *HJV*, *TFR2*) providing early models for precision medicine. Future studies should prioritize sex‐ and age‐stratified analyses, as sex and developmental stage profoundly influence metal homeostasis. Finally, the therapeutic potential of modulating metal homeostasis in common diseases (neurodegeneration, cardiovascular disease, diabetes) where metal dysregulation is a secondary contributor deserves exploration, with Fe again serving as a prime candidate given its ubiquitous involvement.

In conclusion, metal ions are not merely dietary nutrients or toxicants; they are fundamental regulators of human health and disease. Fe, the most abundant and chemically versatile of them, stands as the prime exemplar, from the hemoglobin that sustains every breath to the ferritin that stores cellular Fe, from the Fenton chemistry that drives ferroptosis to the hepcidin that orchestrates systemic balance. Yet Cu, Zn, and Ca are equally woven into the fabric of life: Cu in mitochondrial respiration, Zn in genomic sculpting, and Ca in every heartbeat. Understanding their collective biology is not only a scientific imperative but a clinical necessity, offering tangible pathways to better diagnose, treat, and ultimately prevent the many diseases that arise when metal homeostasis goes awry. With Fe as the guiding thread, this integrated framework illuminates both the unity and diversity of metal biology in human health and disease.

Looking forward, several priority directions demand coordinated effort. First, multiomics integration, encompassing metallo‐genomics, epigenomics, and metabolomics, should systematically map the genetic and environmental determinants of metal homeostasis across populations, with sex‐ and age‐stratified analyses to capture modulatory effects. Second, validated noninvasive biomarkers for tissue‐specific Cu, Zn, and Ca burdens are urgently needed, analogous to T_2_* MRI for Fe, and could be complemented by circulating markers of ferroptosis (e.g., lipid peroxidation products) and cuproptosis (e.g., lipoylated protein adducts) to enable dynamic monitoring. Third, tissue‐targeted and stimuli‐responsive delivery systems such as nanoparticle‐based chelators or ferroptosis inhibitors, could overcome the narrow therapeutic window of current agents, while systematic exploration of metal–metal interactions (Fe–Cu crosstalk, Zn–Cu competition, Ca–Fe interplay) may reveal novel combination strategies. Fourth, clinical translation of ferroptosis and cuproptosis modulators requires proof‐of‐concept trials in well‐defined patient cohorts, with biomarker‐guided patient selection. Fifth, metal dysregulation in common nonclassical disorders, neurodegeneration, cardiovascular disease, diabetes, and chronic kidney disease, should be evaluated as a modifiable amplifier of pathology through targeted intervention trials. Sixth, environmental and public health dimensions, particularly in low‐ and middle‐income countries facing dual burdens of deficiency and toxicity, merit integrated monitoring and cost‐effective mitigation strategies. Ultimately, the convergence of these efforts toward precision metallotherapy, personalized by genetic predisposition, metal status, comorbidity, and exposure profile, will require artificial intelligence‐driven clinical decision support, transforming current knowledge into actionable, individualized care across the lifespan.

## Author Contributions

X. Dai conceptualized the insights and prepared the manuscript, figures, and tables. J.T. Li approved the insights and provided the financial support. All authors have read and approved the final manuscript.

## Funding

The authors have nothing to report.

## Ethics Statement

The authors have nothing to report.

## Conflicts of Interest

The authors declare no conflicts of interest.

## Data Availability

The authors have nothing to report.

## References

[1] L. Pauling and C. D. Coryell , “The Magnetic Properties and Structure of Hemoglobin, Oxyhemoglobin and Carbonmonoxyhemoglobin,” Proceedings of the National Academy of Sciences of the United States of America 22, no. 4 (April 1936): 210–216. 10.1073/pnas.22.4.210 PMC1076743 16577697

[2] D. Keilin and T. Mann , “Carbonic Anhydrase. Purification and Nature of the Enzyme,” Biochemical Journal 34, no. 8‐9 (September 1940): 1163–1176. 10.1042/bj0341163 PMC1265396 16747299

[3] M. W. Hentze , M. U. Muckenthaler , B. Galy , and C. Camaschella , “Two to Tango: Regulation of Mammalian Iron Metabolism,” Cell 142, no. 1 (2010): 24–38. 10.1016/j.cell.2010.06.028 20603012

[4] E. L. MacKenzie , K. Iwasaki , and Y. Tsuji , “Intracellular Iron Transport and Storage: From Molecular Mechanisms to Health Implications,” Antioxidants & Redox Signaling 10, no. 6 (June 2008): 997–1030. 10.1089/ars.2007.1893 PMC2932529 18327971

[5] F. Zhang , Y. Cheng , B. Xue , Y. Gao , Y. Liu , and C. Wang , “Metalnet2: An Enhanced Server for Predicting Metal‐Binding Sites in Proteomes,” National Science Review 11, no. 12 (December 2024): nwae391. 10.1093/nsr/nwae391 PMC11660930 39712664

[6] M. D. Peris‐Díaz , A. Orzeł , S. Wu , K. Mosna , P. E. Barran , and A. Krężel , “Combining Native Mass Spectrometry and Proteomics to Differentiate and Map the Metalloform Landscape in Metallothioneins,” Journal of Proteome Research 23, no. 8 (August 2024): 3626–3637. 10.1021/acs.jproteome.4c00271 PMC11301679 38993068

[7] K. Jomova and M. Valko , “Advances in Metal‐Induced Oxidative Stress and Human Disease,” Toxicology 283, no. 2‐3 (May 10 2011): 65–87. 10.1016/j.tox.2011.03.001 21414382

[8] K. Jomova , M. Makova , S. Y. Alomar , et al., “Essential Metals in Health and Disease,” Chemico‐Biological Interactions (November 2022): 110173. 10.1016/j.cbi.2022.110173 36152810

[9] B. Chen , P. Yu , W. N. Chan , et al., “Cellular Zinc Metabolism and Zinc Signaling: From Biological Functions to Diseases and Therapeutic Targets,” Signal Transduction and Targeted Therapy 9, no. 1 (January 2024): 6. 10.1038/s41392-023-01679-y PMC10761908 38169461

[10] C. López‐Otín and G. Kroemer , “Hallmarks of Health,” Cell 184, no. 7 (April 2021): 1929–1939. 10.1016/j.cell.2021.03.033 33798440

[11] M. Valko , D. Leibfritz , J. Moncol , M. T. Cronin , M. Mazur , and J. Telser , “Free Radicals and Antioxidants in Normal Physiological Functions and Human Disease,” The International Journal of Biochemistry & Cell Biology 39, no. 1 (2007): 44–84. 10.1016/j.biocel.2006.07.001 16978905

[12] M. S. Razzaque and S. J. Wimalawansa , “Minerals and Human Health: From Deficiency to Toxicity,” Nutrients 17, no. 3 (January 2025): 454. 10.3390/nu17030454 PMC11820417 39940312

[13] E. I. Obeagu , “Iron Homeostasis and Health: Understanding Its Role Beyond Blood Health—A Narrative Review,” Annals of Medicine & Surgery 87, no. 6 (June 2025): 3362–3371. 10.1097/MS9.0000000000003100 PMC12140690 40486647

[14] M. Auerbach , T. G. DeLoughery , and J. S. Tirnauer , “Iron Deficiency in Adults,” Jama 333, no. 20 (May 2025): 1813. 10.1001/jama.2025.0452 40159291

[15] C. Roidos , C. A. Batakoias , E. N. Symeonidis , et al., “Beta‐Thalassemia and Male Infertility: Unraveling the Oxidative Stress Connection—An Up‐to‐Date Review,” Diagnostics (Basel) 14, no. 24 (December 2024): 2789. 10.3390/diagnostics14242789 PMC11727083 39767150

[16] A. Polizzi , “Recent Advances in Research on Iron Metabolism, Ferritin, and Hepcidin,” International Journal of Molecular Sciences 27, no. 2 (January 2026): 906. 10.3390/ijms27020906 PMC12841351 41596552

[17] M. Tang , J. Zhang , and M. Yu , “Advances in Copper Homeostasis and Copper Dysregulation in Diseases,” Journal of Trace Elements in Medicine and Biology 91 (October 2025): 127727. 10.1016/j.jtemb.2025.127727 40902208

[18] A. Binesh and K. Venkatachalam , “Copper in Human Health and Disease: A Comprehensive Review,” Journal of Biochemical and Molecular Toxicology 38, no. 11 (November 2024): e70052. 10.1002/jbt.70052 39503199

[19] A. Hussain , W. Jiang , X. Wang , et al., “Mechanistic Impact of Zinc Deficiency in Human Development,” Frontiers in Nutrition 9 (2022): 717064. 10.3389/fnut.2022.717064 PMC8959901 35356730

[20] A. S. Prasad , “Lessons Learned From Experimental Human Model of Zinc Deficiency,” Journal of Immunology Research 2020 (2020): 9207279. 10.1155/2020/9207279 PMC7199614 32411807

[21] L. I. Stiles , K. Ferrao , and K. J. Mehta , “Role of Zinc in Health and Disease,” Clinical and Experimental Medicine 24, no. 1 (February 2024): 38. 10.1007/s10238-024-01302-6 PMC10874324 38367035

[22] D. Matuz‐Mares , M. Gonzalez‐Andrade , M. G. Araiza‐Villanueva , M. M. Vilchis‐Landeros , and H. Vazquez‐Meza , “Mitochondrial Calcium: Effects of Its Imbalance in Disease,” Antioxidants (Basel) 11, no. 5 (April 2022): 801. 10.3390/antiox11050801 PMC9138001 35624667

[23] M. S. Cooper and N. J. Gittoes , “Diagnosis and Management of Hypocalcaemia,” Bmj 336, no. 7656 (June 2008): 1298–1302. 10.1136/bmj.39582.589433.BE PMC2413335 18535072

[24] C. Liao , T.‐W. Peng , X.‐M. Li , et al., “Identification of Ferroptotic Genes and Phenotypes in Idiopathic Nonobstructive Azoospermia,” Systems Biology in Reproductive Medicine 69, no. 6 (December 2023): 410–422. 10.1080/19396368.2023.2257352 37782778

[25] E. Moretti , C. Signorini , L. Liguori , et al., “Evaluation of Known Markers of Ferroptosis in Semen of Patients With Different Reproductive Pathologies and Fertile Men,” Cells 13, no. 17 (September 2024): 1490. 10.3390/cells13171490 PMC11394366 39273059

[26] P. Tsvetkov , S. Coy , B. Petrova , et al., “Copper Induces Cell Death by Targeting Lipoylated TCA Cycle Proteins,” Science 375, no. 6586 (March2022): 1254–1261. 10.1126/science.abf0529 PMC9273333 35298263

[27] S. J. Dixon , K. M. Lemberg , M. R. Lamprecht , et al., “Ferroptosis: An Iron‐Dependent Form of Nonapoptotic Cell Death,” Cell 149, no. 5 (May 2012): 1060–1072. 10.1016/j.cell.2012.03.042 PMC3367386 22632970

[28] Y. Yang , J. Wu , L. Wang , G. Ji , and Y. Dang , “Copper Homeostasis and Cuproptosis in Health and Disease,” MedComm 5, no. 10 (October 2024): e724. 10.1002/mco2.724 PMC11406047 39290254

[29] B. R. Stockwell , J. P. Friedmann Angeli , H. Bayir , et al., “Ferroptosis: A Regulated Cell Death Nexus Linking Metabolism, Redox Biology, and Disease,” Cell 171, no. 2 (October 2017): 273–285. 10.1016/j.cell.2017.09.021 PMC5685180 28985560

[30] T. Rolić , M. Yazdani , S. Mandić , and S. Distante , “Iron Metabolism, Calcium, Magnesium and Trace Elements: A Review,” Biological Trace Element Research 203, no. 4 (April 2025): 2216–2225. 10.1007/s12011-024-04289-z PMC11920315 38969940

[31] Z. I. Malik , M. U. Ghafoor , S. Shah , J. Abid , U. Farooq , and A. M. R. Ahmad , “Unlocking Iron: Nutritional Origins, Metabolic Pathways, and Systemic Significance,” Frontiers in Nutrition 12 (2025): 1637316. 10.3389/fnut.2025.1637316 PMC12380693 40880741

[32] T. Silva , G. M. M. Vieira , L. T. S. Fraga , W. D. Fernandes , E. N. Sakane , and S. S. Maeda , “Supplements for Bone Health,” Arch Endocrinol Metab 70, no. Spe1 (December 2025): e20250374. 10.20945/2359-4292-2025-0374 PMC12714311 41370665

[33] P. Trumbo , A. A. Yates , S. Schlicker , and M. Poos , “Dietary Reference Intakes,” Journal of the American Dietetic Association 101, no. 3 (March 2001): 294–301. 10.1016/S0002-8223(01)00078-5 11269606

[34] T. Y. Wu , W. Gruissem , and N. K. Bhullar , “Targeting Intracellular Transport Combined With Efficient Uptake and Storage Significantly Increases Grain Iron and Zinc Levels in Rice,” Plant Biotechnology Journal 17, no. 1 (January 2019): 9–20. 10.1111/pbi.12943 PMC6330537 29734523

[35] C. Berndt , H. Alborzinia , V. S. Amen , et al., “Ferroptosis in Health and Disease,” Redox Biology 75 (September 2024): 103211. 10.1016/j.redox.2024.103211 PMC11253697 38908072

[36] S. K. Das and G. Y. Oudit , “Voltage‐Gated C a^2+^ Channels as Key Mediators of Iron‐Transport and Iron‐Overload Cardiomyopathy: L ‐Type vs. T ‐Type C a^+^ Channels,” European Journal of Haematology 88, no. 6 (June 2012): 476–477. 10.1111/j.1600-0609.2012.01782.x 22420502

[37] S. Kumfu , S. C. Chattipakorn , S. Fucharoen , and N. Chattipakorn , “Dual T‐Type and L‐Type Calcium Channel Blocker Exerts Beneficial Effects in Attenuating Cardiovascular Dysfunction in Iron‐Overloaded Thalassaemic Mice,” Experimental Physiology 101, no. 4 (April 2016): 521–539. 10.1113/EP085517 26824522

[38] A. Syed and J. A. Tainer , “Charge Transport Communication Through DNA by Protein Fe–S Clusters: How Far Is Not Too Far?,” ACS Central Science 5, no. 1 (January 23 2019): 7–9. 10.1021/acscentsci.8b00909 PMC6346388 30693320

[39] J. O. Fuss , C. L. Tsai , J. P. Ishida , and J. A. Tainer , “Emerging Critical Roles of Fe–S Clusters in DNA Replication and Repair,” Biochimica et Biophysica Acta (BBA)—Molecular Cell Research 1853, no. 6 (June 2015): 1253–1271. 10.1016/j.bbamcr.2015.01.018 PMC4576882 25655665

[40] L. Cantamessa , M. Asperti , M. Gryzik , et al., “Didox, a Ribonucleotide Reductase Inhibitor With Iron Chelator Properties, Counteracts the in Vitro and in Vivo Growth of Rhabdomyosarcoma Cells,” Biochemical Pharmacology 242, no. Pt 2 (December 2025): 117312. 10.1016/j.bcp.2025.117312 40925467

[41] K. Gaur , S. C. Pérez Otero , J. A. Benjamín‐Rivera , et al., “Iron Chelator Transmetalative Approach to Inhibit Human Ribonucleotide Reductase,” JACS Au 1, no. 6 (June 28 2021): 865–878. 10.1021/jacsau.1c00078 PMC8243325 34240081

[42] L. Pacoste , R. Kumar , V. Srinivas , et al., “Tracking the Redox Reaction of the Iron Enzyme Ribonucleotide Reductase Using Continuous Serialed and Sfx,” Structure 34, no. 6 (June 4 2026): 901–914.e6. 10.1016/j.str.2026.03.006 PMC13166098 42097140

[43] F. P. Guengerich , “Introduction: Metals in Biology: Α‐Ketoglutarate/Iron‐Dependent Dioxygenases,” Journal of Biological Chemistry 290, no. 34 (August 2015): 20700–20701. 10.1074/jbc.R115.675652 PMC4543631 26152720

[44] Y. U.‐I. Tsukada , J. Fang , H. Erdjument‐Bromage , et al., “Histone Demethylation by a Family of Jmjc Domain‐Containing Proteins,” Nature 439, no. 7078 (February 2006): 811–816. 10.1038/nature04433 16362057

[45] R. J. Klose , E. M. Kallin , and Y. Zhang , “Jmjc‐Domain‐Containing Proteins and Histone Demethylation,” Nature Reviews Genetics 7, no. 9 (September 2006): 715–727. 10.1038/nrg1945 16983801

[46] F. Carlomagno , M. Tenuta , A. Sansone , et al., “Iron Overload Disorders in Adults: A Comprehensive Review of Gonadal Function, Reproductive, and Sexual Health,” Human Reproduction Update 32, no. 5 (May 2026): 663–690. 10.1093/humupd/dmag010 PMC13538717 42101252

[47] H. M. Sakhr , M. H. Hassan , E. A. Abdallah , A. M. Ewis , M. H. Mohamed , and S. G. Rizk , “Thyroid Peroxidase Gene Variants and Susceptibility to Congenital Hypothyroidism and Autoimmune Thyroid Disease Among egyptian Pediatric Cohort,” Clinical and Experimental Pediatrics 69, no. 6 (June 2026): 486–496. 10.3345/cep.2025.02957 PMC13244060 41927052

[48] S. Y. Hess , M. B. Zimmermann , R. F. Hurrell , M. Arnold , and W. Langhans , “Iron Deficiency Anemia Reduces Thyroid Peroxidase Activity in Rats,” The Journal of Nutrition 132, no. 7 (July 2002): 1951–1955. 10.1093/jn/132.7.1951 12097675

[49] H. M. Saad , E. A. Salem , O. Elhussieny , T. S. Waheeb , and A. E. Elsayed , “Crosstalk Between Ferroptosis and Mirna in Type 2 Diabetes Mellitus and Possible Therapeutic Targeting,” European Journal of Medical Research 30, no. 1 (October 2025): 982. 10.1186/s40001-025-03178-y PMC12529866 41094555

[50] X. Li , F. Wang , D. Zhao , Y. Xu , S. Yan , and B. Li , “Multi‐Omics Integration Identifies the CBS—CTH—GSH Axis as a Critical Regulator of β‐Cell Ferroptosis in Type 2 Diabetes,” The FASEB Journal 40, no. 8 (April 2026): e71792. 10.1096/fj.202600188R 41996204

[51] A. Marku , A. Galli , P. Marciani , N. Dule , C. Perego , and M. Castagna , “Iron Metabolism in Pancreatic Beta‐Cell Function and Dysfunction,” Cells 10, no. 11 (October 2021): 2841. 10.3390/cells10112841 PMC8616520 34831062

[52] W. Sha , F. Hu , Y. Xi , Y. Chu , and S. Bu , “Mechanism of Ferroptosis and Its Role in Type 2 Diabetes Mellitus,” Journal of Diabetes Research 2021 (2021): 1–10. 10.1155/2021/9999612 PMC8257355 34258295

[53] A. Saini and N. Nair , “Trace Element Copper in Male and Female Reproduction: A Review,” Bulletin of Environment, Pharmacology and Life Sciences 11, no. 9 (2022): 15–22.

[54] S. Herman , P. ł. Lipiński , M. Ogórek , et al., “Molecular Regulation of Copper Homeostasis in the Male Gonad During the Process of Spermatogenesis,” International Journal of Molecular Sciences 21, no. 23 (November 2020): 9053. 10.3390/ijms21239053 PMC7730223 33260507

[55] B. H. Bin , S. Hojyo , T. Ryong Lee , and T. Fukada , “Spondylocheirodysplastic Ehlers‐Danlos Syndrome (SCD‐EDS) and the Mutant Zinc Transporter ZIP13,” Rare Diseases 2, no. 1 (2014): e974982. 10.4161/21675511.2014.974982 PMC4755239 26942106

[56] C. R. Ferreira and W. A. Gahl , “Disorders of Metal Metabolism,” Transl Sci Rare Dis 2, no. 3‐4 (December 2017): 101–139. 10.3233/TRD-170015 PMC5764069 29354481

[57] A. Avan , A. Członkowska , S. Gaskin , A. Granzotto , S. L. Sensi , and T. U. Hoogenraad , “The Role of Zinc in the Treatment of Wilson's Disease,” International Journal of Molecular Sciences 23, no. 16 (August 2022): 9316. 10.3390/ijms23169316 PMC9409413 36012580

[58] G. Mantovani , M. Bastepe , D. Monk , et al., “Diagnosis and Management of Pseudohypoparathyroidism and Related Disorders: First International Consensus Statement,” Nature Reviews Endocrinology 14, no. 8 (August 2018): 476–500. 10.1038/s41574-018-0042-0 PMC6541219 29959430

[59] N. Panay and R. A. Anderson , “Evidence‐Based Guideline: Premature Ovarian Insufficiency,” Fertility and Sterility 123, no. 2 (February 2025): 221–236. 10.1016/j.fertnstert.2024.11.007 39652037

[60] I. Lambrinoudaki , S. A. Paschou , M. A. Lumsden , et al., “Premature Ovarian Insufficiency: A Toolkit for the Primary Care Physician,” Maturitas 147 (May 2021): 53–63. 10.1016/j.maturitas.2020.11.004 33451805

[61] T. Kambe , T. Tsuji , A. Hashimoto , and N. Itsumura , “The Physiological, Biochemical, and Molecular Roles of Zinc Transporters in Zinc Homeostasis and Metabolism,” Physiological Reviews 95, no. 3 (July 2015): 749–784. 10.1152/physrev.00035.2014 26084690

[62] R. Squitti , A. Pal , M. Picozza , et al., “Zinc Therapy in Early alzheimer's Disease: Safety and Potential Therapeutic Efficacy,” Biomolecules 10, no. 8 (August 2020): 1164. 10.3390/biom10081164 PMC7466035 32784855

[63] J. R. Evans and J. G. Lawrenson , “Antioxidant Vitamin and Mineral Supplements for Slowing the Progression of Age‐related Macular Degeneration,” Cochrane Database of Systematic Reviews 9, no. 9 (September 2023): CD000254. 10.1002/14651858.CD000254.pub5 PMC10498493 37702300

[64] Age‐Related Eye Disease Study Research G , A Randomized, Placebo‐Controlled, Clinical Trial of High‐Dose Supplementation with Vitamins C and E and Beta Carotene for Age‐Related Cataract and Vision Loss, Archives of Ophthalmology Oct 2001;119(10):1439. 10.1001/archopht.119.10.1439 PMC1472812 11594943

[65] E. Y. Chew , T. E. Clemons , E. Agrón , et al., “Long‐Term Effects of Vitamins C and E, β‐Carotene, and Zinc on Age‐Related Macular Degeneration,” Ophthalmology 120, no. 8 (August 2013): 1604–1611.e4. 10.1016/j.ophtha.2013.01.021 PMC3728272 23582353

[66] S. Hagmeyer , A. K. Sauer , and A. M. Grabrucker , “Prospects of Zinc Supplementation in Autism Spectrum Disorders and Shankopathies Such as phelan Mcdermid Syndrome,” Frontiers in Synaptic Neuroscience 10 (2018): 11. 10.3389/fnsyn.2018.00011 PMC5974951 29875651

[67] K. Lee , Z. Mills , P. Cheung , J. E. Cheyne , and J. M. Montgomery , “The Role of Zinc and NMDA Receptors in Autism Spectrum Disorders,” Pharmaceuticals 16, no. 1 (December 2022): 1. 10.3390/ph16010001 PMC9866730 36678498

[68] M. Frega , M. Selten , B. Mossink , et al., “Distinct Pathogenic Genes Causing Intellectual Disability and Autism Exhibit a Common Neuronal Network Hyperactivity Phenotype,” Cell Reports 30, no. 1 (January 2020): 173–186.e6. 10.1016/j.celrep.2019.12.002 31914384

[69] H. Yasuda , K. Yoshida , Y. Yasuda , and T. Tsutsui , “Infantile Zinc Deficiency: Association With Autism Spectrum Disorders,” Scientific Reports 1 (2011): 129. 10.1038/srep00129 PMC3216610 22355646

[70] M. L. Ackland and A. Michalczyk , “Zinc Deficiency and Its Inherited Disorders—A Review,” Genes & Nutrition 1, no. 1 (March 2006): 41–49. 10.1007/BF02829935 PMC3454812 18850219

[71] G. K. Andrews , “Regulation and Function of ZIP4 , the Acrodermatitis Enteropathica Gene,” Biochemical Society Transactions 36, no. Pt 6 (December 2008): 1242–1246. 10.1042/BST0361242 PMC2634863 19021533

[72] Y.‐E. Cho , R.‐A. R. Lomeda , S.‐H. Ryu , et al., “Zinc Deficiency Negatively Affects Alkaline Phosphatase and the Concentration of Ca, Mg and P in Rats,” Nutrition Research and Practice 1, no. 2 (2007): 113. 10.4162/nrp.2007.1.2.113 PMC2882585 20535396

[73] G. Morciano , N. Naumova , P. Koprowski , et al., “The Mitochondrial Permeability Transition Pore: An Evolving Concept Critical for Cell Life and Death,” Biological Reviews 96, no. 6 (December 2021): 2489–2521. 10.1111/brv.12764 34155777

[74] G. Gherardi , A. Weiser , F. Bermont , et al., “Mitochondrial Calcium Uptake Declines During Aging and Is Directly Activated by Oleuropein to Boost Energy Metabolism and Skeletal Muscle Performance,” Cell Metabolism 37, no. 2 (February 2025): 477–495.e11. 10.1016/j.cmet.2024.10.021 39603237

[75] K. Mallilankaraman , P. Doonan , C. Cárdenas , et al., “MICU1 Is an Essential Gatekeeper for MCU‐Mediated Mitochondrial Ca2+ Uptake That Regulates Cell Survival,” Cell 151, no. 3 (October 2012): 630–644. 10.1016/j.cell.2012.10.011 PMC3486697 23101630

[76] K. J. Kamer and V. K. Mootha , “MICU1 and MICU2 Play Nonredundant Roles in the Regulation of the Mitochondrial Calcium Uniporter,” The EMBO Reports 15, no. 3 (March 2014): 299–307. 10.1002/embr.201337946 PMC3989696 24503055

[77] J. Ji , L. Su , and Z. Liu , “Critical Role of Calpain in Inflammation,” Biomedical Reports 5, no. 6 (December 2016): 647–652. 10.3892/br.2016.785 PMC5228304 28101338

[78] A. G. Yakovlev , G. Wang , B. A. Stoica , et al., “A Role of the Ca2+/Mg2+‐Dependent Endonuclease in Apoptosis and Its Inhibition by Poly(ADP‐Ribose) Polymerase,” Journal of Biological Chemistry 275, no. 28 (July 2000): 21302–21308. 10.1074/jbc.M001087200 10807908

[79] M. Bastepe and R. C. Gensure , “Hypoparathyroidism and Pseudohypoparathyroidism,” in Endotext, ed. Feingold KR , Adler RA , and Ahmed SF , (2000). 25905388

[80] P. Gaschen , M. H. Almekdash , and A. Peiris , “Diagnosis and Management of Hyperparathyroidism,” JAMA Internal Medicine 179, no. 12 (December 2019): 1732. 10.1001/jamainternmed.2019.5129 31790529

[81] B. L. Clarke , E. M. Brown , M. T. Collins , et al., “Epidemiology and Diagnosis of Hypoparathyroidism,” The Journal of Clinical Endocrinology & Metabolism 101, no. 6 (June 2016): 2284–2299. 10.1210/jc.2015-3908 PMC5393595 26943720

[82] S. R. Kumar , K. G. S. Kumar , and R. Gayathri , “Hypomagnesemia in Patients With Type 2 Diabetes Mellitus,” Journal of the Association of Physicians of India 72, no. 7 (July 2024): 25–28. 10.59556/japi.72.0410 38990583

[83] A. Jackson and D. Namarika , “Chronic Symptomatic Hypocalcaemia—Difficulties in Diagnosis and Treatment in Rural Africa,” Tropical Doctor 40, no. 4 (October 2010): 251–252. 10.1258/td.2010.100166 20729263

[84] M. Kosugi , R. Takezawa , S. Shiota , S. Inokuchi , H. Takaya , and M. Yamauchi , “Management of Iron Deficiency Anemia and the Role of Intravenous Iron Supplementation in Patients Undergoing Cancer Chemotherapy: A Real‐World Retrospective Study in Japan,” International Journal of Clinical Oncology 31, no. 7(June 2026): 1408–1417. 10.1007/s10147-026-03057-4 PMC13303690 42228300

[85] J. V. Facco , D. S. Krause , and S. T. Olalla Saad , “Mechanisms of Thrombocytosis in Iron‐Deficiency Anemia,” European Journal of Haematology 117, no. 4 (May 2026): 728–738. 10.1111/ejh.70211 PMC13542968 42097980

[86] T. Ma , J. Du , Y. Zhang , Y. Wang , B. Wang , and T. Zhang , “GPX4‐Independent Ferroptosis—A New Strategy in Disease's Therapy,” Cell Death Discovery 8, no. 1 (October 2022): 434. 10.1038/s41420-022-01212-0 PMC9617873 36309489

[87] M. Musumeci , S. Maccari , A. Massimi , et al., “Iron Excretion in Iron Dextran‐Overloaded Mice,” Blood Transfus 12, no. 4 (October 2014): 485–490. 10.2450/2014.0288-13 PMC4212028 24960657

[88] E. M. Rossi , V. B. Marques , D. D. E. O. Nunes , et al., “Acute Iron Overload Leads to Hypothalamic‐Pituitary‐Gonadal Axis Abnormalities in Female Rats,” Toxicology Letters 240, no. 1 (January 5 2016): 196–213. 10.1016/j.toxlet.2015.10.027 26536400

[89] Y. Xie , C. Li , X. Dong , B. Wang , J. Qin , and H. Lv , “Natural Products as Modulators of Iron Metabolism and Ferroptosis in Diabetes and Its Complications,” Nutrients 17, no. 16 (August 2025): 2714. 10.3390/nu17162714 PMC12389314 40871742

[90] R. Mao , D. Yin , W. Lv , et al., “Iron: A Double‐Edged Sword of Female Reproductive Health,” Journal of Trace Elements in Medicine and Biology 93 (February 2026): 127808. 10.1016/j.jtemb.2025.127808 41406609

[91] C. M. Nouraee , W. H. Swain , D. M. Harmon , P. S. Kamath , M. M. Redfield , and J. C. Olson , “Rapid Onset of Iron Overload Cardiomyopathy in Cirrhosis,” JACC: Case Reports (May 2026): 108338. 10.1016/j.jaccas.2026.108338 PMC13309694 42126381

[92] S. Dong , X. Wang , S. An , Y. Hu , J. Xiao , and A. Jiang , “Assessing Causal Relationships Between Iron Level, Disorders of Iron Metabolism, and Hepatocellular Carcinoma: A Mendelian Randomisation Study: The First Author,” Discov Oncol 17, no. 1 (February 2026): 388. 10.1007/s12672-025-03086-x PMC12961066 41632391

[93] C. Y. Wang and J. L. Babitt , “Liver Iron Sensing and Body Iron Homeostasis,” Blood 133, no. 1 (January 2019): 18–29. 10.1182/blood-2018-06-815894 PMC6318427 30401708

[94] B. A. Ansharullah , H. Sutanto , and P. Z. Romadhon , “Thalassemia and Iron Overload Cardiomyopathy: Pathophysiological Insights, Clinical Implications, and Management Strategies,” Current Problems in Cardiology 50, no. 1 (January 2025): 102911. 10.1016/j.cpcardiol.2024.102911 39477176

[95] M. H. El‐Samahy , A. A. Tantawy , A. A. Adly , A. A. Abdelmaksoud , E. A. Ismail , and N. Y. Salah , “Evaluation of Continuous Glucose Monitoring System for Detection of Alterations in Glucose Homeostasis in Pediatric Patients With β‐Thalassemia Major,” Pediatric Diabetes 20, no. 1 (February 2019): 65–72. 10.1111/pedi.12793 30378745

[96] P. Evangelidis , T. M. Venou , B. Fani , E. Vlachaki , and E. Gavriilaki , on behalf of theInternational Hemoglobinopathy Research N , “Endocrinopathies in Hemoglobinopathies: What Is the Role of Iron?,” International Journal of Molecular Sciences 24, no. 22 (November 2023): 16263. 10.3390/ijms242216263 PMC10671246 38003451

[97] R. Huo , Y. He , C. Zhao , X. J. Guo , M. Lin , and J. H. Sha , “Identification of Human Spermatogenesis‐Related Proteins by Comparative Proteomic Analysis: A Preliminary Study,” Fertility and Sterility 90, no. 4 (October 2008): 1109–1118. 10.1016/j.fertnstert.2007.07.1342 18068167

[98] P. Hedera , A. Peltier , J. K. Fink , S. Wilcock , Z. London , and G. J. Brewer , “Myelopolyneuropathy and Pancytopenia Due to Copper Deficiency and High Zinc Levels of Unknown Origin II. The Denture Cream Is a Primary Source of Excessive Zinc,” Neurotoxicology 30, no. 6 (November 2009): 996–999. 10.1016/j.neuro.2009.08.008 19732792

[99] P. Hedera , J. K. Fink , P. L. Bockenstedt , and G. J. Brewer , “Myelopolyneuropathy and Pancytopenia Due to Copper Deficiency and High Zinc Levels of Unknown Origin,” Archives of Neurology 60, no. 9 (September 2003): 1303–1306. 10.1001/archneur.60.9.1303 12975299

[100] M. T. T. Silva , “Copper Deficiency Myelopathy Induced by Excessive Zinc Supplementation,” Practical Neurology 26, no. 2 (March 2026): 178–179. 10.1136/pn-2025-004785 40866286

[101] R. Petruzzelli , E. V. Polishchuk , and R. S. Polishchuk , “Copper in Human Health and Disease: Insights From Inherited Disorders,” Physiology (Bethesda) 41, no. 3 (May 2026): 0. 10.1152/physiol.00032.2025 PMC7618482 41213165

[102] S. Liu , Y. Chang , J. Li , et al., “Copper and Cuproptosis: From Homeostasis to Pathogenesis,” Chemico‐Biological Interactions 431 (May 2026): 112007. 10.1016/j.cbi.2026.112007 41794315

[103] M. T. Schulz and L. Rink , “Zinc Deficiency as Possible Link Between Immunosenescence and Age‐Related Diseases,” Immunity & Ageing 22, no. 1 (May 2025): 19. 10.1186/s12979-025-00511-1 PMC12087153 40390089

[104] R. Sugimoto , L. Lee , Y. Tanaka , et al., “Zinc Deficiency as a General Feature of Cancer: A Review of the Literature,” Biological Trace Element Research 202, no. 5 (May 2024): 1937–1947. 10.1007/s12011-023-03818-6 PMC10955002 37658952

[105] M. Maywald and L. Rink , “Zinc Deficiency and Zinc Supplementation in Allergic Diseases,” Biomolecules 14, no. 7 (July 2024): 863. 10.3390/biom14070863 PMC11274920 39062576

[106] A. Sivakumar , R. K. Vageshappa , and R. Kumari , “Acrodermatitis Enteropathica,” JAMA Dermatology 160, no. 1 (January 2024): 102. 10.1001/jamadermatol.2023.4147 37938848

[107] M. Maares and H. Haase , “A Guide to human Zinc Absorption: General Overview and Recent Advances of in Vitro Intestinal Models,” Nutrients 12, no. 3 (March 2020): 762. 10.3390/nu12030762 PMC7146416 32183116

[108] W. Cui , N. Wang , M. Shi , X. Xu , and H. Jiang , “Acrodermatitis Enteropathica in the Pediatric Population: A Literature Review of Real‐World Studies,” Frontiers in Nutrition 12 (2025): 1590075. 10.3389/fnut.2025.1590075 PMC12202354 40584104

[109] A. A. George , A. K. Mishra , K. K. Sahu , and J. Sargent , “Acquired Acrodermatitis Enteropathica,” The American Journal of Medicine 134, no. 1 (January 2021): e2–e3. 10.1016/j.amjmed.2020.09.011 33058784

[110] Y. Golan and Y. G. Assaraf , “Genetic and Physiological Factors Affecting Human Milk Production and Composition,” Nutrients 12, no. 5 (May 2020): 1500. 10.3390/nu12051500 PMC7284811 32455695

[111] A. N. Rodichkin , A. M. Brown , P. Devabathini , et al., “Cerebellar Dysfunction in a Mouse Model of Childhood‐Onset Manganese‐Induced Dystonia Parkinsonism,” Neurobiology of Disease 216 (November 2025): 107115. 10.1016/j.nbd.2025.107115 PMC13060767 40992700

[112] K. Tuschl , E. Meyer , L. E. Valdivia , et al., “Mutations in SLC39A14 Disrupt Manganese Homeostasis and Cause Childhood‐Onset Parkinsonism–Dystonia,” Nature Communications 7 (May 2016): 11601. 10.1038/ncomms11601 PMC4894980 27231142

[113] H. Deng , X. Qiao , T. Xie , et al., “SLC‐30A9 Is Required for Zn^2+^ Homeostasis, Zn^2+^ Mobilization, and Mitochondrial Health,” Proceedings of the National Academy of Sciences of the United States of America 118, no. 35 (August 2021): e2023909118. 10.1073/pnas.2023909118 PMC8536367 34433664

[114] J. E. Stanton , S. Malijauskaite , K. McGourty , and A. M. Grabrucker , “The Metallome as a Link Between the “Omes” in Autism Spectrum Disorders,” Frontiers in Molecular Neuroscience 14 (2021): 695873. 10.3389/fnmol.2021.695873 PMC8289253 34290588

[115] Z. Zhou , W. G. Qiao , and J. Lin , “Pstpip1‐Associated Myeloid‐Related Proteinemia Inflammatory (PAMI) Syndrome: A Case of Relapsed Episodes of Gastrointestinal Inflammation and Literature Review,” Journal of Clinical Pathology 78, no. 2 (January 2025): 141–144. 10.1136/jcp-2024-209463 38749662

[116] X. Ye , Q. Zhou , P. Ren , W. Xiang , and L. Xiao , Neuropsychiatric Disease and Treatment 19 (2023): 1515–1530. 10.2147/NDT.S407731 PMC10327924 37424961

[117] M. K. Baum , S. Lai , S. Sales , J. B. Page , and A. Campa , “Randomized, Controlled Clinical Trial of Zinc Supplementation to Prevent Immunological Failure in HIV‐Infected Adults,” Clinical Infectious Diseases 50, no. 12 (June 2010): 1653–1660. 10.1086/652864 PMC2874106 20455705

[118] A. Tokuyama , E. Kanda , S. Itano , et al., “Effect of Zinc Deficiency on Chronic Kidney Disease Progression and Effect Modification by Hypoalbuminemia,” PLoS ONE 16, no. 5 (2021): e0251554. 10.1371/journal.pone.0251554 PMC8112700 33974681

[119] C. McClain , V. Vatsalya , and M. Cave , “Role of Zinc in the Development/Progression of Alcoholic Liver Disease,” Current Treatment Options in Gastroenterology 15, no. 2 (June 2017): 285–295. 10.1007/s11938-017-0132-4 PMC6206836 28447197

[120] M. Yesiladali , M. G. K. Yazici , E. Attar , and F. Kelestimur , “Differentiating Polycystic Ovary Syndrome From Adrenal Disorders,” Diagnostics (Basel) 12, no. 9 (August 2022): 2045. 10.3390/diagnostics12092045 PMC9498167 36140452

[121] I. Youssry , M. El‐Ghamrawy , E. R. Youness , N. A. Mohamed , and Y. M. M. Selim , “Zinc Level and Its Impact on the Phenotype of Sickle Cell Disease,” Annals of Hematology 104, no. 6 (June 2025): 3213–3221. 10.1007/s00277-025-06429-4 PMC12283855 40461893

[122] Y. Liang , X. Zhang , Y. Liu , et al., “Gata Zinc Finger Domain‐Containing Protein 2a (GATAD2A) Deficiency Reactivates Fetal Haemoglobin in Patients With Beta‐Thalassaemia Through Impaired Formation of Methyl‐Binding Domain Protein 2 (MBD2)‐Containing Nucleosome Remodelling and Deacetylation (NURD) Complex,” British Journal of Haematology 193, no. 6 (June 2021): 1220–1227. 10.1111/bjh.17511 33997955

[123] R. Prasad , B. Hawthorne , D. Durai , and I. McDowell , “Zinc in Denture Adhesive: A Rare Cause of Copper Deficiency in a Patient on Home Parenteral Nutrition,” BMJ Case Reports (October 2015): bcr2015211390. 10.1136/bcr-2015-211390 PMC4600814 26452740

[124] U. M. Agnew and T. L. Slesinger , “Zinc Toxicity,” in Statpearls (StatPearls Publishing, 2026). 32119435

[125] H. Schoofs , J. Schmit , and L. Rink , “Zinc Toxicity: Understanding the Limits,” Molecules 29, no. 13 (July 2024): 3130. 10.3390/molecules29133130 PMC11243279 38999082

[126] S. Munie and P. Pintavorn , “Erythropoietin‐Resistant Anemia Secondary to Zinc‐Induced Hypocupremia in a Hemodialysis Patient,” Case Reports in Nephrology and Dialysis 11, no. 2 (May‐August 2021): 167–175. 10.1159/000512612 PMC8299384 34327219

[127] F. Amisha , P. Saluja , N. Gautam , S. Biyani , and S. Kakadia , “Acquired Hyperzincaemia Due to Zinc‐Laden Denture Adhesives Leading to Hypocupraemia as a Cause of Neutropenia,” Eur J Case Rep Intern Med 8, no. 11 (2021): 002983. 10.12890/2021_002983 PMC8667996 34912740

[128] A. Duncan , I. Morrison , and S. Bryson , “Iatrogenic Copper Deficiency: Risks and Cautions With Zinc Prescribing,” British Journal of Clinical Pharmacology 89, no. 9 (September 2023): 2825–2829. 10.1111/bcp.15749 37070154

[129] N. Gupta and M. F. Carmichael , “Zinc‐induced Copper Deficiency as a Rare Cause of Neurological Deficit and Anemia,” Cureus 15, no. 8 (August 2023): e43856. 10.7759/cureus.43856 PMC10510946 37736439

[130] L. Liu , E. Tian , S. Quan , et al., “Adam17 as a Promising Therapeutic Target: From Structural Basis to Inhibitor Discovery in Human Diseases,” Frontiers in Pharmacology 16 (2025): 1640090. 10.3389/fphar.2025.1640090 PMC12491190 41050403

[131] E. Marcello , B. Borroni , S. Pelucchi , F. Gardoni , and M. Di Luca , “Adam10 as a Therapeutic Target for Brain Diseases: From Developmental Disorders to Alzheimer's Disease,” Expert Opinion on Therapeutic Targets 21, no. 11 (November 2017): 1017–1026. 10.1080/14728222.2017.1386176 28960088

[132] S. Lourenco Dos Santos , I. Petropoulos , and B. Friguet , “The Oxidized Protein Repair Enzymes Methionine Sulfoxide Reductases and Their Roles in Protecting Against Oxidative Stress, in Ageing and in Regulating Protein Function,” Antioxidants (Basel) 7, no. 12 (December 2018): 191. 10.3390/antiox7120191 PMC6316033 30545068

[133] J. Guo , L. He , T. Li , J. Yin , Y. Yin , and G. Guan , “Antioxidant and Anti‐Inflammatory Effects of Different Zinc Sources on Diquat‐Induced Oxidant Stress in a Piglet Model,” BioMed Research International 2020 (2020): 3464068. 10.1155/2020/3464068 PMC7115046 32280687

[134] M. Jarosz , M. Olbert , G. Wyszogrodzka , K. Młyniec , and T. Librowski , “Antioxidant and Anti‐Inflammatory Effects of Zinc. Zinc‐Dependent NF‐κB Signaling,” Inflammopharmacology 25, no. 1 (February 2017): 11–24. 10.1007/s10787-017-0309-4 PMC5306179 28083748

[135] K. Morishita , M. Kubota , Y. Katakura , et al., “The Progression of Aging in Klotho Mutant Mice Can be Modified by Dietary Phosphorus and Zinc,” The Journal of Nutrition 131, no. 12 (December 2001): 3182–3188. 10.1093/jn/131.12.3182 11739863

[136] M. Malavolta and K. Pabis , “Elevated Metallothionein Expression in Long‐Lived Species,” Aging 14, no. 1 (January 2022): 1–3. 10.18632/aging.203831 PMC8791206 35027505

[137] K. Pabis , Y. Chiari , C. Sala , et al., “Elevated Metallothionein Expression in Long‐Lived Species Mediates the Influence of Cadmium Accumulation on Aging,” Geroscience 43, no. 4 (August 2021): 1975–1993. 10.1007/s11357-021-00393-3 PMC8492847 34117600

[138] J. P. Bilezikian , A. Khan , J. T. Potts , and J. Sanders, Jr. , “Hypoparathyroidism in the Adult: Epidemiology, Diagnosis, Pathophysiology, Target‐Organ Involvement, Treatment, and Challenges for Future Research,” Journal of Bone and Mineral Research 26, no. 10 (October 2011): 2317–2337. 10.1002/jbmr.483 PMC3405491 21812031

[139] M. E. van der Meijs , D. H. Schweitzer , and H. Boom , “Palatoschisis, Schizophrenia and Hypocalcaemia: Phenotypic Expression of 22q11.2 Deletion Syndrome (digeorge syndrome) in an Adult,” Eur J Case Rep Intern Med 8, no. 4 (2021): 002411. 10.12890/2021_002411 PMC8112094 33987118

[140] G. Reyhanoglu , D. Madhavarapu , and A. Kafrouni , “Autoimmune Polyendocrine Syndromes: Recognizing the Overlooked Clusters of Endocrinopathies,” Cureus 17, no. 8 (August 2025): e89936. 10.7759/cureus.89936 PMC12425835 40951041

[141] B. Mayr , M. Glaudo , and C. Schöfl , “Activating Calcium‐sensing Receptor Mutations: Prospects for Future Treatment With Calcilytics,” Trends in Endocrinology & Metabolism 27, no. 9 (September 2016): 643–652. 10.1016/j.tem.2016.05.005 27339034

[142] A. Misrani , C. Ngwa , and F. Liu , “Mitochondrial Dysfunction in Alzheimer's Disease and Related Sex Differences,” Frontiers in Aging Neuroscience 18 (2026): 1761702. 10.3389/fnagi.2026.1761702 PMC13055547 41952873

[143] F. Du , Q. Yu , N. M. Kanaan , and S. S. Yan , “Mitochondrial Oxidative Stress Contributes to the Pathological Aggregation and Accumulation of Tau Oligomers in Alzheimer's Disease,” Human Molecular Genetics 31, no. 15 (August 2022): 2498–2507. 10.1093/hmg/ddab363 PMC9396941 35165721

[144] G. Zhou , Q. Ye , Y. Xu , et al., “Mitochondrial Calcium Uptake 3 Mitigates Cerebral Amyloid Angiopathy‐related Neuronal Death and Glial Inflammation by Reducing Mitochondrial Dysfunction,” International Immunopharmacology 117 (April 2023): 109614. 10.1016/j.intimp.2022.109614 36878048

[145] Y. I.‐Q. Yan , R. Zheng , Y. I. Liu , et al., “Parkin Regulates Microglial NLRP3 and Represses Neurodegeneration in Parkinson's Disease,” Aging Cell 22, no. 6 (June 2023): e13834. 10.1111/acel.13834 PMC10265164 37029500

[146] C. Ma , Y. Liu , S. Li , et al., “Microglial cGAS Drives Neuroinflammation in the MPTP Mouse Models of Parkinson's Disease,” CNS Neuroscience & Therapeutics 29, no. 7 (July 2023): 2018–2035. 10.1111/cns.14157 PMC10324349 36914567

[147] A. Rimessi , S. Marchi , S. Patergnani , and P. Pinton , “H‐RAS‐Driven Tumoral Maintenance Is Sustained Through Caveolin‐1‐Dependent Alterations in Calcium Signaling,” Oncogene 33, no. 18 (May 2014): 2329–2340. 10.1038/onc.2013.192 23728347

[148] Z. Wu , M. Zuo , L. Zeng , et al., “Oma1 Reprograms Metabolism Under Hypoxia to Promote Colorectal Cancer Development,” The EMBO Reports 22, no. 1 (January 2021): e50827. 10.15252/embr.202050827 PMC7788456 33314701

[149] N. Nielsen , K. Kondratska , T. Ruck , et al., “TRPC6 Channels Modulate the Response of Pancreatic Stellate Cells to Hypoxia,” Pflügers Archiv—European Journal of Physiology 469, no. 12 (December 2017): 1567–1577. 10.1007/s00424-017-2057-0 28849300

[150] Y. Li , B. O. Guo , Q. Xie , et al., “Stim1 Mediates Hypoxia‐Driven Hepatocarcinogenesis via Interaction With HIF‐1,” Cell Reports 12, no. 3 (July 2015): 388–395. 10.1016/j.celrep.2015.06.033 26166565

[151] M. Vig , C. Peinelt , A. Beck , et al., “CRACM1 Is a Plasma Membrane Protein Essential for Store‐Operated Ca^2+^ Entry,” Science 312, no. 5777 (May2006): 1220–1223. 10.1126/science.1127883 PMC5685805 16645049

[152] K. Salnikow , T. Kluz , M. Costa , et al., “The Regulation of Hypoxic Genes by Calcium Involves c‐Jun/AP‐1, Which Cooperates With Hypoxia‐Inducible Factor 1 in Response to Hypoxia,” Molecular and Cellular Biology 22, no. 6 (March 2002): 1734–1741. 10.1128/MCB.22.6.1734-1741.2002 PMC135615 11865053

[153] J. Rius , M. Guma , C. Schachtrup , et al., “NF‐κB Links Innate Immunity to the Hypoxic Response Through Transcriptional Regulation of HIF‐1α,” Nature 453, no. 7196 (June 2008): 807–811. 10.1038/nature06905 PMC2669289 18432192

[154] R. Deng , H.‐L. Zhang , J.‐H. Huang , et al., “MAPK1/3 Kinase‐Dependent ULK1 Degradation Attenuates Mitophagy and Promotes Breast Cancer Bone Metastasis,” Autophagy 17, no. 10 (October 2021): 3011–3029. 10.1080/15548627.2020.1850609 PMC8526010 33213267

[155] S. Missiroli , M. Perrone , R. Gafà , et al., “PML at Mitochondria‐Associated Membranes Governs a Trimeric Complex With NLRP3 and P2X7R That Modulates the Tumor Immune Microenvironment,” Cell Death & Differentiation 30, no. 2 (February 2023): 429–441. 10.1038/s41418-022-01095-9 PMC9713080 36450825

[156] A. Rimessi , V. Bezzerri , S. Patergnani , S. Marchi , G. Cabrini , and P. Pinton , “Mitochondrial Ca2+‐Dependent NLRP3 Activation Exacerbates the pseudomonas Aeruginosa‐Driven Inflammatory Response in Cystic Fibrosis,” Nature Communications 6 (February 2015): 6201. 10.1038/ncomms7201 25648527

[157] A. Rimessi , C. Pozzato , L. Carparelli , et al., “Pharmacological Modulation of Mitochondrial Calcium Uniporter Controls Lung Inflammation in Cystic Fibrosis,” Science Advances 6, no. 19 (May 2020): eaax9093. 10.1126/sciadv.aax9093 PMC7202873 32494695

[158] Y. B. Zhang , Y. H. Meng , S. Chang , R. Y. Zhang , and C. Shi , “High Fructose Causes Cardiac Hypertrophy via Mitochondrial Signaling Pathway,” American journal of translational research 8, no. 11 (2016): 4869–4880. PMC5126329 27904687

[159] M. L. Kleme , A. Sané , C. Garofalo , et al., “Cftr Deletion Confers Mitochondrial Dysfunction and Disrupts Lipid Homeostasis in Intestinal Epithelial Cells,” Nutrients 10, no. 7 (June 2018): 836. 10.3390/nu10070836 PMC6073936 29954133

[160] A. Rimessi , V. A. M. Vitto , S. Patergnani , and P. Pinton , “Update on Calcium Signaling in Cystic Fibrosis Lung Disease,” Frontiers in Pharmacology 12 (2021): 581645. 10.3389/fphar.2021.581645 PMC7990772 33776759

[161] G. Morciano , C. Giorgi , M. Bonora , et al., “Molecular Identity of the Mitochondrial Permeability Transition Pore and Its Role in Ischemia‐Reperfusion Injury,” Journal of Molecular and Cellular Cardiology 78 (January 2015): 142–153. 10.1016/j.yjmcc.2014.08.015 25172387

[162] G. Campo , G. Morciano , R. Pavasini , et al., “FO ATP Synthase C Subunit Serum Levels in Patients With ST‐Segment Elevation Myocardial Infarction: Preliminary Findings,” International Journal of Cardiology 221 (October 2016): 993–997. 10.1016/j.ijcard.2016.07.125 27441480

[163] G. Morciano and P. Pinton , “Modulation of Mitochondrial Permeability Transition Pores in Reperfusion Injury: Mechanisms and Therapeutic Approaches,” European Journal of Clinical Investigation 55, no. 1 (January 2025): e14331. 10.1111/eci.14331 PMC11628652 39387139

[164] G. Morciano , G. Pedriali , M. Bonora , et al., “A Naturally Occurring Mutation in ATP Synthase Subunit c Is Associated With Increased Damage Following Hypoxia/Reoxygenation in Stemi Patients,” Cell Reports 35, no. 2 (April 2021): 108983. 10.1016/j.celrep.2021.108983 33852870

[165] L. U. Sun , R. Leng , M. Liu , et al., “Endothelial MICU1 Protects Against Vascular Inflammation and Atherosclerosis by Inhibiting Mitochondrial Calcium Uptake,” Journal of Clinical Investigation 135, no. 7 (April 2025): e181928. 10.1172/JCI181928 PMC11957702 40166941

[166] A. G. Bick , H. Wakimoto , K. J. Kamer , et al., “Cardiovascular Homeostasis Dependence on MICU2, a Regulatory Subunit of the Mitochondrial Calcium Uniporter,” Proceedings of the National Academy of Sciences of the United States of America 114, no. 43 (October 2017): E9096–E9104. 10.1073/pnas.1711303114 PMC5664535 29073106

[167] S. Zhang , Y. Lv , X. Luo , et al., “Homocysteine Promotes Atherosclerosis Through Macrophage Pyroptosis via Endoplasmic Reticulum Stress and Calcium Disorder,” Molecular Medicine 29, no. 1 (June 2023): 73. 10.1186/s10020-023-00656-z PMC10262416 37308812

[168] Y. Li , X. Liu , Q. Zhang , et al., “Koumine Mediates the Generation of MTROS Through the IP3R1‐GRP75‐VDAC1 Complex to Improve Citrinin Induced Intestinal Inflammation,” Phytomedicine 142 (July 2025): 156803. 10.1016/j.phymed.2025.156803 40311595

[169] B. O. Liu , A. S. Gulati , V. Cantillana , et al., “Irgm1‐Deficient Mice Exhibit Paneth Cell Abnormalities and Increased Susceptibility to Acute Intestinal Inflammation,” American Journal of Physiology‐Gastrointestinal and Liver Physiology 305, no. 8 (October 2013): G573–G584. 10.1152/ajpgi.00071.2013 PMC3798734 23989005

[170] A. Kabi , K. P. Nickerson , C. R. Homer , and C. McDonald , “Digesting the Genetics of Inflammatory Bowel Disease: Insights From Studies of Autophagy Risk Genes,” Inflammatory Bowel Diseases 18, no. 4 (April 2012): 782–792. 10.1002/ibd.21868 PMC3245781 21936032

[171] H. Zhang , L. Zheng , D. P. B. McGovern , et al., “Myeloid ATG16L1 Facilitates Host–Bacteria Interactions in Maintaining Intestinal Homeostasis,” The Journal of Immunology 198, no. 5 (March 2017): 2133–2146. 10.4049/jimmunol.1601293 PMC5322190 28130498

[172] J. Han , C. Yu , R. F. Souza , and A. L. Theiss , “Prohibitin 1 Modulates Mitochondrial Function of stat3,” Cellular Signalling 26, no. 10 (October 2014): 2086–2095. 10.1016/j.cellsig.2014.06.006 PMC4130792 24975845

[173] M. J. Lopez‐Armada , R. R. Riveiro‐Naveira , C. Vaamonde‐Garcia , and M. N. Valcarcel‐Ares , “Mitochondrial Dysfunction and the Inflammatory Response,” Mitochondrion 13, no. 2 (March 2013): 106–118. 10.1016/j.mito.2013.01.003 23333405

[174] U. Billmeier , W. Dieterich , M. F. Neurath , and R. Atreya , “Molecular Mechanism of Action of Anti‐Tumor Necrosis Factor Antibodies in Inflammatory Bowel Diseases,” World Journal of Gastroenterology 22, no. 42 (November 2016): 9300. 10.3748/wjg.v22.i42.9300 PMC5107694 27895418

[175] M. Bonora , E. De Marchi , S. Patergnani , et al., “Tumor Necrosis Factor‐α Impairs Oligodendroglial Differentiation Through a Mitochondria‐dependent Process,” Cell Death & Differentiation 21, no. 8 (August 2014): 1198–1208. 10.1038/cdd.2014.35 PMC4085526 24658399

[176] M. J. Manary , C. Hotz , N. F. Krebs , et al., “Zinc Homeostasis in Malawian Children Consuming a High‐Phytate, Maize‐Based Diet,” The American Journal of Clinical Nutrition 75, no. 6 (June 2002): 1057–1061. 10.1093/ajcn/75.6.1057 12036813

[177] M. Tachikawa , D. Keino , Y. Kimizuka , et al., “Copper Deficiency Presenting With Bicytopenia During Long‐Term Parenteral Nutrition for Short Bowel Syndrome: A Case Report and Literature Review,” Cureus 17, no. 5 (May 2025): e83688. 10.7759/cureus.83688 PMC12144283 40486299

[178] M. Locatelli and C. Farina , “Role of Copper in Central Nervous System Physiology and Pathology,” Neural Regeneration Research 20, no. 4 (April 2025): 1058–1068. 10.4103/NRR.NRR-D-24-00110 PMC11438321 38989937

[179] S. Kotnala , S. Tiwari , A. Nayak , et al., “Impact of Heavy Metal Toxicity on the Human Health and Environment,” Science of The Total Environment 987 (July 2025): 179785. 10.1016/j.scitotenv.2025.179785 40466229

[180] N. I. Liang , Z. Li , J. Sun , et al., “A Systematic Review on Heavy Metals in Indoor Air: Occurrence, Spatial Variation, and Health Risk,” Building and Environment 269 (2025): 112357.

[181] G. J. Udom , D. Iyaye , B. Oritsemuelebi , et al., “Public Health Concerns of Multifaceted Exposures to Metal and Metalloid Mixtures: A Systematic Review,” Environmental Monitoring and Assessment 197, no. 5 (April 2025): 502. 10.1007/s10661-025-13963-1 40172706

[182] K. Svensson , J. M. Lane , J. J. Chelonis , et al., “Developmental Pb Exposure Increases Rate of Forgetting on a Delayed Matching‐to‐Sample Task Among Mexican Children,” Science Advances 11, no. 28 (July 2025): eadq4495. 10.1126/sciadv.adq4495 PMC12239937 40632840

[183] T. Guo , M. L. Najafi , and J. Zhang , “A Systematic Review of Exposure to Toxic Elements and Neurocognitive Development in Children,” Ecotoxicology and Environmental Safety 291 (February 2025): 117792. 10.1016/j.ecoenv.2025.117792 39933231

[184] R. Su , Y. Jiang , W. Li , W. Ding , and L. Feng , “Effects of Prenatal Arsenic, Cadmium, and Manganese Exposure on Neurodevelopment in Children: A Systematic Review and Meta‐Analysis,” Medicina (Kaunas) 61, no. 7 (June 2025): 1143. 10.3390/medicina61071143 PMC12300850 40731773

[185] A. S. da Silva , R. M. S. Santos , P. G. De Marco , et al., “Neurodevelopmental Outcomes Associated With Early‐Life Exposure to Heavy Metals: A Systematic Review,” International Journal of Environmental Research and Public Health 22, no. 8 (August 2025): 1308. 10.3390/ijerph22081308 PMC12386800 40869893

[186] A. F. Pennington , M. R. Smith , S. O. Chuke , C. R. Cornwell , P. B. Allwood , and J. G. Courtney , “Effects of Blood Lead Levels <10 Microg/Dl in School‐Age Children and Adolescents: A Scoping Review,” Pediatrics 154, no. Suppl 2 (October 2024): e2024067808F. 10.1542/peds.2024-067808F PMC11610497 39352036

[187] R. Teschke , “Copper, Iron, Cadmium, and Arsenic, All Generated in the Universe: Elucidating Their Environmental Impact Risk on human Health Including Clinical Liver Injury,” International Journal of Molecular Sciences 25, no. 12 (June 2024): 6662. 10.3390/ijms25126662 PMC11203474 38928368

[188] M. C. Miletta , A. Bieri , K. Kernland , et al., “Transient Neonatal Zinc Deficiency Caused by a Heterozygous G87R Mutation in the Zinc Transporter ZnT‐2 (SLC30A2) Gene in the Mother Highlighting the Importance of Zn^2+^ for Normal Growth and Development,” International Journal of Endocrinology 2013 (2013): 259189. 10.1155/2013/259189 PMC3804372 24194756

[189] N. Charoenngam , P. Wattanachayakul , and M. Mannstadt , “Casrdb: A Publicly Accessible Comprehensive Database for Disease‐Associated Calcium‐sensing Receptor Variants,” The Journal of Clinical Endocrinology & Metabolism 110, no. 2 (January 2025): 297–302. 10.1210/clinem/dgae769 39484850

[190] F. M. Hannan , G. V. Walls , V. N. Babinsky , et al., “The Calcilytic Agent NPS 2143 Rectifies Hypocalcemia in a Mouse Model With an Activating Calcium‐Sensing Receptor (CASR) Mutation: Relevance to Autosomal Dominant Hypocalcemia Type 1 (ADH1),” Endocrinology 156, no. 9 (September 2015): 3114–3121. 10.1210/en.2015-1269 PMC4541614 26052899

[191] T. Grigore , Q. Leusink , F. Latta , et al., “Transient Hyperparathyroidism and Severe Intrauterine Osteopenia Linked to Novel Homozygous TRPV6 Deletion,” Clinical Endocrinology 105, no. 2 (March 2026): 197–204. 10.1111/cen.70134 PMC13326983 41885138

[192] Y. Suzuki , H. Sawada , T. Tokumasu , et al., “Novel TRPV6 Mutations in the Spectrum of Transient Neonatal Hyperparathyroidism,” The Journal of Physiological Sciences 70, no. 1 (July 2020): 33. 10.1186/s12576-020-00761-2 PMC10717230 32646367

[193] M. Hureaux , A. Molin , N. Jay , et al., “Prenatal Hyperechogenic Kidneys in Three Cases of Infantile Hypercalcemia Associated With SLC34A1 Mutations,” Pediatric Nephrology 33, no. 10 (October 2018): 1723–1729. 10.1007/s00467-018-3998-z 29959532

[194] M. Verjans , A. Hindryckx , K. Rosier , K. Devriendt , D. Mekahli , and D. Bockenhauer , “Antenatal Presentation and Early Postnatal Treatment of Infantile Hypercalcemia Type 2,” Pediatric Nephrology 39, no. 10 (October 2024): 2911–2913. 10.1007/s00467-024-06403-8 38753084

[195] T. Alelign and B. Petros , “Kidney Stone Disease: An Update on Current Concepts,” Advances in Urology 2018 (2018): 1–12. 10.1155/2018/3068365 PMC5817324 29515627

[196] A. A. N. Bhuiyan , N. Akter , T. Haq , M. Fariduddin , and S. Selim , “Recurrent Nephrolithiasis and Beyond: The Long Diagnostic Odyssey of a Case of CLDN16 Mutation,” Clinical Case Reports 13, no. 12 (December 2025): e71530. 10.1002/ccr3.71530 PMC12644926 41306405

[197] B. Carducci , E. C. Keats , and Z. A. Bhutta , “Zinc Supplementation for Improving Pregnancy and Infant Outcome,” Cochrane Database of Systematic Reviews 3, no. 3 (March 2021): CD000230. 10.1002/14651858.CD000230.pub6 PMC8094617 33724446

[198] V. Sangkhae , A. L. Fisher , T. Ganz , and E. Nemeth , “Iron Homeostasis During Pregnancy: Maternal, Placental, and Fetal Regulatory Mechanisms,” Annual Review of Nutrition 43 (August 2023): 279–300. 10.1146/annurev-nutr-061021-030404 PMC10723031 37253681

[199] L. Guan , Y. Wang , L. Lin , Y. Zou , and L. Qiu , “Variations in Blood Copper and Possible Mechanisms During Pregnancy,” Biological Trace Element Research 202, no. 2 (February 2024): 429–441. 10.1007/s12011-023-03716-x 37777692

[200] A. Or Koca , “Heavy Metals as Endocrine Disruptors,” Turk J Med Sci 56, no. 1 (2026): 7–14. 10.55730/1300-0144.6132 PMC12974275 41816752

[201] J. Jansen , W. Karges , and L. Rink , “Zinc and Diabetes–Clinical Links and Molecular Mechanisms,” Journal of Nutritional Biochemistry 20, no. 6 (June 2009): 399–417. 10.1016/j.jnutbio.2009.01.009 19442898

[202] M. H. Sohouli , F. Almuqayyid , A. Alfardous Alazm , et al., “A Comprehensive Review and Meta‐Regression Analysis of Randomized Controlled Trials Examining the Impact of Vitamin b12 Supplementation on Homocysteine Levels,” Nutrition Reviews 82, no. 6 (May 2024): 726–737. 10.1093/nutrit/nuad091 37495210

[203] Y. Yin , Z. Zhao , X. Wang , and Q. Wu , “Effects of Folic Acid With Vitamin b12/Vitamin b6 Intervention on Serum Homocysteine Metabolism and Complications in Patients With Type 2 Diabetes: A Systematic Review and Meta‐analysis of Randomized Controlled Trials,” Frontiers in Nutrition 12 (2025): 1701310. 10.3389/fnut.2025.1701310 PMC12698435 41393929

[204] T. G. St Pierre , P. R. Clark , W. Chua‐anusorn , et al., “Noninvasive Measurement and Imaging of Liver Iron Concentrations Using Proton Magnetic Resonance,” Blood 105, no. 2 (January 2005): 855–861. 10.1182/blood-2004-01-0177 15256427

[205] O. Bandmann , K. H. Weiss , and S. G. Kaler , “Wilson's Disease and Other Neurological Copper Disorders,” The Lancet Neurology 14, no. 1 (January 2015): 103–113. 10.1016/S1474-4422(14)70190-5 PMC4336199 25496901

[206] J. Walker , “Diagnosis and Management of Patients With Hypercalcaemia,” Nursing Older People 27, no. 4 (May 2015): 22–26. 10.7748/nop.27.4.22.e685 25924757

[207] W. Zhang , M. Doherty , T. Bardin , et al., “European League Against Rheumatism Recommendations for Calcium Pyrophosphate Deposition. Part i: Terminology and Diagnosis,” Annals of the Rheumatic Diseases 70, no. 4 (April 2011): 563–570. 10.1136/ard.2010.139105 21216817

[208] A. A. Khan , D. A. Hanley , R. Rizzoli , et al., “Primary Hyperparathyroidism: Review and Recommendations on Evaluation, Diagnosis, and Management. A Canadian and International Consensus,” Osteoporosis International 28, no. 1 (January 2017): 1–19. 10.1007/s00198-016-3716-2 PMC5206263 27613721

[209] R. V. Thakker , “Multiple Endocrine Neoplasia Type 1 (MEN1) and Type 4 (MEN4),” Molecular and Cellular Endocrinology 386, no. 1‐2 (April 2014): 2–15. 10.1016/j.mce.2013.08.002 PMC4082531 23933118

[210] O. M. Al‐Sanabra , M. A. Abbas , A. A. Hazàa , and W. J. Hazà , “Endocrine Complications in Patients With β‐Thalassemia Major Receiving Iron‐Chelation Therapy,” Therapeutic Advances in Endocrinology and Metabolism 16 (2025): 20420188251406554. 10.1177/20420188251406554 PMC12715153 41425691

[211] S. T. Singer , N. Sweeters , O. Vega , A. Higa , E. Vichinsky , and M. Cedars , “Fertility Potential in Thalassemia Major Women: Current Findings and Future Diagnostic Tools,” Annals of the New York Academy of Sciences 1202 (August 2010): 226–230. 10.1111/j.1749-6632.2010.05583.x PMC5074539 20712797

[212] S. T. Singer , E. P. Vichinsky , G. Gildengorin , J. van Disseldorp , M. Rosen , and M. I. Cedars , “Reproductive Capacity in Iron Overloaded Women With Thalassemia Major,” Blood 118, no. 10 (September 2011): 2878–2881. 10.1182/blood-2011-06-360271 PMC3172803 21757620

[213] K. Farmaki , I. Tzoumari , C. Pappa , G. Chouliaras , and V. Berdoukas , “Normalisation of Total Body Iron Load With Very Intensive Combined Chelation Reverses Cardiac and Endocrine Complications of Thalassaemia Major,” British Journal of Haematology 148, no. 3 (February 2010): 466–475. 10.1111/j.1365-2141.2009.07970.x 19912219

[214] N. Skordis and M. Toumba , “Bone Disease in Thalassaemia Major: Recent Advances in Pathogenesis and Clinical Aspects,” Pediatr Endocrinol Rev 8, no. Suppl 2 (March 2011): 300–306. 21705982

[215] G. Corona , D. G. Goulis , I. Huhtaniemi , et al., “European Academy of Andrology (EAA) Guidelines on Investigation, Treatment and Monitoring of Functional Hypogonadism in Males,” Andrology 8, no. 5 (September 2020): 970–987. 10.1111/andr.12770 32026626

[216] A. Gautier , F. Lainé , C. Massart , et al., “Liver Iron Overload Is Associated With Elevated SHBG Concentration and Moderate Hypogonadotrophic Hypogonadism in Dysmetabolic Men Without Genetic Haemochromatosis,” European Journal of Endocrinology 165, no. 2 (August 2011): 339–343. 10.1530/EJE-11-0215 21646287

[217] T. Cundy , A. Bomford , J. Butler , M. Wheeler , and R. Williams , “Hypogonadism and Sexual Dysfunction in Hemochromatosis: The Effects of Cirrhosis and Diabetes,” The Journal of Clinical Endocrinology & Metabolism 69, no. 1 (July 1989): 110–116. 10.1210/jcem-69-1-110 2732293

[218] S. De Vincentis , S. Evangelisti , B. Rossi , et al., “Hypoparathyroidism in Adults With Iron Overload Diseases (IOD): Evidence of a Subclinical Phenotype,” Endocrine 87, no. 3 (March 2025): 1257–1266. 10.1007/s12020-024-04124-4 39630327

[219] O. Fonseca , A. S. Ramos , L. T. S. Gomes , M. S. Gomes , and A. C. Moreira , “New Perspectives on Circulating Ferritin: Its Role in Health and Disease,” Molecules 28, no. 23 (November 2023): 7707. 10.3390/molecules28237707 PMC10708148 38067440

[220] C. E. McLaren , G. J. McLachlan , J. W. Halliday , et al., “Distribution of Transferrin Saturation in an Australian Population: Relevance to the Early Diagnosis of Hemochromatosis,” Gastroenterology 114, no. 3 (March 1998): 543–549. 10.1016/s0016-5085(98)70538-4 9496946

[221] M. Berkovitch , T. Bistritzer , S. D. Milone , et al., “Iron Deposition in the Anterior Pituitary in Homozygous Beta‐thalassemia: MRI Evaluation and Correlation With Gonadal Function,” Journal of Pediatric Endocrinology and Metabolism 13, no. 2 (February 2000): 179–184. 10.1515/jpem.2000.13.2.179 10711663

[222] S. I. K. Karadag , Z. Karakas , Y. Yilmaz , et al., “Pituitary Iron Deposition and Endocrine Complications in Patients With β‐Thalassemia: From Childhood to Adulthood,” Hemoglobin 44, no. 5 (September 2020): 344–348. 10.1080/03630269.2020.1812636 32900239

[223] L. J. Noetzli , S. D. Mittelman , R. M. Watanabe , T. D. Coates , and J. C. Wood , “Pancreatic Iron and Glucose Dysregulation in Thalassemia Major,” American Journal of Hematology 87, no. 2 (February 2012): 155–160. 10.1002/ajh.22223 22120775

[224] L. Anderson , “Cardiovascular T2‐Star (T2*) Magnetic Resonance for the Early Diagnosis of Myocardial Iron Overload,” European Heart Journal 22, no. 23 (December 2001): 2171–2179. 10.1053/euhj.2001.2822 11913479

[225] W.‐Y. Au , W. W.‐M. Lam , W. Chu , et al., “A T2* Magnetic Resonance Imaging Study of Pancreatic Iron Overload in Thalassemia Major,” Haematologica 93, no. 1 (January 2008): 116–119. 10.3324/haematol.11768 18166794

[226] M. Hashemieh , M. Radfar , A. Azarkeivan , G. Noghabaei , and K. Sheibani , “T2* magnetic Resonance Imaging Study of Pancreatic Iron Overload and Its Relation With the Diabetic State in Thalassemic Patients,” Journal of Pediatric Hematology/Oncology 39, no. 5 (July 2017): 337–340. 10.1097/MPH.0000000000000767 28085743

[227] M. Ouederni , M. Ben Khaled , F. Mellouli , et al., “Myocardial and Liver Iron Overload, Assessed Using T2* Magnetic Resonance Imaging With an Excel Spreadsheet for Post Processing in Tunisian Thalassemia Major Patients,” Annals of Hematology 96, no. 1 (January 2017): 133–139. 10.1007/s00277-016-2841-5 27730342

[228] D. Fintini , A. Grossi , C. Brufani , et al., “Bone Mineral Density and Body Composition in Male Children With Hypogonadism,” Journal of Endocrinological Investigation 32, no. 7 (July 2009): 585–589. 10.1007/BF03346513 19535890

[229] M. Tenuta , V. Hasenmajer , D. Gianfrilli , and A. M. Isidori , “Testosterone and Male Bone Health: A Puzzle of Interactions,” The Journal of Clinical Endocrinology & Metabolism 110, no. 7 (June 2025): e2121–e2135. 10.1210/clinem/dgaf191 40120082

[230] V. Jeney , “Clinical Impact and Cellular Mechanisms of Iron Overload‐Associated Bone Loss,” Frontiers in Pharmacology 8 (2017): 77. 10.3389/fphar.2017.00077 PMC5318432 28270766

[231] S. Gupta , S. Bhargava , R. Kumar , and S. Kakkar , “Effect of Iron Overload on Bone Mineral Density in Patients With Transfusion‐Dependent Thalassemia,” Journal of The Association of Physicians of India 73, no. 6 (June 2025): 40–44. 10.59556/japi.73.1029 40553589

[232] P. D. Phatak , D. H. Ryan , J. Cappuccio , et al., “Prevalence and Penetrance of Hfe Mutations in 4865 Unselected Primary Care Patients,” Blood Cells, Molecules, and Diseases 29, no. 1 (July‐August 2002): 41–47. 10.1006/bcmd.2002.0536 12482402

[233] C. Abadía Molina , N. Goñi Ros , R. González Tarancón , L. Rello Varas , M. D. V. Recasens Flores , and S. Izquierdo Álvarez , “Hereditary Haemochromatosis: Prevalence and Characterization of the Disease in a Tertiary Hospital in Aragon, Spain,” Medicina Clínica 163, no. 9 (November 2024): 442–448. 10.1016/j.medcli.2024.05.015 39003111

[234] S. P. Demirdjian , R. E. Irwin , P. D. Thompson , et al., “Maternal Adiposity and DNA Methylation of TFR2 and HJV Genes in Early Pregnancy: Mediating Role of Inflammation and Consequences for Iron Status,” The Journal of Nutrition 156, no. 4 (April 2026): 101393. 10.1016/j.tjnut.2026.101393 PMC13084681 41651072

[235] Z. Huang , S. Zhou , Z. Cui , et al., “Dha Ameliorates Gdm Placental Angiogenesis by Regulating Hamp‐Mediated Iron Imbalance,” Journal of Advanced Research (March 2026): S2090‐1232(26)00224‐9. 10.1016/j.jare.2026.03.005 41812706

[236] N. Bharadwaj , S. Peyam , P. Bhatia , et al., “Impact of HFE‐2 and Hamp Gene Variations on Iron Overload in Pediatric Patients With Non‐Transfusion Dependent Thalassemia: A Pilot Study,” Indian Journal of Hematology and Blood Transfusion 38, no. 1 (January 2022): 158–163. 10.1007/s12288-021-01442-9 PMC8804030 35125723

[237] L. Huang , L. Pan , W. Qin , Y. Zhao , Y. Huang , and X. Qin , “Slc40a1 (iron transporter): Mechanistic Regulation, Role in Disease Pathogenesis, and Prospects for Targeted Therapy,” Frontiers in Cell and Developmental Biology 14 (2026): 1816800. 10.3389/fcell.2026.1816800 PMC13199365 42199341

[238] Q. Dang , C. Huang , Y. Liang , et al., “Downregulation of SLC40A1 Leads to Iron Accumulation in Fibrotic Lung Fibroblasts,” Respiratory Research 27, no. 1 (January 2026): 91. 10.1186/s12931-025-03479-0 PMC12922364 41606738

[239] M. A. Lescano , L. C. Tavares , and P. Santos , “Juvenile Hemochromatosis: HAMP Mutation and Severe Iron Overload Treated With Phlebotomies and Deferasirox,” World Journal of Clinical Cases 5, no. 10 (October 2017): 381–383. 10.12998/wjcc.v5.i10.381 PMC5649000 29085829

[240] T. M. Peters , A. F. Meulders , K. Redert , et al., “TFR2‐Related Haemochromatosis in the Netherlands: A Cause of Arthralgia in Young Adulthood,” Netherlands Journal of Medicine 75, no. 2 (March 2017): 56–64. 28276324

[241] V. Gerolami , G. Le Gac , L. Mercier , M. Nezri , J. L. Berge‐Lefranc , and C. Ferec , “Early‐Onset Haemochromatosis Caused by a Novel Combination of TFR2 Mutations(p.R396x/c.1538‐2 a>g) in a Woman of Italian Descent,” Haematologica 93, no. 5 (2008): e45–46. 10.3324/haematol.12884 18450729

[242] C. Camaschella , A. Roetto , A. Calì , et al., “The Gene TFR2 Is Mutated in a New Type of Haemochromatosis Mapping to 7q22,” Nature Genetics 25, no. 1 (May 2000): 14–15. 10.1038/75534 10802645

[243] G. Ravasi , S. Pelucchi , A. Russo , R. Mariani , and A. Piperno , “Ferroportin Disease: A Novel SLC40A1 Mutation,” Digestive and Liver Disease 52, no. 6 (June 2020): 688–690. 10.1016/j.dld.2020.03.013 32360131

[244] A. Vadivelan , S. Zhang , D. N. Srole , et al., “HJV Mutations Causing Hemochromatosis: Variable Phenotypic Expression in a Pair of Twins,” Haematologica 109, no. 8 (August 2024): 2741–2744. 10.3324/haematol.2023.284134 PMC11290577 38450514

[245] J. C. Barton , J. C. Barton , and R. T. Acton , “Mean Corpuscular Volume in HFE p.C282Y/p.H63D Compound Heterozygotes with High Iron Phenotypes: Clinical and Laboratory Associations,” Journal of Hematology 15, no. 1 (February 2026): 13–22. 10.14740/jh2155 PMC12948473 41768868

[246] D. Li , J. Li , H. Zhang , et al., “Hereditary Hemochromatosis Caused by a C282Y/H63D Mutation in the HFE Gene: A Case Report,” Heliyon 10, no. 7 (April 2024): e28046. 10.1016/j.heliyon.2024.e28046 PMC10979142 38560130

[247] J. C. Barton , S. A. LaFreniere , C. Leiendecker‐Foster , et al., “HFE, SLC40A1, HAMP, HJV, TFR2 , and FTL Mutations Detected by Denaturing High‐Performance Liquid Chromatography After Iron Phenotyping and HFE C282Y and H63D Genotyping in 785 HEIRS Study Participants,” American Journal of Hematology 84, no. 11 (November 2009): 710–714. 10.1002/ajh.21524 19787796

[248] M. Batool , N. Ehsan , M. Imran , W. Qaisar , and M. N. Malik , “The Quiet Burden of Iron: A Rare Case of Hereditary Hemochromatosis in Pakistan,” Cureus 17, no. 7 (July 2025): e88355. 10.7759/cureus.88355 PMC12362553 40837903

[249] P. R. Galle , A. Forner , J. M. Llovet , et al., “EASL Clinical Practice Guidelines: Management of Hepatocellular Carcinoma,” Journal of hepatology 69, no. 1 (2018): 182–236. 10.1016/j.jhep.2018.03.019 29628281

[250] S. M. Wazir and I. Ghobrial , “Copper Deficiency, a New Triad: Anemia, Leucopenia, and Myeloneuropathy,” Journal of Community Hospital Internal Medicine Perspectives 7, no. 4 (October 2017): 265–268. 10.1080/20009666.2017.1351289 PMC5637704 29046759

[251] M. Xie , H. Yu , and B. Yao , “Bilateral Dense Kayser‐Fleischer Rings in Wilson's Disease,” Eye Lond (January 2026). 10.1038/s41433-025-04219-w 41501288

[252] J. F. Ortiz , A. Morillo Cox , and W. Tambo , “Neurological Manifestations of Wilson's Disease: Pathophysiology and Localization of Each Component,” Cureus 12, no. 11 (November 2020): e11509. 10.7759/cureus.11509 PMC7744205 33354453

[253] B. Tarnacka , A. Jopowicz , and M. Maślińska , “Copper, Iron, and Manganese Toxicity in Neuropsychiatric Conditions,” International Journal of Molecular Sciences 22, no. 15 (July 2021): 7820. 10.3390/ijms22157820 PMC8346158 34360586

[254] S. El Balkhi , J. M. Trocello , and J. Poupon , “Relative Exchangeable Copper: A New Highly Sensitive and Highly Specific Biomarker for Wilson's Disease Diagnosis,” Clinica Chimica Acta 412, no. 23‐24 (November 2011): 2254–2260. 10.1016/j.cca.2011.08.019 21878323

[255] D. Spirea , C. Vanlemmens , F. Parant , et al., “Performance of Relative Exchangeable Copper for the Diagnosis of Wilson Disease in Acute Liver Failure,” Journal of Inherited Metabolic Disease 48, no. 2 (March 2025): e70024. 10.1002/jimd.70024 PMC11913634 40097333

[256] M. Dezortova , A. Lescinskij , P. Dusek , et al., “Multiparametric Quantitative Brain MRI in Neurological and Hepatic Forms of Wilson's Disease,” Journal of Magnetic Resonance Imaging 51, no. 6 (June 2020): 1829–1835. 10.1002/jmri.26984 31710776

[257] T. Litwin , B. Rędzia‐Ogrodnik , A. Antos , A. Przybyłkowski , A. Członkowska , and J. P. ł. Bembenek , “Brain Magnetic Resonance Imaging in Wilson's Disease—Significance and Practical Aspects—A Narrative Review,” Brain Sciences 14, no. 7 (July 2024): 727. 10.3390/brainsci14070727 PMC11274939 39061467

[258] B. Rędzia‐Ogrodnik , A. Członkowska , A. Antos , et al., “Pathognomonic Neuroradiological Signs in Wilson's Disease—Truth or Myth?,” Parkinsonism & Related Disorders 107 (February 2023): 105247. 10.1016/j.parkreldis.2022.105247 36543734

[259] V. D and K. Ap , “Wilson's Disease in Childhood and the Challenges in Its Diagnosis: A Case Report,” Cureus 16, no. 7 (July 2024): e65847. 10.7759/cureus.65847 PMC11363881 39219963

[260] H. S. Aboalam , M. K. Hassan , N. El‐domiaty , et al., “Challenges and Recent Advances in Diagnosing Wilson Disease,” Journal of Clinical and Experimental Hepatology 15, no. 4 (July‐August 2025): 102531. 10.1016/j.jceh.2025.102531 PMC11952840 40160676

[261] European Association for the Study of the Liver , EASL‐ERN Clinical Practice Guidelines on Wilson's Disease, Journal of Hepatology 84, no. 4 (February 2025): 690–728. 10.1016/j.jhep.2024.11.007 40089450

[262] M. Li , J. Ma , W. Wang , X. Yang , and K. Luo , “Mutation Analysis of the ATP7B Gene and Genotype–Phenotype Correlation in Chinese Patients With Wilson Disease,” BMC Gastroenterology 21, no. 1 (September 2021): 339. 10.1186/s12876-021-01911-5 PMC8411542 34470610

[263] B. Gul , S. Firasat , R. Tehreem , T. Shan , and K. Afshan , “Analysis of Wilson Disease Mutations in Copper Binding Domain of ATP7B Gene,” PLoS ONE 17, no. 6 (2022): e0269833. 10.1371/journal.pone.0269833 PMC9239485 35763513

[264] A. Matsumoto , S. Kano , N. Kobayashi , et al., “Unfavorable Switching of Skewed X Chromosome Inactivation Leads to Menkes Disease in a Female Infant,” Scientific Reports 14, no. 1 (January 2024): 440. 10.1038/s41598-023-50668-2 PMC10764769 38172222

[265] A. Backal , M. Velinov , J. Garcia , and C. L. Louis , “Novel, Likely Pathogenic Variant in ATP7A Associated With Menkes Disease Diagnosed With Ultrarapid Genome Sequencing,” BMJ Case Reports 17, no. 10 (October 2024): e259792. 10.1136/bcr-2024-259792 39353672

[266] L. Venkataraman , C. J. Jeanty , T. Kaniganti , et al., “Intravenous AAV9‐ATP7A Plus Subcutaneous Copper Histidinate Optimizes Outcomes in a Lethal Menkes Disease Mouse Model,” Science Advances 11, no. 35 (August 2025): eadw5612. 10.1126/sciadv.adw5612 PMC12396322 40880469

[267] A. Papadopoulou , K. Nathavitharana , M. D. Williams , P. J. Darbyshire , and I. W. Booth , “Diagnosis and Clinical Associations of Zinc Depletion Following Bone Marrow Transplantation,” Archives of Disease in Childhood 74, no. 4 (April 1996): 328–331. 10.1136/adc.74.4.328 PMC1511474 8669934

[268] B. Çelik , H. Nalçacıoğlu , Ç. L. Karakükçü , H. Aslaner , and Ü. M. Şahiner , “Assessment of Hair Zinc in the School Children in Kayseri, Turkey,” Biological Trace Element Research 196, no. 2 (August 2020): 343–348. 10.1007/s12011-020-02115-w 32221800

[269] M. Ren , L. Yan , Y. Pang , et al., “External Interference From Ambient Air Pollution on Using Hair Metal(loid)s for Biomarker‐Based Exposure Assessment,” Environment International 137 (April 2020): 105584. 10.1016/j.envint.2020.105584 32106049

[270] D. Plantone , G. Primiano , R. Renna , et al., “Copper Deficiency Myelopathy: A Report of Two Cases,” The Journal of Spinal Cord Medicine 38, no. 4 (July 2015): 559–562. 10.1179/2045772314Y.0000000268 PMC4612215 25343982

[271] D. LeRoith , J. M. P. Holly , and B. E. Forbes , “Insulin‐Like Growth Factors: Ligands, Binding Proteins, and Receptors,” Molecular Metabolism 52 (2021): 101245, , 2021/10/01. 10.1016/j.molmet.2021.101245 PMC8513159 33962049

[272] C. Carter , D. Luz , E. E. Smith , et al., “Transient Neonatal Zinc Deficiency due to Maternal Variants in SLC30A2: An Emerging and Atypical Candidate Gene for Maternal Carrier Screening,” American Journal of Medical Genetics Part A 200, no. 8 (2026): 1868–1873. 10.1002/ajmg.a.70131 41866816

[273] D. B. D. Steel , F. R. Danti , M. Abunada , et al., “Clinical Phenotype in Individuals with Birk‐Landau‐Perez Syndrome Associated with Biallelic SLC30A9 Pathogenic Variants,” Neurology 100, no. 21 (May 2023): e2214–e2223. 10.1212/WNL.0000000000207241 PMC10238160 37041080

[274] Y. Perez , Z. Shorer , K. Liani‐Leibson , et al., “SLC30A9 Mutation Affecting Intracellular Zinc Homeostasis Causes a Novel Cerebro‐Renal Syndrome,” Brain 140, no. 4 (April 2017): 928–939. 10.1093/brain/awx013 PMC5837213 28334855

[275] S. A. Ahsan , M. Lackovic , A. Katner , and C. Palermo , “Metal Fume Fever: A Review of the Literature and Cases Reported to the Louisiana Poison Control Center,” Journal of the Louisiana State Medical Society 161, no. 6 (November‐December 2009): 348–351. 20108830

[276] M. I. Greenberg and D. Vearrier , “Metal Fume Fever and Polymer Fume Fever,” Clinical Toxicology 53, no. 4 (May 2015): 195–203. 10.3109/15563650.2015.1013548 25706449

[277] B. L. Clarke , “Epidemiology and Complications of Hypoparathyroidism,” Endocrinology and Metabolism Clinics of North America 47, no. 4 (December 2018): 771–782. 10.1016/j.ecl.2018.07.004 30390812

[278] B. Abu Raya , I. Koren , D. Bader , A. Kugelman , L. Borenstein‐Levin , and A. Riskin , “Transient Hypercalcemia in Preterm Infants: Insights Into Natural History and Laboratory Evaluation,” Glob Pediatr Health 1 (2014): 2333794×14560818. 10.1177/2333794X14560818 PMC4804696 27335919

[279] J. O. Jacobson , F. Richard Bringhurst , N. L. Harris , S. A. Weitzman , and A. C. Aisenberg , “Humoral Hypercalcemia in Hodgkin's Disease. Clinical and Laboratory Evaluation,” Cancer 63, no. 5 (March 1989): 917–923. 10.1002/1097-0142(19890301)63:5<917::aid-cncr2820630522>3.0.co;2-e 2536588

[280] G. Lum , “Evaluation of a Laboratory Critical Limit (Alert Value) Policy for Hypercalcemia,” Archives of Pathology & Laboratory Medicine 120, no. 7 (July 1996): 633–636. 8757466

[281] S. Hussein , S. Murad , O. Dawod , et al., “Biochemical Association Between Calcium Homeostasis and Serum Uric Acid Levels in Patients With Hypothyroidism: A Comparative Evaluation With 25‐Hydroxyvitamin D,” Georgian Med News 369 (December 2025): 174–179. 41687651

[282] N. Yedla , H. Kim , A. Sharma , and X. Wang , “Vitamin D Deficiency and the Presentation of Primary Hyperparathyroidism: A Mini Review,” International Journal of Endocrinology 2023 (2023): 1–8. 10.1155/2023/1169249 PMC10728357 38115826

[283] F. Cetani , F. Bertoldo , M. Bononi , et al., “Unveiling the Complexities of Hypoparathyroidism: A Comprehensive Review of Clinical Manifestations, Diagnosis, and Novel Therapies,” Journal of Endocrinological Investigation 49, no. 4 (April 2026): 725–746. 10.1007/s40618-025-02760-9 PMC13053346 41335198

[284] M. A. Levine , “Pseudohypoparathyroidism: From Bedside to Bench and Back,” Journal of Bone and Mineral Research 14, no. 8 (August 1999): 1255–1260. 10.1359/jbmr.1999.14.8.1255 10457257

[285] J. Martel‐Villagrán , A. Arias‐Medina , and G. García‐Mardones , “Usefulness of X‐Rays in the Differential Diagnosis of Hypophosphataemic Rickets,” Advances in Therapy 37, no. Suppl 2 (May 2020): 89–94. 10.1007/s12325-019-01183-2 32236877

[286] R. Thillaigovindan , E. Arumugam , R. Rai , P. R , and R. Kesavan , “Idiopathic Basal Ganglia Calcification: Fahr's Syndrome, a Rare Disorder,” Cureus 11, no. 10 (October 2019): e5895. 10.7759/cureus.5895 PMC6839755 31772865

[287] Y. Shen , W. Yang , Q. He , et al., “Clinical and Genetic Characteristics of Hypoparathyroidism in Children: A Multicenter Experience in China,” Journal of Endocrinological Investigation 48, no. 2 (February 2025): 393–400. 10.1007/s40618-024-02465-5 39531207

[288] A. Pietrangelo , “Hereditary Hemochromatosis: Pathogenesis, Diagnosis, and Treatment,” Gastroenterology 139, no. 2 (August 2010): 393–408.e2, , 408 e391‐392. 10.1053/j.gastro.2010.06.013 20542038

[289] P. J. Tebben , R. J. Singh , and R. Kumar , “Vitamin D‐mediated Hypercalcemia: Mechanisms, Diagnosis, and Treatment,” Endocrine Reviews 37, no. 5 (October 2016): 521–547. 10.1210/er.2016-1070 PMC5045493 27588937

[290] M. F. Carroll and D. S. Schade , “A Practical Approach to Hypercalcemia,” American Family Physician 67, no. 9 (May 2003): 1959–1966. 12751658

[291] A. M. Patel and S. Goldfarb , “Got Calcium? Welcome to the Calcium‐Alkali Syndrome,” Journal of the American Society of Nephrology 21, no. 9 (September 2010): 1440–1443. 10.1681/ASN.2010030255 20413609

[292] M. D. Walker and E. Shane , “Hypercalcemia,” Jama 328, no. 16 (October 2022): 1624. 10.1001/jama.2022.18331 36282253

[293] S. Minisola , J. Pepe , S. Piemonte , and C. Cipriani , “The Diagnosis and Management of Hypercalcaemia,” Bmj 350 (June 2015): h2723. 10.1136/bmj.h2723 26037642

[294] A. D. Anastasilakis , M. P. Yavropoulou , E. Kassi , et al., “Diagnosis and Management of Primary Hyperparathyroidism: Recommendations From the Bone Section of the hellenic Endocrine Society,” Hormones 24, no. 4 (December 2025): 905–912. 10.1007/s42000-025-00681-z 40459662

[295] C. C. Nava Suarez , C. Anastasopoulou , and P. Kathuria , “Familial Hypocalciuric Hypercalcemia,” in Statpearls (StatPearls Publishing, 2026). 29083672

[296] S. Lipten , “Cancer‐Associated Hypercalcemia,” New England Journal of Medicine 386, no. 26 (June 2022): 2540. 10.1056/NEJMc2206287 35767455

[297] T. A. Guise and J. J. Wysolmerski , “Cancer‐Associated Hypercalcemia,” New England Journal of Medicine 386, no. 15 (April 2022): 1443–1451. 10.1056/NEJMcp2113128 35417639

[298] O. P. Sharma , “Hypercalcemia in Granulomatous Disorders: A Clinical Review,” Current Opinion in Pulmonary Medicine 6, no. 5 (September 2000): 442–447. 10.1097/00063198-200009000-00010 10958237

[299] M. L. Kometas and N. M. Maalouf , “Hypercalcemia of Malignancy: Mechanisms and Therapeutic Implications of Concurrently Elevated PTHRP and Calcitriol,” Journal of the Endocrine Society 9, no. 9 (September 2025): bvaf104. 10.1210/jendso/bvaf104 PMC12257092 40662114

[300] E. Castellano , R. Attanasio , A. Boriano , and G. Borretta , “Clinical Presentation of Primary Hyperparathyroidism in Older Adults,” Journal of the Endocrine Society no. 12 (December 2019): 2305–2312. 10.1210/js.2019-00316 PMC6853663 31745527

[301] R. Schreckenberg and K. D. Schluter , “Calcium Sensing Receptor Expression and Signalling in Cardiovascular Physiology and Disease,” Vascul Pharmacol (March 2018): 35–42. 10.1016/j.vph.2018.02.007 29514057

[302] D. Riccardi and P. J. Kemp , “The Calcium‐sensing Receptor Beyond Extracellular Calcium Homeostasis: Conception, Development, Adult Physiology, and Disease,” Annual Review of Physiology 74 (2012): 271–297. 10.1146/annurev-physiol-020911-153318 22017175

[303] G. N. Hendy , V. Guarnieri , and L. Canaff , “Calcium‐Sensing Receptor and Associated Diseases,” Prog Mol Biol Transl Sci 89 (2009): 31–95. 10.1016/S1877-1173(09)89003-0 20374733

[304] S. A. Wells, Jr. , F. Pacini , B. G. Robinson , and M. Santoro , “Multiple Endocrine Neoplasia Type 2 and Familial Medullary Thyroid Carcinoma: An Update,” The Journal of Clinical Endocrinology & Metabolism 98, no. 8 (August 2013): 3149–3164. 10.1210/jc.2013-1204 PMC5399478 23744408

[305] W. Zhang , M. Doherty , E. Pascual , et al., “Eular Recommendations for Calcium Pyrophosphate Deposition. Part II: Management,” Annals of the Rheumatic Diseases 70, no. 4 (April 2011): 571–575. 10.1136/ard.2010.139360 21257614

[306] T. S. H. Kwok and G. Choy , “Calcium Pyrophosphate Deposition Disease,” Canadian Medical Association Journal 198, no. 4 (February 2026): E130. 10.1503/cmaj.250933 PMC12880869 41628946

[307] S. Cowley and G. McCarthy , Open Access Rheumatology: Research and Reviews 15 (2023): 33–41. 10.2147/OARRR.S389664 PMC10040153 36987530

[308] J. L. Rosales‐Alexander , J. Balsalobre Aznar , and C. Magro‐Checa , “Calcium Pyrophosphate Crystal Deposition Disease: Diagnosis and Treatment,” Open Access Rheumatology: Research and Reviews 6 (2014): 39. 10.2147/OARRR.S39039 PMC5045115 27790033

[309] D. Resnick , G. Niwayama , T. G. Goergen , et al., “Clinical, Radiographic and Pathologic Abnormalities in Calcium Pyrophosphate Dihydrate Deposition Disease (cppd): Pseudogout,” Radiology 122, no. 1 (January 1977): 1–15. 10.1148/122.1.1 186841

[310] J. Miksanek and A. K. Rosenthal , “Imaging of Calcium Pyrophosphate Deposition Disease,” Current Rheumatology Reports 17, no. 3 (March 2015): 20. 10.1007/s11926-015-0496-1 PMC5471493 25761927

[311] F. Becce , “Diagnosis of Calcium Pyrophosphate Deposition by Imaging—current state and Challenges Remaining,” Osteoarthritis and Cartilage 27, no. 4 (April 2019): 545–546. 10.1016/j.joca.2019.01.015 30743016

[312] T. Pascart , G. Falgayrac , L. Norberciak , et al., “Dual‐Energy Computed‐tomography‐Based Discrimination Between Basic Calcium Phosphate and Calcium Pyrophosphate Crystal Deposition in Vivo,” Therapeutic Advances in Musculoskeletal Disease 12 (2020): 1759720×20936060. 10.1177/1759720X20936060 PMC7315653 32636945

[313] M. Forien , A. Combier , A. Gardette , E. Palazzo , P. Dieudé , and S. Ottaviani , “Comparison of Ultrasonography and Radiography of the Wrist for Diagnosis of Calcium Pyrophosphate Deposition,” Joint, Bone, Spine 85, no. 5 (October 2018): 615–618. 10.1016/j.jbspin.2017.09.006 28965942

[314] W. Grassi , T. Okano , and E. Filippucci , “Use of Ultrasound for Diagnosis and Monitoring of Outcomes in Crystal Arthropathies,” Current Opinion in Rheumatology 27, no. 2 (March 2015): 147–155. 10.1097/BOR.0000000000000142 25633243

[315] E. Cipolletta , E. Filippucci , A. Abhishek , et al., “In Patients With Acute Mono/Oligoarthritis, a Targeted Ultrasound Scanning Protocol Shows Great Accuracy for the Diagnosis of Gout and CPPD,” Rheumatology 62, no. 4 (April 2023): 1493–1500. 10.1093/rheumatology/keac479 35997554

[316] G. Filippou , C. A. Scirè , N. Damjanov , et al., “Definition and Reliability Assessment of Elementary Ultrasonographic Findings in Calcium Pyrophosphate Deposition Disease: A Study by the Omeract Calcium Pyrophosphate Deposition Disease Ultrasound Subtask Force,” The Journal of Rheumatology 44, no. 11 (November 2017): 1744–1749. 10.3899/jrheum.161057 28250136

[317] M. Gutierrez , L. Di Geso , F. Salaffi , et al., “Ultrasound Detection of Cartilage Calcification at Knee Level in Calcium Pyrophosphate Deposition Disease,” Arthritis Care & Research 66, no. 1 (January 2014): 69–73. 10.1002/acr.22190 24115756

[318] G. Filippou , A. Scanu , A. Adinolfi , et al., “Criterion Validity of Ultrasound in the Identification of Calcium Pyrophosphate Crystal Deposits at the Knee: An Omeract Ultrasound Study,” Annals of the Rheumatic Diseases 80, no. 2 (February 2021): 261–267. 10.1136/annrheumdis-2020-217998 32988839

[319] J. C. Suan , R. K. Chhem , J. S. Gati , C. J. Norley , and D. W. Holdsworth , “4 T MRI of Chondrocalcinosis in Combination With Three‐dimensional CT, Radiography, and Arthroscopy: A Report of Three Cases,” Skeletal Radiology 34, no. 11 (November 2005): 714–721. 10.1007/s00256-005-0930-y 16167133

[320] C. J. Williams , Y. Zhang , A. Timms , et al., “Autosomal Dominant Familial Calcium Pyrophosphate Dihydrate Deposition Disease Is Caused by Mutation in the Transmembrane Protein Ankh,” The American Journal of Human Genetics 71, no. 4 (October 2002): 985–991. 10.1086/343053 PMC419998 12297989

[321] A. Pendleton , M. D. Johnson , A. Hughes , et al., “Mutations in Ankh Cause Chondrocalcinosis,” The American Journal of Human Genetics 71, no. 4 (October 2002): 933–940. 10.1086/343054 PMC378546 12297987

[322] C. Borgna‐Pignatti , S. Rugolotto , P. De Stefano , et al., “Survival and Complications in Patients With Thalassemia Major Treated With Transfusion and Deferoxamine,” Haematologica 89, no. 10 (October 2004): 1187–1193. 15477202

[323] M. G. Vogiatzi , E. A. Macklin , F. L. Trachtenberg , et al., “Differences in the Prevalence of Growth, Endocrine and Vitamin D Abnormalities Among the Various Thalassaemia Syndromes in North America,” British Journal of Haematology 146, no. 5 (September 2009): 546–556. 10.1111/j.1365-2141.2009.07793.x PMC2798591 19604241

[324] G. Derchi , F. Formisano , M. Balocco , et al., “Clinical Management of Cardiovascular Complications in Patients With Thalassaemia Major: A Large Observational Multicenter Study,” European Journal of Echocardiography 12, no. 3 (March 2011): 242–246. 10.1093/ejechocard/jeq190 21278193

[325] M. Bordbar , H. Bozorgi , F. Saki , et al., “Prevalence of Endocrine Disorders and Their Associated Factors in Transfusion‐Dependent Thalassemia Patients: A Historical Cohort Study in Southern Iran,” Journal of Endocrinological Investigation 42, no. 12 (December 2019): 1467–1476. 10.1007/s40618-019-01072-z 31228105

[326] V. Berdoukas , K. Farmaki , S. Carson , J. Wood , and T. Coates , “Treating Thalassemia Major‐Related Iron Overload: The Role of Deferiprone,” Journal of Blood Medicine 3 (2012): 119. 10.2147/JBM.S27400 PMC3480237 23112580

[327] I. Goldstein , N. Chidambaram , A. Dobs , et al., “Newer Formulations of Oral Testosterone Undecanoate: Development and Liver Side Effects,” Sexual Medicine Reviews 13, no. 1 (January 2025): 33–40. 10.1093/sxmrev/qeae062 39291780

[328] J. Karlberg , L. A. Mattsson , and I. Wiklund , “A Quality of Life Perspective on Who Benefits From Estradiol Replacement Therapy,” Acta Obstetricia et Gynecologica Scandinavica 74, no. 5 (May 1995): 367–372. 10.3109/00016349509024430 7778430

[329] A. D. Divasta and C. M. Gordon , “Hormone Replacement Therapy and the Adolescent,” Current Opinion in Obstetrics & Gynecology 22, no. 5 (October 2010): 363–368. 10.1097/GCO.0b013e32833e4a35 20724926

[330] J. P. Friedmann Angeli , M. Schneider , B. Proneth , et al., “Inactivation of the Ferroptosis Regulator gpx4 Triggers Acute Renal Failure in Mice,” Nature Cell Biology 16, no. 12 (December 2014): 1180–1191. 10.1038/ncb3064 PMC4894846 25402683

[331] J. Zhang , T. Su , Y. Fan , C. Cheng , L. Xu , and LiTian , “Spotlight on Iron Overload and Ferroptosis: Research Progress in Female Infertility,” Life Sciences 340 (March 2024): 122370. 10.1016/j.lfs.2023.122370 38141854

[332] E. G. Bromfield , J. L. H. Walters , S. L. Cafe , et al., “Differential Cell Death Decisions in the Testis: Evidence for an Exclusive Window of Ferroptosis in Round Spermatids,” MHR: Basic science of reproductive medicine 25, no. 5 (May 2019): 241–256. 10.1093/molehr/gaz015 30865280

[333] R. M. Adel , R. A. Lotfy , A. S. Darwish , and A. S. Amer , “Destructive Effect of Iron Overload in Brain Tissue of Albino Rats: Ameliorative Role of Silver Immobilized Organo‐Modified Casein Nanocomposite as co‐treating Agent With Deferasirox,” Journal of Trace Elements in Medicine and Biology 67 (September 2021): 126794. 10.1016/j.jtemb.2021.126794 34052583

[334] M. Onrubia‐Márquez , F. Navas , E. M. Sánchez‐Carnerero , et al., “Size‐Controlled Mesoporous Silica Nanoparticles via Template Nanoarchitectonics From a Deferoxamine Derivative for Enhanced Blood–Brain Barrier Permeability and Neuroprotective Chelation Therapy,” ACS Applied Materials & Interfaces 17, no. 51 (December 2025): 69090–69106. 10.1021/acsami.5c18528 PMC12754760 41376474

[335] F. Zhao , M. Fan , Z. Jing , et al., “Engineered Nanoparticles Potentials in Male Reproduction,” Andrology 13, no. 4 (May 2025): 694–705. 10.1111/andr.13729 PMC12006894 39120563

[336] Y. Li , X. Ruan , M. Sun , et al., “Iron Deposition Participates in LPS‐induced Cognitive Impairment by Promoting Neuroinflammation and Ferroptosis in Mice,” Experimental Neurology 379 (September 2024): 114862. 10.1016/j.expneurol.2024.114862 38866103

[337] B. Du , Z. Deng , K. Chen , et al., “Iron Promotes Both Ferroptosis and Necroptosis in the Early Stage of Reperfusion in Ischemic Stroke,” Genes & Diseases 11, no. 6 (2024): 101262. 10.1016/j.gendis.2024.101262 PMC11402992 39286656

[338] W. Jian , H. Ma , Y. Hu , et al., “Oltipraz Attenuated Cerebral Ischemia–Reperfusion Injury Through Inhibiting the Oxidative Stress and Ferroptosis in Mice,” International Immunopharmacology 140 (2024): 112800, , 2024/10/25/. 10.1016/j.intimp.2024.112800 39096875

[339] M. Ishikawa , A. Uchiyama , K. Kosaka , et al., “Exposure to Volatile Ferroptosis Inhibitor, Tempo, Reduced Cutaneous Ischemia‐Reperfusion Injury Progression to Pressure Ulcer Formation in a Mouse Model,” Journal of Dermatological Science 115, no. 3 (September 2024): 130–140. 10.1016/j.jdermsci.2024.07.005 39098373

[340] Y. Ma , X. Yao , Y. Zou , et al., “Ticlopidine Protects Hepatic Ischemia‐Reperfusion Injury via Suppressing Ferroptosis,” Biochemical and Biophysical Research Communications 733 (November 2024): 150436. 10.1016/j.bbrc.2024.150436 39053102

[341] H. Zhang , H. Shi , X. Li , et al., “Quercetin Alleviates LPS/IE‐DAP‐Induced Liver Injury by Suppressing Ferroptosis via Regulating Ferritinophagy and Intracellular Iron Efflux,” Redox Biology 81 (April 2025): 103557. 10.1016/j.redox.2025.103557 PMC11904602 39986118

[342] S. Yao , X. Zhou , T. Liao , et al., “Ferrostatin‐1 Alleviates Experimental Cerebral Malaria by Regulating Immune Cell Functions and Brain Endothelial Ferroptosis,” International Journal for Parasitology: Drugs and Drug Resistance 30 (April 2026): 100630. 10.1016/j.ijpddr.2025.100630 PMC12795685 41422632

[343] X. Wang , T. Wei , J. Luo , et al., “Iron Overload–Dependent Ferroptosis Aggravates LPS‐Induced Acute Lung Injury by Impairing Mitochondrial Function,” Inflammation 47, no. 6 (December 2024): 2013–2026. 10.1007/s10753-024-02022-5 38722504

[344] M. Mo , L. Pan , L. Deng , M. Liang , N. Xia , and Y. Liang , “Iron Overload Induces Hepatic Ferroptosis and Insulin Resistance by Inhibiting THE JAK2/STAT3/SLC7A11 Signaling Pathway,” Cell Biochemistry and Biophysics 82, no. 3 (September 2024): 2079–2094. 10.1007/s12013-024-01315-8 38801513

[345] X. He , S. Wei , Y. Fu , H. Li , J. Zhang , and L. Liu , “Nuclear Protein 1 Protects Against Neonatal Hypoxic‐Ischemic Encephalopathy by Inhibiting Neuronal Ferroptosis by Improving Iron Storage,” In Vitro Cellular & Developmental Biology—Animal 61, no. 8 (September 2025): 977–990. 10.1007/s11626-025-01088-1 40745515

[346] T. Matsumoto , A. Nita , Y. Kanamori , et al., “Integrated Hepatic Ferroptosis Gene Signature Dictates Pathogenic Features of Ferroptosis,” Hepatology Communications 9, no. 6 (June 2025): e0721. 10.1097/HC9.0000000000000721 PMC12122173 40434703

[347] A. Stancic , M. Markelic , N. Savic , et al., “Early Pathological Changes in the Liver and Kidney of Non‐Obese Diabetic (NOD) Mice: Involvement of Iron Accumulation and Ferroptosis,” Frontiers in Endocrinology 16 (2025): 1673012. 10.3389/fendo.2025.1673012 PMC12527868 41113713

[348] L. Zhang , I.‐C. Tsai , Z. Ni , et al., “Copper Chelation Therapy Attenuates Periodontitis Inflammation Through the Cuproptosis/Autophagy/Lysosome Axis,” International Journal of Molecular Sciences 25, no. 11 (May 2024): 5890. 10.3390/ijms25115890 PMC11172687 38892077

[349] Z. Guo , J. Liu , G. Liang , et al., “Identification and Validation of Cuproptosis‐Related Genes in Acetaminophen‐Induced Liver Injury Using Bioinformatics Analysis and Machine Learning,” Frontiers in Immunology 15 (2024): 1371446. 10.3389/fimmu.2024.1371446 PMC11236684 38994365

[350] N. Chen , L. Guo , L. Wang , S. Dai , X. Zhu , and E. Wang , “Sleep Fragmentation Exacerbates Myocardial Ischemia‒Reperfusion Injury by Promoting Copper Overload in Cardiomyocytes,” Nature Communications 15, no. 1 (May 2024): 3834. 10.1038/s41467-024-48227-y PMC11076509 38714741

[351] Y. Chen , Y. Nan , L. Xu , et al., “Polystyrene Nanoplastics Exposure Induces Cognitive Impairment in Mice via Induction of Oxidative Stress and ERK/MAPK‐Mediated Neuronal Cuproptosis,” Particle and Fibre Toxicology 22, no. 1 (May 2025): 13. 10.1186/s12989-025-00633-w PMC12090536 40394693

[352] L. U. Wang , L. Jiang , J. Wang , et al., “Reduced Glutathione Ameliorates Sepsis‐Induced Acute Kidney Injury in Mouse Model: Involvement of Cuproptosis,” Renal Failure 47, no. 1 (December 2025): 2518227. 10.1080/0886022X.2025.2518227 PMC12893487 40545999

[353] T. Bai , L. Wang , Z. Qiao , and Z. Wang , “Cuproptosis, a Potential Target for the Therapy of Diabetic Critical Limb Ischemia,” Free Radical Biology and Medicine 234 (July 2025): 131–140. 10.1016/j.freeradbiomed.2025.04.022 40246253

[354] T. Wu , Y. Chen , Y. Cong , et al., “Qidantang Granule Ameliorates Diabetic Kidney Disease in Mice by Inhibiting Cuproptosis,” International Journal of Clinical Practice 2025, no. 1 (2025): 6675290.

[355] K. Yu , Y. Gu , Y. Yao , et al., Journal of Inflammation Research 18 (2025): 4651–4664. 10.2147/JIR.S512187 PMC11975008 40195958

[356] S. Fu , H. Du , X. Ling , et al., “Suppressing Chondrocyte Cuproptosis by Syringaresinol‐4‐O‐β‐D‐Glucoside Alleviates Gouty Arthritis,” Frontiers in Pharmacology 16 (2025): 1565422. 10.3389/fphar.2025.1565422 PMC12099060 40417211

[357] J. Dong , G. Xiang , X. Xia , et al., “Aberrant Copper Metabolism and Hepatic Inflammation Cause Neurological Manifestations in a Mouse Model of Wilson's Disease,” Journal of Neuroinflammation 21, no. 1 (September 2024): 235. 10.1186/s12974-024-03178-5 PMC11437830 39334421

[358] T.‐Y. Wenegieme , D. Elased , K. E. McMichael , et al., “Strategies for Inducing and Validating Zinc Deficiency and Zinc Repletion,” American Journal of Physiology‐Heart and Circulatory Physiology 326, no. 6 (June 2024): H1396–H1401. 10.1152/ajpheart.00025.2024 PMC11380977 38578238

[359] R. Luan , M. Luo , D. Ding , X. Su , and J. Yang , “Zinc Deficiency Increases Lung Inflammation and Fibrosis in Obese Mice by Promoting Oxidative Stress,” Biochimica et Biophysica Acta (BBA)—General Subjects 1868, no. 1 (January 2024): 130518. 10.1016/j.bbagen.2023.130518 37951369

[360] M. E. S. Sampah , H. Moore , R. Ahmad , et al., “Xenotransplanted Human Organoids Identify Transepithelial Zinc Transport as a Key Mediator of Intestinal Adaptation,” Nature Communications 15, no. 1 (October 2024): 8613. 10.1038/s41467-024-52216-6 PMC11458589 39375337

[361] K. Lee , Y. Jung , Y. Vyas , Z. Mills , L. McNamara , and J. M. Montgomery , “Differential Effectiveness of Dietary Zinc Supplementation With Autism‐Related Behaviours in SHANK2 Knockout Mice,” Philosophical Transactions B 379, no. 1906 (July 2024): 20230230. 10.1098/rstb.2023.0230 PMC11343228 38853567

[362] R. Zong , X. Zhang , X. Dong , et al., “Genetic Deletion of Zinc Transporter ZnT3 Induces Progressive Cognitive Deficits in Mice by Impairing Dendritic Spine Plasticity and Glucose Metabolism,” Frontiers in Molecular Neuroscience 17 (2024): 1375925. 10.3389/fnmol.2024.1375925 PMC11130425 38807922

[363] J. Rivers‐Auty , V. S. Tapia , C. S. White , et al., “Zinc Status Alters Alzheimer's Disease Progression Through NLRP3‐Dependent Inflammation,” The Journal of Neuroscience 41, no. 13 (March 2021): 3025–3038. 10.1523/JNEUROSCI.1980-20.2020 PMC8018890 33597269

[364] C. Qu , C. Liang , T. Liu , Y. He , K. Ke , and J. Miao , “Natural Zinc‐Rich Oyster Peptides Ameliorate DSS‐induced Colitis via Antioxidation, Anti‐Inflammation, Intestinal Barrier Repair, Microbiota Modulation, and SCFA Accumulation,” Food Bioscience 61 (2024): 104801, , 10/01/2024.

[365] C. J. Dube , M. Lai , Y. Zhang , et al., “T‐Type Calcium Channels Regulate Medulloblastoma and Can be Targeted for Therapy,” Journal of Neuro‐Oncology 173, no. 1 (2025): 121–130, , 2025/05/01. 10.1007/s11060-025-04967-5 PMC12041153 39961940

[366] E. A. Arafa , M. M. Abdel‐Fattah , E. H. M. Hassanein , M. A. Buabeid , and W. R. Mohamed , “Involvement of GSK‐3β, NF‐κB, PPARγ, and Apoptosis in Amlodipine's Anticancer Effect in BALB/c Mice,” Toxicology and Applied Pharmacology 498 (May 2025): 117298. 10.1016/j.taap.2025.117298 40089189

[367] W. Liu , R. Xie , Z. Chen , and Z. Wu , “Calcium Channel Blocker Amlodipine Suppresses Bladder Cancer by Regulating NFAT2 and Enhances Anti‐PD1 Immune Checkpoint Inhibitor Therapy,” Iranian Red Crescent Medical Journal (IRCMJ) 27, no. 1 (2025): e227149.

[368] J. Zhou , N. Wang , Y. U.‐K. Lin , et al., “Targeting nfat2 for Reversing the P‐GP‐mediated Multidrug Resistance to paclitaxel by Manidipine,” Cancers 17, no. 20 (October 2025): 3289. 10.3390/cancers17203289 PMC12562668 41154347

[369] P. Sharma , B. Sharma , S. Ghildiyal , and H. Kharkwal , “Ml218 Modulates Calcium Binding Protein, Oxidative Stress, and Inflammation During Ischemia‐Reperfusion Brain Injury in Mice,” European Journal of Pharmacology 982 (November 2024): 176919. 10.1016/j.ejphar.2024.176919 39179092

[370] Y.‐M. Chen , W.‐Q. Yang , C.‐W. Gu , Y.‐Y. Fan , Y.‐Z. Liu , and B.‐S. Zhao , “Amlodipine Inhibits the Proliferation and Migration of Esophageal Carcinoma Cells Through the Induction of Endoplasmic Reticulum Stress,” World Journal of Gastroenterology 30, no. 4 (2024): 367–380, , 2024/01/28. 10.3748/wjg.v30.i4.367 PMC10835542 38313237

[371] A. S. Chadha , A. U. L. H. K. Mohammad , N. K. D. Nadakuditi , et al., “Dual Oral Iron Chelation (DOIC) in Children With Transfusion‐Dependent Beta Thalassemia: Real‐World Efficacy Data,” Indian Journal of Hematology and Blood Transfusion 41, no. 1 (January 2025): 167–170. 10.1007/s12288-024-01804-z PMC11794929 39917499

[372] A. Premawardhena , S. Wanasinghe , C. Perera , et al., “Deferoxamine, Deferasirox, and Deferiprone Triple Iron Chelator Combination Therapy for Transfusion‐Dependent β‐Thalassaemia With Very High Iron Overload: A Randomised Clinical Trial,” The Lancet Regional Health—Southeast Asia 30 (November 2024): 100495. 10.1016/j.lansea.2024.100495 PMC11718414 39802421

[373] M. Talha , M. H. Ali , S. Hurjkaliani , et al., “Beyond Blood Transfusions: Exploring Iron Chelation Therapies in Transfusion‐Dependent Beta‐Thalassemia,” Annals of Medicine & Surgery 87, no. 1 (January 2025): 13–17. 10.1097/MS9.0000000000002796 PMC11918563 40109613

[374] A. Lal , J. Porter , N. Sweeters , et al., “Combined Chelation Therapy With Deferasirox and Deferoxamine in Thalassemia,” Blood Cells, Molecules, and Diseases 50, no. 2 (February 2013): 99–104. 10.1016/j.bcmd.2012.10.006 PMC3592978 23151373

[375] A. Maggio , A. Vitrano , M. Capra , et al., “Long‐Term Sequential Deferiprone–Deferoxamine Versus Deferiprone Alone for Thalassaemia Major Patients: A Randomized Clinical Trial,” British Journal of Haematology 145, no. 2 (April 2009): 245–254. 10.1111/j.1365-2141.2009.07609.x 19236376

[376] D. J. Pennell , J. B. Porter , A. Piga , et al., “Sustained Improvements in Myocardial T2* Over 2 Years in Severely Iron‐Overloaded Patients With Beta Thalassemia Major Treated With Deferasirox or Deferoxamine,” American Journal of Hematology 90, no. 2 (February 2015): 91–96. 10.1002/ajh.23876 25345697

[377] M. L. Schilsky , A. Czlonkowska , M. Zuin , et al., “Trientine Tetrahydrochloride versus Penicillamine for Maintenance Therapy in Wilson Disease (chelate): A Randomised, Open‐Label, Non‐Inferiority, Phase 3 Trial,” The Lancet Gastroenterology & Hepatology 7, no. 12 (December 2022): 1092–1102. 10.1016/S2468-1253(22)00270-9 36183738

[378] K. H. Weiss , F. K. Askari , A. Czlonkowska , et al., “Bis‐Choline Tetrathiomolybdate in Patients With Wilson's Disease: An Open‐Label, Multicentre, Phase 2 Study,” The Lancet Gastroenterology & Hepatology 2, no. 12 (December 2017): 869–876. 10.1016/S2468-1253(17)30293-5 28988934

[379] X. Zhou , X. Xiao , X.‐H. Li , et al., “A Study of Susceptibility‐Weighted Imaging in Patients With Wilson Disease During the Treatment of Metal Chelator,” Journal of Neurology 267, no. 6 (June 2020): 1643–1650. 10.1007/s00415-020-09746-y 32060651

[380] R. Paniagua , R. Claure , D. Amato , E. Flores , A. Perez , and E. Exaire , “Effects of Oral Administration of Zinc and Diiodohydroxyquinolein on Plasma Zinc Levels of Uremic Patients,” Nephron 69, no. 2 (1995): 147–150. 10.1159/000188430 7723896

[381] S. Badulici and M. Chirila , “Zinc Supplementation of Chelation Therapy in Saturnism. Preliminary Study,” Romanian Journal of Internal Medicine 30, no. 3 (July‐September 1992): 211–215. 1475600

[382] S. J. Tingle , G. R. Severs , J. A. G. Moir , and S. A. White , “Calcium Channel Blockers in Pancreatic Cancer: Increased Overall Survival in a Retrospective Cohort Study,” Anti‐Cancer Drugs 31, no. 7 (August 2020): 737–741. 10.1097/CAD.0000000000000947 32639282

[383] S. Stasek , S. Reincke , M. Rehberg , O. Semler , and H. Hoyer‐Kuhn , “Urinary Calcium and Bone Resorption Markers During 3 Years of Denosumab Treatment in Pediatric Osteogenesis Imperfecta,” JBMR Plus 9, no. 10 (October 2025): ziaf140. 10.1093/jbmrpl/ziaf140 PMC12448440 40978125

[384] M. Holdhoff , X. Ye , J. G. Supko , et al., “Timed Sequential Therapy of the Selective T‐Type Calcium Channel Blocker Mibefradil and Temozolomide in Patients With Recurrent High‐Grade Gliomas,” Neuro‐Oncology 19, no. 6 (2017): 845–852. 10.1093/neuonc/nox020 PMC5464434 28371832

[385] H. W. Ejaz , W. Wang , and M. Lang , “Copper Toxicity Links to Pathogenesis of Alzheimer's Disease and Therapeutics Approaches,” International Journal of Molecular Sciences 21, no. 20 (October 16 2020): 7660. 10.3390/ijms21207660 PMC7589751 33081348

[386] W. Stremmel and R. Weiskirchen , “Therapeutic Strategies in Wilson Disease: Pathophysiology and Mode of Action,” Annals of Translational Medicine 9, no. 8 (April 2021): 732. 10.21037/atm-20-3090 PMC8106045 33987430

[387] W. Fang , Q. Wang , H. Yang , et al., “D ‐Penicillamine‐Loaded MIL‐100(Fe) for Precise Targeted Copper Chelation in Wilson's Disease,” RSC Advances 16, no. 5 (January 2026): 4373–4382. 10.1039/d5ra07832f PMC12817824 41568204

[388] A. Poujois , M. A. Obadia , N. Oussedik‐Djebrani , E. Couchonnal‐Bedoya , F. Ory‐Magne , and D. Debray , “Transition From Zinc Salts to Trientine Tetrahydrochloride in a Cohort of Adult Patients With Wilson Disease: The Zicup Study,” Orphanet Journal of Rare Diseases 21, no. 1 (April 2026): 200. 10.1186/s13023-026-04311-8 PMC13173772 41933366

[389] I. T. Khalil , M. Elshal , N. M. Elsayed , and A. Radwan , “Trientine Ameliorates Bleomycin‐Induced Pulmonary Fibrosis in Rats Through Copper Chelation and Modulation of the CTR1/LOX/COL Pathway,” Inflammopharmacology 34, no. 4 (April 2026): 2759–2775. 10.1007/s10787-026-02135-3 PMC13083351 41784930

[390] M. B. Delsouc , R. A. Conforti , A. S. Zabala , et al., “Copper‐Targeted Therapy in Experimental Endometriosis: Effects of Ammonium Tetrathiomolybdate on Markers of the Interconnected Processes of Inflammation, Innervation, and Fibrogenesis,” International Journal of Molecular Sciences 27, no. 2 (January 2026): 1099. 10.3390/ijms27021099 PMC12842519 41596737

[391] Y. Zhu , W. Peng , G. Liu , et al., “Tdmq20 as a Drug Candidate for Wilson's Disease: Comparison With D‐Penicillamine, Trientine, and Tetrathiomolybdate in Vitro and in Mice,” Pharmaceutics 17, no. 9 (September 2025): 1237. 10.3390/pharmaceutics17091237 PMC12473319 41012571

[392] D. H. Ngwanou , E. Couchonnal , F. Parant , et al., “Long‐Term Urinary Copper Excretion and Exchangeable Copper in Children with Wilson Disease under Chelation Therapy,” Journal of Pediatric Gastroenterology and Nutrition 75, no. 4 (October 2022): e75–e80. 10.1097/MPG.0000000000003531 35706098

[393] M. J. Kim , M. J. Tang , and E. Bruera , “Prolonged Zinc Use for Dysgeusia Causing Copper Deficiency,” Palliative and Supportive Care 24 (January 2026): e37. 10.1017/S1478951525101478 PMC13166446 41539972

[394] Z. W. Myint , T. H. Oo , K. Z. Thein , A. M. Tun , and H. Saeed , “Copper Deficiency Anemia: Review Article,” Annals of Hematology 97, no. 9 (September 2018): 1527–1534. 10.1007/s00277-018-3407-5 29959467

[395] J. A. Shankar , A. Kaur , L. Tyagi , S. Selvam , R. Hans , and A. K. Pannu , “Severe Copper Sulphate Poisoning: A Case Rescued With Plasmapheresis,” Tropical Doctor 53, no. 2 (April 2023): 327–328. 10.1177/00494755231151256 36628444

[396] T. Li and Y. Q. Lu , “Residual Hyperglycemia After Successful Treatment of a Patient With Severe Copper Sulfate Poisoning,” Journal of Zhejiang University‐SCIENCE B 25, no. 12 (December 2024): 1120–1124. 10.1631/jzus.B2400305 PMC11693388 39743299

[397] P. Hedera , “Clinical Management of Wilson Disease,” Annals of Translational Medicine 7, no. Suppl 2 (April 2019): S66. 10.21037/atm.2019.03.18 PMC6531662 31179303

[398] I. Mohr and K. H. Weiss , “Current Anti‐Copper Therapies in Management of Wilson Disease,” Annals of Translational Medicine 7, no. Suppl 2 (April 2019): S69. 10.21037/atm.2019.02.48 PMC6531644 31179306

[399] C. T. Huang , C. Y. Chen , and Y. J. Liang , “Effects of Heyndrickxia Coagulans on Waterborne Copper Toxicity,” Life (Basel) 15, no. 2 (February 2025): 300. 10.3390/life15020300 PMC11857749 40003709

[400] F. T. Kirk , D. E. Munk , E. S. Swenson , et al., “Effects of Tetrathiomolybdate on Copper Metabolism in Healthy Volunteers and in Patients With Wilson Disease,” Journal of Hepatology 80, no. 4 (April 2024): 586–595. 10.1016/j.jhep.2023.11.023 38081365

[401] A. Iqubal , M. K. Iqubal , A. Khan , J. Ali , S. Baboota , and S. E. Haque , “Gene Therapy, a Novel Therapeutic Tool for Neurological Disorders: Current Progress, Challenges and Future Prospective,” Current Gene Therapy 20, no. 3 (2020): 184–194. 10.2174/1566523220999200716111502 32674730

[402] A. Kagiava , X. Chen , R. S. Finkel , S. J. Gray , and K. A. Kleopa , “Progress and Challenges in Intrathecal Gene Therapy for Neurological Disorders,” EBioMedicine 128 (May 2026): 106294. 10.1016/j.ebiom.2026.106294 PMC13196305 42134074

[403] D. M. Hoang , P. T. Pham , T. Q. Bach , et al., “Stem Cell‐Based Therapy for Human Diseases,” Signal Transduction and Targeted Therapy 7, no. 1 (August 2022): 272. 10.1038/s41392-022-01134-4 PMC9357075 35933430

[404] L. Cai , X. K. Li , Y. Song , and M. G. Cherian , “Essentiality, Toxicology and Chelation Therapy of Zinc and Copper,” Current Medicinal Chemistry 12, no. 23 (2005): 2753–2763. 10.2174/092986705774462950 16305470

[405] Y. Haruta , H. Saiwai , K. Kobayakawa , et al., “Zinc Chelation Therapy Mitigates Muscle Fibrosis and Improves Functional Outcomes in a Murine Model of Crush Syndrome,” Journal of Orthopaedic Science (January 2026): 938–948. 10.1016/j.jos.2025.12.009 41617627

[406] A. Duncan , D. Talwar , and I. Morrison , “The Predictive Value of Low Plasma Copper and High Plasma Zinc in Detecting Zinc‐induced Copper Deficiency,” Annals of Clinical Biochemistry: International Journal of Laboratory Medicine 53, no. Pt 5 (September 2016): 575–579. 10.1177/0004563215620821 26586851

[407] A. Duncan , G. Gallacher , and L. Willox , “The Role of the Clinical Biochemist in Detection of Zinc‐Induced Copper Deficiency,” Annals of Clinical Biochemistry: International Journal of Laboratory Medicine 53, no. Pt 2 (March 2016): 298–301. 10.1177/0004563215595429 26113737

[408] Z. Marino and M. L. Schilsky , “Wilson Disease: Novel Diagnostic and Therapeutic Approaches,” Seminars in Liver Disease 45, no. 2 (June 2025): 221–235. 10.1055/a-2460-8999 PMC12962356 39496313

[409] J. W. Chen , T. Zeoli , N. C. Hughes , A. Lane , and R. A. Berkman , “Copper Deficiency Myelopathy Mimicking Cervical Spondylitic Myelopathy: A Systematic Review of the Literature With Case Report,” The Spine Journal 24, no. 11 (November 2024): 2026–2034. 10.1016/j.spinee.2024.06.018 38909910

[410] O. AlDhasee , H. AlMalki , N. AlKharashi , N. AlJeraisy , and M. Al Deeb , “Acute Zinc Sulfate Overdose: Clinical Presentation and Management,” BMJ Case Reports 18, no. 1 (January 2025): e263899. 10.1136/bcr-2024-263899 39828318

[411] I. S. deSouza , R. Allen , P. Shrestha , H. Thode, Jr. , J. Koos , and A. Singer , “Efficacy and Safety of Pharmacologic Therapies for Nausea and Emesis in the Emergency Department: A Systematic Review and Bayesian Network Meta‐Analysis,” Annals of Emergency Medicine 86, no. 6 (December 2025): 646–658. 10.1016/j.annemergmed.2025.06.009 40772912

[412] M. Jozwiak‐Bebenista and J. Z. Nowak , “Paracetamol: Mechanism of Action, Applications and Safety Concern,” Acta Poloniae Pharmaceutica 71, no. 1 (January‐February 2014): 11–23. 24779190

[413] A. Girao , J. Abreu Fernandes , L. Valente , C. Macedo , and R. Pina , “Metal Fume Fever: An Underdiagnosis,” Cureus 15, no. 2 (February 2023): e34928. 10.7759/cureus.34928 PMC9976638 36874333

[414] R. G. Goldie , K. M. Lulich , and J. W. Paterson , “Bronchodilators: Β‐Agonists,” Medical Journal of Australia 162, no. 2 (January 1995): 100–102. 10.5694/j.1326-5377.1995.tb138446.x 7838013

[415] K. P. Manji , S. Somji , M. Bakari , et al., “Efficacy of Zinc Supplementation for Young Infants With Clinical Severe Infection in Tanzania: Study Protocol for a Randomised Controlled Trial,” BMJ Paediatrics Open 9, no. 1 (August 2025): e003804. 10.1136/bmjpo-2025-003804 PMC12352140 40813142

[416] H. Zhu , H. Zhang , F. Jin , et al., “Elevated ORAI1 Expression Mediates Tumor‐Promoting Intracellular Ca2+ Oscillations in Human Esophageal Squamous Cell Carcinoma,” Oncotarget 5, no. 11 (June 2014): 3455–3471. 10.18632/oncotarget.1903 PMC4116495 24797725

[417] H. Yuan , J. Xu , Y. Zhu , et al., “Activation of Calcium‑Sensing Receptor‑Mediated Autophagy in High Glucose‑Induced Cardiac Fibrosis in Vitro,” Molecular Medicine Reports 22, no. 3 (September 2020): 2021–2031. 10.3892/mmr.2020.11277 PMC7411369 32705187

[418] Z. Pan and J. Ma , “Open Sesame: Treasure in Store‐Operated Calcium Entry Pathway for Cancer Therapy,” Science China Life Sciences 58, no. 1 (January 2015): 48–53. 10.1007/s11427-014-4774-3 PMC4765918 25481035

[419] X. Ding , Z. He , K. Zhou , et al., “Essential Role of TRPC6 Channels in G2/M Phase Transition and Development of Human Glioma,” JNCI: Journal of the National Cancer Institute 102, no. 14 (July 2010): 1052–1068. 10.1093/jnci/djq217 20554944

[420] T. Boczek , M. Sobolczyk , J. Mackiewicz , et al., “Crosstalk Among Calcium Atpases: PMCA, SERCA and SPCA in Mental Diseases,” International Journal of Molecular Sciences 22, no. 6 (March 2021): 2785. 10.3390/ijms22062785 PMC8000800 33801794

[421] H. O. Gu , S. W. Noh , O. H. Kim , and B. C. Oh , “Crucial Roles of Calcium Atpases and Phosphoinositides: Insights Into Pathophysiology and Therapeutic Strategies,” Molecules and Cells 48, no. 9 (September 2025): 100254. 10.1016/j.mocell.2025.100254 PMC12329087 40653168

[422] C. Cui , R. Merritt , L. Fu , and Z. Pan , “Targeting Calcium Signaling in Cancer Therapy,” Acta Pharmaceutica Sinica B 7, no. 1 (January 2017): 3–17. 10.1016/j.apsb.2016.11.001 PMC5237760 28119804

[423] S. Baruah , M. K. Bharali , N. Akhtara , et al., “Thapsigargin Enhanced Chemotherapeutic Sensitivity of Irinotecan in the Inflammation‐Induced Colorectal Cancer Model in Mice,” Scientific Reports 16, no. 1 (January 2026): 4551. 10.1038/s41598-025-34567-2 PMC12868824 41491020

[424] J. T. Isaacs , W. N. Brennen , S. B. Christensen , and S. R. Denmeade , “Mipsagargin: The Beginning—Not the End—Of Thapsigargin Prodrug‐Based Cancer Therapeutics,” Molecules 26, no. 24 (December 2021): 7469. 10.3390/molecules26247469 PMC8707208 34946547

[425] R. Cui , Y. Wang , L. Wang , et al., “Cyclopiazonic Acid, an Inhibitor of Calcium‐Dependent Atpases With Antiviral Activity Against Human respiratory Syncytial Virus,” Antiviral Research 132 (August 2016): 38–45. 10.1016/j.antiviral.2016.05.010 27210812

[426] M. Omote and H. Mizusawa , “Endothelium‐Dependent Rhythmic Contractions Induced by Cyclopiazonic Acid, a Sarcoplasmic Reticulum Ca^2+^ ‐Pump Inhibitor, in the Rabbit Femoral Artery,” Acta Physiologica Scandinavica 154, no. 2 (June 1995): 113–119. 10.1111/j.1748-1716.1995.tb09893.x 7572207

[427] B. Yang , M. Zhang , J. Gao , et al., “Small Molecule Rl71 Targets SERCA2 at a Novel Site in the Treatment of Human Colorectal Cancer,” Oncotarget 6, no. 35 (November 2015): 37613–37625. 10.18632/oncotarget.6068 PMC4741952 26608678

[428] A. Muscella , N. Calabriso , C. Vetrugno , et al., “The Platinum (II) Complex [Pt(O,O′‐ACAC)(γ‐ACAC)(DMS)] Alters the Intracellular Calcium Homeostasis in MCF‐7 Breast Cancer Cells,” Biochemical Pharmacology 81, no. 1 (January 2011): 91–103. 10.1016/j.bcp.2010.09.012 20854797

[429] M. Li , W. Zhang , X. Yang , G. An , and W. Zhao , “The α2δ1 Subunit of the Voltage‐gated Calcium Channel Acts as a Potential Candidate for Breast Cancer Tumor Initial Cells Biomarker,” Cancer Biomarkers 31, no. 3 (2021): 295–305. 10.3233/CBM-203165 PMC12500007 33896833

[430] A. Shiozaki , K. Kurashima , M. Kudou , et al., “Cancer Stem Cells of Hepatocellular Carcinoma Are Suppressed by the Voltage‐gated Calcium Channel Inhibitor Amlodipine,” Anticancer Research 43, no. 11 (November 2023): 4855–4864. 10.21873/anticanres.16682 37909988

[431] S.‐H. Gao , G.‐Z. Wang , L. I.‐P. Wang , et al., “Mutations and Clinical Significance of Calcium Voltage‐gated Channel Subunit Alpha 1e (CACNA1E) in Non‐Small Cell Lung Cancer,” Cell Calcium 102 (March 2022): 102527. 10.1016/j.ceca.2022.102527 35026540

[432] R. Khanam , I. S. Fanous , E. N. Fadhel , T. Hyder , and A. Brufsky , “Voltage‐Gated Calcium Channel Antibody‐Induced Oropharyngeal Dysphagia Presenting as a Paraneoplastic Neurological Complication in Breast Cancer,” Cureus 13, no. 3 (March 2021): e13677. 10.7759/cureus.13677 PMC8012257 33824828

[433] V. P. Kale , S. G. Amin , and M. K. Pandey , “Targeting Ion Channels for Cancer Therapy by Repurposing the Approved Drugs,” Biochimica et Biophysica Acta (BBA)—Biomembranes 1848, no. 10 Pt B (October 2015): 2747–2755. 10.1016/j.bbamem.2015.03.034 25843679

[434] J. M. Taylor and R. U. Simpson , “Inhibition of Cancer Cell Growth by Calcium Channel Antagonists in the Athymic Mouse,” Cancer Research 52, no. 9 (May 1992): 2413–2418. 1533173

[435] J. Yoshida , T. Ishibashi , and M. Nishio , “G1 Cell Cycle Arrest by Amlodipine, a Dihydropyridine Ca2+ Channel Blocker, in Human Epidermoid Carcinoma A431 Cells,” Biochemical Pharmacology 73, no. 7 (Apri; 2007): 943–953. 10.1016/j.bcp.2006.12.011 17217918

[436] C. J. Dube , Y. Zhang , S. Saha , et al., “Microenvironment T‐type Calcium Channels Regulate Neuronal and Glial Processes in Tumor Cells to Promote Glioblastoma Growth,” Neuro‐Oncology 28, no. 9 (May 2026): 2123–2135. 10.1093/neuonc/noag131 PMC13550691 42218829

[437] I. Garrido‐Laguna , A. C. Tan , M. C. Villarroel , et al., “Activity of the T‐Type Calcium Channel Antagonist Mibefradil in Pancreatic Cancer Xenografts,” Clinical Cancer Research 14, no. 19 (2008): B49.

[438] A. J. Krouse , L. Gray , T. Macdonald , and J. McCray , “Repurposing and Rescuing of Mibefradil, an Antihypertensive, for Cancer: A Case Study,” ASSAY and Drug Development Technologies 13, no. 10 (December 2015): 650–653. 10.1089/adt.2015.29014.ajkdrrr 26690767

[439] K. I. H. Kim , D. Kim , J. U. Y. Park , et al., “NNC 55‐0396, a T‐Type Ca2+ Channel Inhibitor, Inhibits Angiogenesis via Suppression of Hypoxia‐Inducible Factor‐1α Signal Transduction,” Journal of Molecular Medicine 93, no. 5 (May 2015): 499–509. 10.1007/s00109-014-1235-1 25471482

[440] H.‐K. Rim , H.‐W. Lee , I. L. S. Choi , et al., “T‐Type Ca2+ Channel Blocker, KYS05047 Induces G1 Phase Cell Cycle Arrest by Decreasing Intracellular Ca2+ Levels in human Lung Adenocarcinoma a549 Cells,” Bioorganic & Medicinal Chemistry Letters 22, no. 23 (December 2012): 7123–7126. 10.1016/j.bmcl.2012.09.076 23079520

[441] N. Díaz‐Lezama , M. Hernández‐Elvira , A. Sandoval , A. Monroy , R. Felix , and E. Monjaraz , “Ghrelin Inhibits Proliferation and Increases T‐Type Ca2+ Channel Expression in Pc‐3 Human Prostate Carcinoma Cells,” Biochemical and Biophysical Research Communications 403, no. 1 (December 2010): 24–29. 10.1016/j.bbrc.2010.10.100 21040709

[442] J. E. Merritt , W. P. Armstrong , C. D. Benham , et al., “SK&F 96365, a Novel Inhibitor of Receptor‐Mediated Calcium Entry,” Biochemical Journal 271, no. 2 (October 1990): 515–522. 10.1042/bj2710515 PMC1149585 2173565

[443] B. O. Zeng , C. Yuan , X. Yang , S. L Atkin , and S.‐Z. Xu , “Trpc Channels and Their Splice Variants Are Essential for Promoting human Ovarian Cancer Cell Proliferation and Tumorigenesis,” Current Cancer Drug Targets 13, no. 1 (January 2013): 103–116. 22920441

[444] M. Prakriya and R. S. Lewis , “Potentiation and Inhibition of Ca^2+^ Release‐Activated Ca^2+^ Channels by 2‐Aminoethyldiphenyl Borate (2‐APB) Occurs Independently of IP_3_ Receptors,” The Journal of Physiology 536, no. Pt 1 (October 2001): 3–19. 10.1111/j.1469-7793.2001.t01-1-00003.x PMC2278849 11579153

[445] F. Splettstoesser , A.‐M. Florea , and D. Büsselberg , “IP_3_ Receptor Antagonist, 2‐APB, Attenuates Cisplatin Induced Ca^2+^ ‐Influx in HeLa‐S3 Cells and Prevents Activation of Calpain and Induction of Apoptosis,” British Journal of Pharmacology 151, no. 8 (August 2007): 1176–1186. 10.1038/sj.bjp.0707335 PMC2189830 17592515

[446] J.‐I. Goto , A. Z. Suzuki , S. Ozaki , et al., “Two Novel 2‐aminoethyl Diphenylborinate (2‐APB) Analogues Differentially Activate and Inhibit Store‐Operated Ca2+ Entry via STIM Proteins,” Cell Calcium 47, no. 1 (January 2010): 1–10. 10.1016/j.ceca.2009.10.004 PMC2905153 19945161

[447] D. M. Haverstick , T. N. Heady , T. L. Macdonald , and L. S. Gray , “Inhibition of Human Prostate Cancer Proliferation in Vitro and in a Mouse Model by a Compound Synthesized to Block Ca2+ Entry,” Cancer Research 60, no. 4 (February 2000): 1002–1008. 10706116

[448] C. P. Landowski , K. A. Bolanz , Y. Suzuki , and M. A. Hediger , “Chemical Inhibitors of the Calcium Entry Channel TRPV6,” Pharmaceutical Research 28, no. 2 (February 2011): 322–330. 10.1007/s11095-010-0249-9 21057859

[449] S. Fu , H. Hirte , S. Welch , et al., “First‐in‐Human Phase I Study of SOR‐c13, a TRPV6 Calcium Channel Inhibitor, in Patients With Advanced Solid Tumors,” Investigational New Drugs 35, no. 3 (June 2017): 324–333. 10.1007/s10637-017-0438-z PMC5418314 28150073

[450] K. S. Thorneloe , A. C. Sulpizio , Z. Lin , et al., “N‐((1s)‐1‐[4‐((2s)‐2‐[(2,4‐Dichlorophenyl)Sulfonyl]Amino‐3‐Hydroxypropanoyl)‐1‐Piperazinyl]Carbonyl‐3‐Methylbutyl)‐1‐Benzothiophene‐2‐Carboxamide (GSK1016790A), a Novel and Potent Transient Receptor Potential Vanilloid 4 Channel Agonist Induces Urinary Bladder Contraction and Hyperactivity: Part i,” Journal of Pharmacology and Experimental Therapeutics 326, no. 2 (August 2008): 432–442. 10.1124/jpet.108.139295 18499743

[451] M. Cheung , W. Bao , D. J. Behm , et al., “Discovery of Gsk2193874: An Orally Active, Potent, and Selective Blocker of Transient Receptor Potential Vanilloid 4,” ACS Medicinal Chemistry Letters no. 5 (May 2017): 549–554. 10.1021/acsmedchemlett.7b00094 PMC5430398 28523109

[452] F. Yang , X. Xiao , W. Cheng , et al., “Structural Mechanism Underlying Capsaicin Binding and Activation of the TRPV1 Ion Channel,” Nature Chemical Biology 11, no. 7 (July 2015): 518–524. 10.1038/nchembio.1835 PMC4472570 26053297

[453] A. M. Sánchez , M. G. Sánchez , S. Malagarie‐Cazenave , N. Olea , and I. Díaz‐Laviada , “Induction of Apoptosis in Prostate Tumor PC‐3 Cells and Inhibition of Xenograft Prostate Tumor Growth by the Vanilloid Capsaicin,” Apoptosis 11, no. 1 (January 2006): 89–99. 10.1007/s10495-005-3275-z 16374544

[454] J. K. Lau , K. C. Brown , A. M. Dom , et al., “Capsaicin Induces Apoptosis in Human Small Cell Lung Cancer via the TRPV6 Receptor and the Calpain Pathway,” Apoptosis 19, no. 8 (August 2014): 1190–1201. 10.1007/s10495-014-1007-y PMC4072851 24878626

[455] C. B. Gonzales , N. B. Kirma , J. J. De La Chapa , et al., “Vanilloids Induce Oral Cancer Apoptosis Independent of TRPV1,” Oral Oncology 50, no. 5 (May 2014): 437–447. 10.1016/j.oraloncology.2013.12.023 PMC4418484 24434067

[456] C. Carson , P. Raman , J. Tullai , et al., “Englerin a Agonizes the TRPC4/C5 Cation Channels to Inhibit Tumor Cell Line Proliferation,” PLoS ONE 10, no. 6 (2015): e0127498. 10.1371/journal.pone.0127498 PMC4476799 26098886

[457] Y. Akbulut , H. J. Gaunt , K. Muraki , et al., “(−)‐Englerin A Is a Potent and Selective Activator of TRPC4 and TRPC5 Calcium Channels,” Angewandte Chemie International Edition 54, no. 12 (March 2015): 3787–3791. 10.1002/anie.201411511 PMC7116557 25707820

[458] J. A. H. Hwang , M. K. Hwang , Y. Jang , et al., “20‐O‐β‐D‐Glucopyranosyl‐20(S)‐Protopanaxadiol, a Metabolite of Ginseng, Inhibits Colon Cancer Growth by Targeting TRPC Channel‐Mediated Calcium Influx,” The Journal of Nutritional Biochemistry 24, no. 6 (June 2013): 1096–1104. 10.1016/j.jnutbio.2012.08.008 23318138

[459] B. Costa , G. Giagnoni , C. Franke , A. E. Trovato , and M. Colleoni , “Vanilloid TRPV1 Receptor Mediates the Antihyperalgesic Effect of the Nonpsychoactive Cannabinoid, Cannabidiol, in a Rat Model of Acute Inflammation,” British Journal of Pharmacology 143, no. 2 (September 2004): 247–250. 10.1038/sj.bjp.0705920 PMC1575333 15313881

[460] R. Ramer , K. Bublitz , N. Freimuth , et al., “Cannabidiol Inhibits Lung Cancer Cell Invasion and Metastasis via Intercellular Adhesion Molecule‐1,” The FASEB Journal 26, no. 4 (April 2012): 1535–1548. 10.1096/fj.11-198184 22198381

[461] B. A. McNally , A. Somasundaram , M. Yamashita , and M. Prakriya , “Gated Regulation of CRAC Channel Ion Selectivity by STIM1,” Nature 482, no. 7384 (January 2012): 241–245. 10.1038/nature10752 PMC3276717 22278058

[462] A. V. Yeromin , S. L. Zhang , W. Jiang , Y. Yu , O. Safrina , and M. D. Cahalan , “Molecular Identification of the CRAC Channel by Altered Ion Selectivity in a Mutant of orai,” Nature 443, no. 7108 (September 2006): 226–229. 10.1038/nature05108 PMC2756048 16921385

[463] M. Trebak , G. St J Bird , R. R. McKay , and J. W. Putney , “Comparison of Human TRPC3 Channels in Receptor‐Activated and Store‐Operated Modes,” Journal of Biological Chemistry 277, no. 24 (June 2002): 21617–21623. 10.1074/jbc.M202549200 11943785

[464] S. Yang , J. J. Zhang , and X. Y. Huang , “Orai1 and stim1 Are Critical for Breast Tumor Cell Migration and Metastasis,” Cancer Cell 15, no. 2 (February 2009): 124–134. 10.1016/j.ccr.2008.12.019 19185847

[465] L. P. He , T. Hewavitharana , J. Soboloff , M. A. Spassova , and D. L. Gill , “A Functional Link Between Store‐Operated and TRPC Channels Revealed by the 3,5‐bis(Trifluoromethyl)Pyrazole Derivative, BTP2,” Journal of Biological Chemistry 280, no. 12 (March 2005): 10997–11006. 10.1074/jbc.M411797200 15647288

[466] T. Yoshino , J. Ishikawa , K. Ohga , et al., “Ym‐58483, a Selective CRAC Channel Inhibitor, Prevents Antigen‐Induced Airway Eosinophilia and Late Phase Asthmatic Responses via TH2 Cytokine Inhibition in Animal Models,” European Journal of Pharmacology 560, no. 2‐3 (April 2007): 225–233. 10.1016/j.ejphar.2007.01.012 17307161

[467] R. Takezawa , H. Cheng , A. Beck , et al., “A Pyrazole Derivative Potently Inhibits Lymphocyte Ca2+ Influx and Cytokine Production by Facilitating Transient Receptor Potential Melastatin 4 Channel Activity,” Molecular Pharmacology 69, no. 4 (April 2006): 1413–1420. 10.1124/mol.105.021154 16407466

[468] A. M. Sadaghiani , S. M. Lee , J. I. Odegaard , et al., “Identification of ORAI1 Channel Inhibitors by Using Minimal Functional Domains to Screen Small Molecule Microarrays,” Chemistry & Biology 21, no. 10 (October 2014): 1278–1292. 10.1016/j.chembiol.2014.08.016 25308275

[469] J. T. Smyth , W. I. Dehaven , G. S. Bird , and J. W. Putney Jr. , “Ca2+‐Store‐Dependent and ‐Independent Reversal of STIM1 Localization and Function,” Journal of Cell Science 121, no. Pt 6 (March 2008): 762–772. 10.1242/jcs.023903 PMC2587154 18285445

[470] A. Kondratskyi , M. Yassine , C. Slomianny , et al., “Identification of Ml‐9 as a Lysosomotropic Agent Targeting Autophagy and Cell Death,” Cell Death & Disease 5, no. 4 (April 2014): e1193. 10.1038/cddis.2014.156 PMC4001310 24763050

[471] G. Chen , S. Panicker , K.‐Y. Lau , et al., “Characterization of a Novel Crac Inhibitor That Potently Blocks Human T Cell Activation and Effector Functions,” Molecular Immunology 54, no. 3 (2013): 355–367. 10.1016/j.molimm.2012.12.011 23357789

[472] A. Jairaman and M. Prakriya , “Molecular Pharmacology of Store‐Operated Crac Channels,” Channels 7, no. 5 (September‐October 2013): 402–414. 10.4161/chan.25292 PMC3913763 23807116

[473] I. Derler , R. Schindl , R. Fritsch , et al., “The Action of Selective CRAC Channel Blockers Is Affected by the Orai Pore Geometry,” Cell Calcium 53, no. 2 (February 2013): 139–151. 10.1016/j.ceca.2012.11.005 PMC3580291 23218667

[474] S. Ramos , R. E. GS , J. Roos , J. Whitten , K. Stauderman , and G. Velicelebi , “Cm3457, a Potent and Selective Oral Crac Channel Inhibitor, Suppresses T and Mast Cell Function and Is Efficacious in Rat Models of Arthritis and Asthma,” The Journal of Immunology 188, no. Issue 1_Supplement (2012): 72.73.

[475] F.‐F. Lin , R. Elliott , A. Colombero , et al., “Generation and Characterization of Fully human Monoclonal Antibodies Against human Orai1 for Autoimmune Disease,” The Journal of Pharmacology and Experimental Therapeutics 345, no. 2 (May 2013): 225–238. 10.1124/jpet.112.202788 23475901

[476] K. Gaida , H. Salimi‐Moosavi , R. Subramanian , et al., “Inhibition of Crac With a Human Anti‐Orai1 Monoclonal Antibody Inhibits T‐Cell‐Derived Cytokine Production but Fails to Inhibit a T‐Cell‐Dependent Antibody Response in the Cynomolgus Monkey,” Journal of Immunotoxicology 12, no. 2 (April‐June 2015): 164–173. 10.3109/1547691X.2014.915897 24990272

[477] J. H. Cox , S. Hussell , H. Søndergaard , et al., “Antibody‐Mediated Targeting of the Orai1 Calcium Channel Inhibits T Cell Function,” PLoS ONE 8, no. 12 (2013): e82944. 10.1371/journal.pone.0082944 PMC3871607 24376610

[478] S. Miyamoto , M. Izumi , M. Hori , M. Kobayashi , H. Ozaki , and H. Karaki , “Xestospongin C, a Selective and Membrane‐Permeable Inhibitor of IP_3_ Receptor, Attenuates the Positive Inotropic Effect of α‐Adrenergic Stimulation in Guinea‐Pig Papillary Muscle,” British Journal of Pharmacology 130, no. 3 (June 2000): 650–654. 10.1038/sj.bjp.0703358 PMC1572115 10821794

[479] E. Jaimovich , C. Mattei , J. L. Liberona , et al., “Xestospongin B, a Competitive Inhibitor of IP_3_ ‐Mediated Ca^2+^ Signalling in Cultured Rat Myotubes, Isolated Myonuclei, and Neuroblastoma (NG108‐15) Cells,” FEBS Letters 579, no. 10 (April 2005): 2051–2057. 10.1016/j.febslet.2005.02.053 15811317

[480] C. Sakakura , A. Hagiwara , K. Fukuda , et al., “Possible Involvement of Inositol 1,4,5‐Trisphosphate Receptor Type 3 (IP3R3) in the Peritoneal Dissemination of Gastric Cancers,” Anticancer Research 23, no. 5A (September‐October 2003): 3691–3697. 14666665

[481] Z. I. Khamis , K. A. Iczkowski , Y. G. Man , M. J. Bou‐Dargham , and Q. X. Sang , “Evidence for a Proapoptotic Role of Matrix Metalloproteinase‐26 in Human Prostate Cancer Cells and Tissues,” Journal of Cancer 7, no. 1 (2016): 80–87. 10.7150/jca.13067 PMC4679384 26722363

[482] C. Garcia‐Prieto , K. B. Riaz Ahmed , Z. Chen , et al., “Effective Killing of Leukemia Cells by the Natural Product Osw‐1 Through Disruption of Cellular Calcium Homeostasis,” Journal of Biological Chemistry 288, no. 5 (February 2013): 3240–3250. 10.1074/jbc.M112.384776 PMC3561545 23250754

[483] M. Song , D. Chen , and S. P. Yu , “The TRPC Channel Blocker SKF 96365 Inhibits Glioblastoma Cell Growth by Enhancing Reverse Mode of the Na^+^/Ca^2+^ Exchanger and Increasing Intracellular Ca^2+^ ,” British Journal of Pharmacology 171, no. 14 (July 2014): 3432–3447. 10.1111/bph.12691 PMC4105931 24641279

[484] J. K. Ryu , N. Jantaratnotai , M. C. Serrano‐Perez , P. L. McGeer , and J. G. McLarnon , “Block of Purinergic P2X_7_ R Inhibits Tumor Growth in a C6 Glioma Brain Tumor Animal Model,” Journal of Neuropathology & Experimental Neurology 70, no. 1 (January 2011): 13–22. 10.1097/NEN.0b013e318201d4d4 21157381

[485] R. Bartlett , L. Stokes , and R. Sluyter , “The P2X7 Receptor Channel: Recent Developments and the Use of P2X7 Antagonists in Models of Disease,” Pharmacological Reviews 66, no. 3 (July 2014): 638–675. 10.1124/pr.113.008003 24928329

[486] J. Zhang , T. Ju , Y. Qin , M. Hou , L. Gao , and L. A. Wu , “Therapeutic Targeting of p2x Receptors for Orofacial Pain,” Brain Research Bulletin 224 (May 2025): 111301. 10.1016/j.brainresbull.2025.111301 40086763

[487] A. Giannuzzo , S. F. Pedersen , and I. Novak , “The P2X7 Receptor Regulates Cell Survival, Migration and Invasion of Pancreatic Ductal Adenocarcinoma Cells,” Molecular Cancer 14 (November 2015): 203. 10.1186/s12943-015-0472-4 PMC4660609 26607222

[488] H. Jin , S. O. Y. Eun , J. S. Lee , et al., “P2y2 Receptor Activation by Nucleotides Released From Highly Metastatic Breast Cancer Cells Increases Tumor Growth and Invasion via Crosstalk With Endothelial Cells,” Breast Cancer Research 16, no. 5 (August 2014): R77. 10.1186/bcr3694 PMC4406012 25156554

[489] R. Xie , J. Xu , G. Wen , et al., “The p2y2 Nucleotide Receptor Mediates the Proliferation and Migration of Human Hepatocellular Carcinoma Cells Induced by Atp,” Journal of Biological Chemistry 289, no. 27 (July 2014): 19137–19149. 10.1074/jbc.M113.540047 PMC4081950 24847054

[490] G. Nylund , L. Hultman , S. Nordgren , and D. S. Delbro , “P2Y_2_ ‐ and P2Y_4_ Purinergic Receptors Are Over‐Expressed in Human Colon Cancer,” Autonomic and Autacoid Pharmacology 27, no. 2 (April 2007): 79–84. 10.1111/j.1474-8673.2007.00389.x 17391276

[491] J. P. Maynard , J. U.‐S. Lee , B. O. H. Sohn , et al., “P2X3 Purinergic Receptor Overexpression Is Associated With Poor Recurrence‐Free Survival in Hepatocellular Carcinoma Patients,” Oncotarget 6, no. 38 (December 2015): 41162–41179. 10.18632/oncotarget.6240 PMC4747397 26517690

